# Infant mortality and growth failure after oral azithromycin among low birthweight and underweight neonates: A subgroup analysis of a randomized controlled trial

**DOI:** 10.1371/journal.pgph.0001009

**Published:** 2023-05-15

**Authors:** Mamadou Bountogo, Ali Sié, Alphonse Zakane, Guillaume Compaoré, Thierry Ouédraogo, Jessica Brogdon, Elodie Lebas, Fanice Nyatigo, Melissa M. Medvedev, Benjamin F. Arnold, Thomas M. Lietman, Catherine E. Oldenburg, for the NAITRE Study Team

**Affiliations:** 1 Centre de Recherche en Santé de Nouna, Nouna, Burkina Faso; 2 Francis I Proctor Foundation, University of California, San Francisco, San Francisco, California, United States of America; 3 Institute for Global Health Sciences, University of California, San Francisco, San Francisco, California, United States of America; 4 Department of Pediatrics, University of California, San Francisco, San Francisco, California, United States of America; 5 Maternal, Adolescent, Reproductive & Child Health Centre, London School of Hygiene & Tropical Medicine, London, United Kingdom; 6 Department of Ophthalmology, University of California, San Francisco, San Francisco, California, United States of America; 7 Department of Epidemiology & Biostatistics, University of California, San Francisco, San Francisco, California, United States of America; PLOS: Public Library of Science, UNITED STATES

## Abstract

**Background:**

Low birthweight (birthweight <2500 grams, g) and underweight (weight-for-age Z-score, WAZ, < -2) infants have higher risk of poor outcomes compared to their well-nourished peers. We evaluated the role of azithromycin for reducing mortality and improving growth outcomes in low birthweight and/or underweight infants.

**Methods:**

Infants aged 8–27 days of age weighing ≥2500 g at enrollment in Burkina Faso were randomized 1:1 to a single, oral dose of azithromycin (20 mg/kg) or matching placebo. We evaluated mortality and anthropometric outcomes in four subgroups: 1) both low birthweight and underweight at enrollment; 2) low birthweight-only; 3) underweight-only; 4) neither low birthweight nor underweight.

**Findings:**

Of 21,832 enrolled infants, 21,320 (98%) had birthweight measurements and included in this analysis. Of these, 747 (3%) were both low birthweight and underweight, 972 (5%) were low birthweight-only, 825 (4%) were underweight-only, and 18,776 (88%) were neither low birthweight nor underweight. Infants who were both low birthweight and underweight receiving azithromycin had lower odds of underweight at 6 months compared to placebo (OR 0.65, 95% CI 0.44 to 0.95), but the treatment group by subgroup interaction was not statistically significant (*P* = 0.06). We did not find evidence of a difference between groups for other outcomes in any subgroup.

**Interpretation:**

Azithromycin may have some growth-promoting benefits for the highest risk infants, but we were unable to demonstrate a difference in most outcomes in low birthweight and underweight infants. As a secondary analysis of a trial, this study was underpowered for rare outcomes such as mortality.

**Trial registration:**

ClinicalTrials.gov NCT03682653.

## Introduction

Low birthweight (birthweight < 2500 grams, g) and underweight (weight-for-age Z-score, WAZ, < -2 SD) neonates are at increased risk of mortality, morbidity, and growth failure compared to term, appropriate-for-gestational age babies [1]. An estimated 2.4 million neonatal deaths occurred in 2019, accounting for 47% of deaths among children under 5 years of age [2]. More than 80% of these deaths occur in babies with a low birthweight who are small due to being preterm, small for their gestational age, or both [1]. Early life interventions to prevent neonatal mortality, especially among small newborns, may help reduce the excess burden of mortality that is persistent in this age group [3].

Recent cohort analyses of wasting and stunting have shown that the highest incidence of growth failure occurs before 6 months of age. Children experiencing growth failure during this period are at the highest risk of severe consequences, including persistent growth failure and mortality [4, 5]. Although interventions that include nutritional supplementation, such as lipid-based nutrient supplements (LNS), have been shown to reduce mortality and improve weight gain in older children, their utility is limited in infants under 6 months for whom exclusive breastfeeding should be promoted [6–9]. Infants under 6 months of age with growth failure are a key population in the current World Health Organization guideline development for prevention and treatment of wasting in children [10]. Interventions for infants at high risk of wasting that do not interfere with breastfeeding are therefore of high priority. Antibiotics are routinely administered to infants over 6 months of age with severe acute malnutrition (weight-for-height Z-score < -3 mid-upper arm circumference < 11.5 cm) [11]. Children <6 months of age with severe acute malnutrition are typically managed on an inpatient basis, and a broad-spectrum antibiotic (e.g., amoxicillin) is recommended for those who are managed as outpatients. However, there is very little evidence to support this recommendation, and the evidence for providing antibiotics to children aged 6 to 59 months is mixed [11–14].

Azithromycin has been shown to reduce all-cause childhood mortality among children aged 1–59 months following biannual mass distribution [15]. The largest effects on mortality were among children aged 1–5 months, who also had the highest mortality rates. The *Nouveux-nés et Azithromycine*: *une Innovation dans le Traitement des Enfants* (NAITRE) study was a randomized controlled trial designed to evaluate whether a single oral dose of azithromycin reduces all-cause infant mortality when administered during the neonatal period (between 8–27 days) compared to placebo [16]. The trial collected data both on vital status and anthropometric endpoints. The primary analysis of found no evidence of an effect of azithromycin on mortality, although the study was underpowered due to lower than anticipated mortality [17]. Any effect of azithromycin on mortality or growth endpoints may be greater in higher risk subgroups. Here, we report the results of a pre-specified subgroup analysis assessing the effects of azithromycin administered to low birthweight and/or underweight neonates on outcomes at 6 months of age.

## Methods

### General study design and study setting

Complete methods for the NAITRE study have been previously reported (**S1 File**) [16]. In brief, neonates aged 8 to 27 days were randomized in a 1:1 fashion to a single oral 20 mg/kg dose of azithromycin or matching placebo. The primary outcome of the trial was all-cause mortality at age 6 months. Participants were enrolled at 44 primary healthcare facilities in 5 regions of Burkina Faso from April 15, 2019 through December 20, 2020. The original trial was designed around detecting infantile hypertrophic pyloric stenosis (IHPS), a rare but serious condition that requires prompt surgical intervention [16, 18]. Macrolides, including azithromycin, are hypothesized to increase the risk of IHPS when administered during the neonatal period [19]. Diagnosis requires an ultrasound, and thus participating primary healthcare facilities had to be geolocated within an hour of a district hospital with ultrasound capacity and within four hours of a hospital with pediatric surgical capacity (in the cities of Ouagadougou or Bobo Dioulasso). Therefore, most enrollment facilities were located in urban settings. The study was reviewed and approved by the Comité d’Ethique pour la Recherche en Santé in Ouagadougou, Burkina Faso (Protocol #2018-10-123) and the Institutional Review Board at the University of California, San Francisco (Protocol #18–25027). Written informed consent was obtained from at least one parent/guardian of each enrolled infant.

### Participants

Neonates were eligible for inclusion in the parent trial if they were between 8 and 27 days of age, weighed ≥2500 g at the time of enrollment, were able to feed orally, and had appropriate caregiver consent. Neonates who were low birthweight were eligible for inclusion if they gained enough weight to meet the 2500 g inclusion criterion by 27 days of age (upper age bound for inclusion in the trial). Caregivers of newborns at participating facilities who were attending routine vaccination days or attending another well-child visit were informed of the study and how to participate. Enrolled participants were clinically stable and children who required hospitalization were not considered for the trial.

### Intervention and randomization

Participants were randomized in a 1:1 fashion to a single oral 20 mg/kg dose of azithromycin or equivalent volume of matching placebo (Pfizer, Inc, New York, NY). Dosing was weight-based, and treatments were delivered via oral syringe. All study treatments consisted of a single dose and were administered and recorded in the study’s mobile electronic data collection application by the enrolling study nurse. The study dose was identical to that used for mass drug administration in trachoma control programs and that is currently being considered in childhood survival programs based on the MORDOR study [15].

### Anthropometric assessments

Anthropometric measurements were collected at enrollment and 6 months of age from all enrolled infants. Children were weighed with a standard infant scale (ADE M112600 U Scale) and measured using a ShorrBoard (Weight and Measure, LLC, Olney, MD). Mid-upper arm circumference (MUAC) was measured with a standard MUAC tape (Weight and Measure, LLC). Length measurements were collected in triplicate and the median was used for analysis. Children with MUAC measurements <11.5 cm at the 6-month study visit were referred to the health facility’s nutritional program for diagnosis and treatment of severe acute malnutrition. At baseline, birthweight was extracted from the child’s government-issued *carnet de santé*, in which various data related to the pregnancy and birth are recorded. This measurement was used to define subgroups based on low birthweight, and the baseline weight measurement collected in the trial was used to defined subgroups based on underweight.

### Outcomes

Vital status was assessed at each follow-up timepoint (21 days after treatment and at 3 and 6 months of age). Follow-up visits were conducted in person or via phone call, with the exception of the 6-month visit, which were in person for anthropometric measurements. Weight gain in g/day and length change in mm/day were calculated at 6 months of age since enrollment. Z-scores, including weight-for-age (WAZ), weight-for-length (WLZ), and length-for-age (LAZ), were calculated at enrollment and 6 months based on 2006 World Health Organization standards [20]. Subgroup sizes vary slightly for mortality and vital status endpoints as mortality was measured over the 6-month study period whereas anthropometry was only measured at the 6-month study visit. Infants who died or were lost to follow-up before 6 months therefore did not have 6-month anthropometric measurements. Per our pre-specified analysis plan, participants outside of WHO child growth standards for WAZ (-6 to +5 SD), LAZ (-6 to +6 SD), and WLZ (-5 to +5 SD) were excluded from all anthropometric analyses.

### Sample size

The trial’s target enrollment was based on the primary endpoint, all-cause mortality at 6 months of age [16]. For this subgroup analysis, assuming 330 children per arm in the smallest subgroup (both low birthweight and underweight), which represents 10% loss-to-follow-up from 367 children, a standard deviation for WAZ of 1.1, and a correlation between baseline and 6-month WAZ in the subgroup of 0.1, we had approximately 80% power to detect a difference in WAZ of 0.24 SD at 6 months in infants receiving azithromycin compared to placebo. Power was low for mortality subgroup analyses. Assuming 330 children per arm and the observed 1.4% mortality prevalence by 6 months in the placebo arm in the smallest subgroup, the analysis would have 5% power to detect a 20% relative reduction and approximately 49% power to detect a reduction to 0.1% mortality prevalence in the azithromycin group (a 93% relative reduction).

### Statistical methods

We compared outcomes across subgroups aggregated by arm using logistic regression models for dichotomous outcomes (mortality, underweight, stunting, and wasting) and linear regression models for continuous outcomes (MUAC, weight gain, and length change). These models were adjusted for the child’s age at enrollment and sex and contained an indicator variable for each subgroup. To assess the effect of azithromycin versus placebo across the four subgroups, we conducted a logistic regression model for each dichotomous outcome or linear regression model for each continuous outcome with an interaction term for subgroup by randomized treatment group for estimation of interaction and the main effect of the subgroup. The MUAC analysis additionally included a term for baseline MUAC, but other analyses were unadjusted for baseline measures. P<0.05 was considered statistically significant for the main subgroup analyses as they were prespecified (**S2 File**).

We conducted a series of non-prespecified subgroup analyses to evaluate whether there were any differential effects of azithromycin in subgroups of neonates defined by other anthropometric deficits: 1) MUAC < 11.0, 2) MUAC < 11.0 or WAZ < -2, 3) WAZ < -3, 4) WLZ < -2, 5) WLZ < -3, and 6) any anthropometric deficit (low birthweight or WAZ < -2 or MUAC < 11.0 or WLZ < -2). Each of the 6 non-prespecified subgroups was analyzed separately. We evaluated the effect of azithromycin in each subgroup on all outcomes as in the primary subgroup analysis, using identical methods. Due to the number of comparisons (42 interaction *P*-values estimated) in the non-prespecified subgroups analyses, *P*<0.001 was considered statistically significant.

All analyses were two-sided. All analyses were conducted in R (The R Foundation for Statistical Computing, Vienna, Austria).

### Role of the funding source

The study was supported by the Bill and Melinda Gates Foundation (OPP1187628) and azithromycin and matching placebo were donated by Pfizer, Inc (New York, NY). The funders played no role in the design or conduct of the study, interpretation of data, or the decision to publish.

## Results

**Fig 1:**
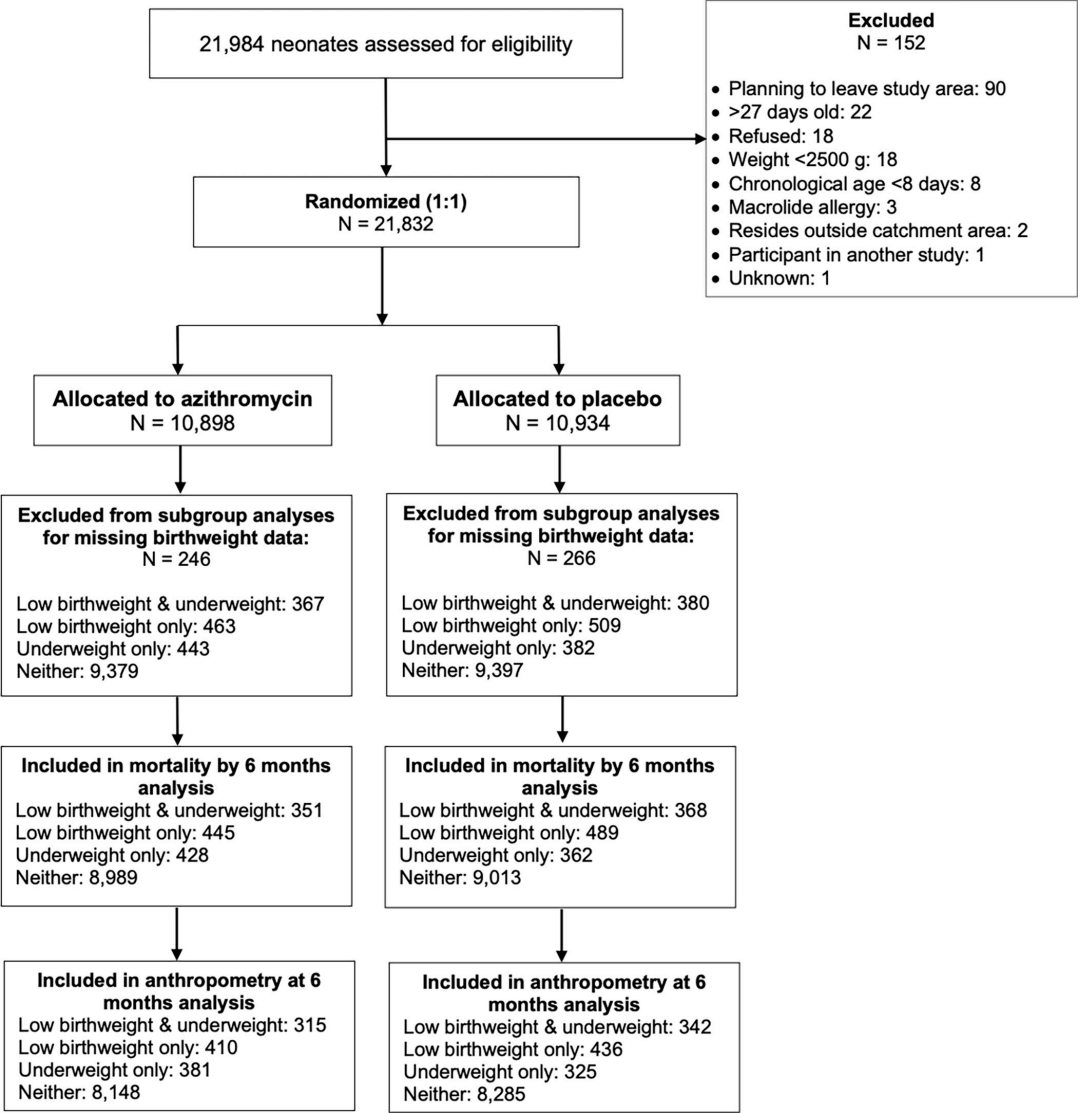
Recruitment, enrollment, and follow-up by treatment group and subgroup.

Of 21,832 neonates enrolled in the trial between April 2019 and December 2020, 21,320 (98%) had valid birthweight measurements in their *carnet de santé* and were eligible for inclusion in this analysis (**Fig 1**). Of these, 18,776 (88%) were neither low birthweight nor underweight at enrollment, 972 were low birthweight-only (5%), 825 (4%) were underweight-only, and 747 (3%) were both low birthweight and underweight at enrollment. Of the 21,320 enrolled infants eligible for this subgroup analysis, 20,445 (96%) were included in mortality analyses and 18,642 (87%) were included in anthropometric analyses. The percentage of infants lost to follow-up did not vary by subgroup or by treatment arm (**Fig 1**).

Although baseline characteristics were well balanced by treatment group across the subgroups (**Table 1**), as expected they differed by subgroup. Low birthweight-only infants were more often female (68% female) whereas underweight-only infants were more often male (33% female). Whereas infants in most subgroups had higher enrollment weights on average than birthweights, infants in the underweight-only subgroup were approximately the same weight at enrollment as they were at birth.

**Table 1:** Baseline characteristics by randomized treatment group among both low birthweight and underweight, low birthweight only, underweight only, and neither low birthweight nor underweight infants.

	Low birthweight and underweight		Low birthweight only		Underweight only		Not low birthweight or underweight	
	Azithromycin (N = 367)	Placebo (N = 380)	Azithromycin (N = 463)	Placebo (N = 509)	Azithromycin (N = 443)	Placebo (N = 382)	Azithromycin (N = 9379)	Placebo (N = 9397)
Chronological age, days								
Mean (SD)	17 (6)	17 (6)	13 (5)	13 (5)	16 (6)	15 (6)	12 (5)	12 (5)
Sex								
Female	181 (49%)	178 (47%)	318 (69%)	341 (67%)	149 (34%)	121 (32%)	4636 (49%)	4663 (50%)
Male	186 (51%)	202 (53%)	145 (31%)	168 (33%)	294 (66%)	261 (68%)	4743 (51%)	4734 (50%)
Mother’s age, years								
Mean (SD)	25 (6)	25 (7)	25 (6)	24 (6)	25 (7)	25 (7)	26 (6)	26 (6)
Birthweight, g								
Mean (SD)	2229 (166)	2226 (180)	2307 (125)	2304 (129)	2720 (252)	2682 (202)	3074 (371)	3081 (378)
Weight at enrollment, g								
Mean (SD)	2622 (137)	2621 (138)	2944 (342)	2958 (355)	2655 (147)	2645 (147)	3400 (438)	3401 (439)
Length at enrollment, cm								
Mean (SD)	48.3 (1.9)	48.2 (1.7)	49.2 (2.0)	49.4 (2.0)	49.1 (1.8)	49.1 (1.8)	50.8 (1.9)	50.9 (1.9)
WAZ								
Mean (SD)	-2.5 (0.4)	-2.5 (0.4)	-1.4 (0.6)	-1.4 (0.6)	-2.4 (0.3)	-2.4 (0.3)	-0.4 (0.8)	-0.4 (0.8)
WLZ								
Mean (SD)	-1.7 (1.1)	-1.6 (1.1)	-1.0 (1.2)	-1.1 (1.4)	-2.1 (1.2)	-2.1 (1.2)	-0.5 (1.3)	-0.5 (1.2)
LAZ								
Mean (SD)	-2.1 (1.0)	-2.2 (0.9)	-1.2 (1.0)	-1.1 (1.0)	-1.6 (0.9)	-1.6 (1.0)	-0.4 (1.0)	-0.4 (1.0)

Abbreviations: SD, standard deviation; WAZ, weight-for-age Z-score; WLZ, weight-for-length Z-score; LAZ, length-for-age Z-score

All-cause mortality by 6 months of age was highest among infants who were underweight at enrollment or both low birthweight and underweight (underweight: 1.5%; low birthweight and underweight: 1.3%; **Table 2**). In general, infants who were both low birthweight and underweight at enrollment had worse anthropometric outcomes and were more likely to be underweight, stunted, and wasted at 6 months of age compared to other infants (**Table 2**).

**Table 2:** Mortality and growth outcomes by subgroups defined by low birthweight (<2500 g) and/or underweight (weight-for-age Z-score < -2) at enrollment.

Outcome	N in Subgroup	N with Outcome (%) or Mean (SD)	Odds Ratio or Mean Difference (95% CI)*
**Mortality**			
Not LBW or UW	18,002	61 (0.3%)	1.00
LBW only	934	7 (0.7%)	2.32 (0.96 to 4.77)
UW only	790	12 (1.5%)	4.68 (2.35 to 8.59)
LBW and UW	719	9 (1.3%)	4.04 (1.82 to 8.02)
**Weight gain (g/day)**			
Not LBW or UW	16,433	23.1 (5.3)	Ref
LBW only	846	23.2 (5.1)	0.5 (0.1 to 0.8)
UW only	706	25.8 (5.5)	2.8 (2.4 to 3.2)
LBW and UW	657	24.8 (5.5)	2.2 (1.8 to 2.7)
**Length change (mm/day)**			
Not LBW or UW	16,433	0.88 (0.16)	Ref
LBW only	846	0.89 (0.17)	0.02 (0.009 to 0.03)
UW only	706	0.95 (0.17)	0.07 (0.06 to 0.08)
LBW and UW	657	0.95 (0.18)	0.09 (0.07 to 0.10)
**MUAC (cm)**			
Not LBW or UW	16,433	14.1 (1.1)	Ref
LBW only	846	13.8 (1.1)	-0.2 (-0.3 to -0.2)
UW only	706	13.7 (1.1)	-0.4 (-0.5 to -0.3)
LBW and UW	657	13.6 (1.1)	-0.5 (-0.6 to -0.4)
**Underweight (WAZ <-2)**			
Not LBW or UW	16,433	947 (5.8%)	1.00
LBW only	846	78 (9.2%)	1.78 (1.38 to 2.25)
UW only	706	123 (17.4%)	3.03 (2.45 to 3.73)
LBW and UW	657	132 (20.1%)	3.77 (3.05 to 4.64)
**Stunted (LAZ <-2)**			
Not LBW or UW	16,433	1,301 (7.9%)	1.00
LBW only	846	120 (14.2%)	2.26 (1.83 to 2.76)
UW only	706	118 (16.7%)	2.09 (1.69 to 2.57)
LBW and UW	657	179 (27.3%)	4.50 (3.71 to 5.43)
**Wasted (WLZ <-2)**			
Not LBW or UW	16,433	871 (5.3%)	1.00
LBW only	846	56 (6.6%)	1.29 (0.96 to 1.69)
UW only	706	68 (9.6%)	1.84 (1.40 to 2.37)
LBW and UW	657	65 (9.9%)	1.92 (1.45 to 2.50)

Abbreviations: SD, standard deviation; CI, confidence interval; LBW, low birthweight; UW, underweight; MUAC, mid-upper arm circumference; WAZ, weight-for-age Z-score; LAZ, length-for-age Z-score; WLZ, weight-for-length Z-score; *Adjusted for age at enrollment and sex

There was no evidence of a difference in mortality among children receiving azithromycin versus placebo in any subgroups defined by baseline nutritional status (*P* for interaction = 0.86). Neonates receiving azithromycin in low birthweight and underweight subgroups had reduced odds of mortality compared to those receiving placebo, but the number of events was small and differences were not statistically significant (**S1 Table**).

**Fig 2:**
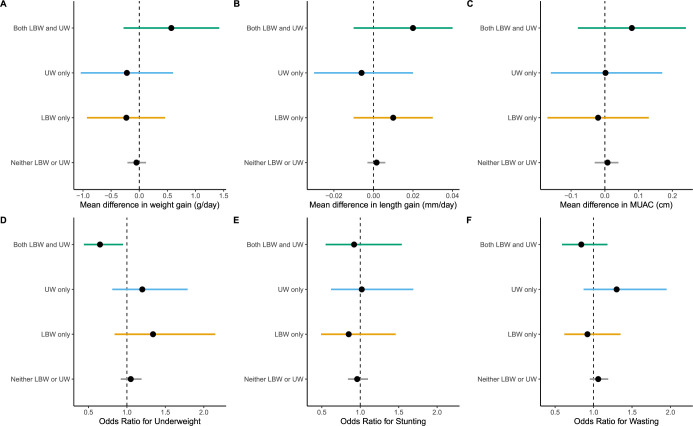
Mean differences and odds ratios in subgroups of neonates randomized to azithromycin versus placebo in subgroups defined by low birthweight (<2500 g) and/or underweight (weight-for-age Z-score < -2) at enrollment. Outcomes include weight gain (g/day; A), length gain (mm/day, B), mid-upper arm circumference (C), underweight (weight-for-age Z-score -2, D), stunted (length-for-age Z-score <-2), and wasted (weight-for-length Z-score <2) at 6 months.

Infants who were both low birthweight and underweight at enrollment who received azithromycin had greater weight gain in g/day than those who did not, but this difference was not statistically significant (mean difference 0.6 g/day, 95% confidence interval, CI, -0.3 to 1.4; **Fig 2**), and there was no evidence of a difference across subgroups (*P* for interaction = 0.46; **S1 Table**). Infants who were low birthweight and underweight who received azithromycin had reduced odds of being underweight at 6 months compared to placebo (odds ratio, OR, 0.65, 95% CI 0.44 to 0.95; **Fig 2**). However, there was no difference in odds of underweight among children who were low birthweight-only or underweight at enrollment-only who received azithromycin vs placebo and the interaction term for subgroup by treatment arm was not statistically significant (*P* for interaction = 0.06). There was no evidence of a difference between azithromycin and placebo in infants defined by low birthweight and/or underweight at enrollment subgroups for any other outcomes (**Fig 2; S1 Table**).

We found no significant evidence of a difference in infants randomized to azithromycin compared to placebo in a series of non-prespecified subgroups defined by anthropometric indicators other than low birthweight and underweight (**S2–S8 Tables, S1 and S2 Figs**). Infants who were severely underweight at enrollment (WAZ < -3) who received azithromycin had reduced odds of underweight (WAZ < -2) and stunting (LAZ < -2) at 6 months of age compared to severely underweight infants who received placebo, but these differences were not statistically significant when accounting for multiple comparisons (**S4 Table**).

## Discussion

Overall, we found no evidence of an effect of azithromycin on mortality or growth endpoints in low birthweight or underweight infants compared to placebo. There was some evidence that azithromycin reduced the risk of underweight by 6 months of age among infants who were both low birthweight and underweight compared to placebo. This subgroup of infants had persistent weight deficits, as they were born with a low birthweight and remained underweight by the time they were enrolled. These babies are likely at the highest risk of poor outcomes and thus may have been more likely to benefit from an antibiotic-based intervention than better-nourished babies. Previous randomized controlled trial evidence has suggested that antibiotics lead to weight gain in children with pre-existing morbidity, including malnutrition [21]. Amoxicillin is routinely used as part of outpatient treatment for severe acute malnutrition, and has been shown to lead to increased weight gain compared to placebo in randomized controlled trials [12, 13]. However, we found no evidence of a difference in weight outcomes in underweight-only infants receiving azithromycin compared to placebo. These babies had similar outcomes to the low birthweight and underweight babies and represent a subgroup of babies who had a normal birthweight but failed to gain sufficient weight during the first few weeks of life. These differences could be due to substantive differences in the low birthweight and underweight babies (e.g., weight deficits at birth that persist respond differently than new-onset underweight), or the finding among low birthweight and underweight babies could be due to chance.

The overall risk of mortality was low in this cohort. As the parent trial was designed both for efficacy and safety, and due to the potential risk of IHPS following azithromycin administration to neonates, enrolling centers had to be relatively close to major hospitals with pediatric surgical capacity. As a result, most enrollment sites were urban, which may have had lower mortality rates. Furthermore, for safety reasons due to possible increased risk of IHPS in small or preterm babies, babies had to be ≥2500 g to be eligible for enrollment in the parent trial. This selected for a cohort that, on average, was heavier than the general population and likely also had lower risk of mortality and poor outcomes. Low birthweight babies were eligible for inclusion if they gained enough weight to meet the 2500 g enrollment criterion by 27 days of age. As a result, the trial was underpowered for its mortality endpoint, and subgroup analyses for mortality were very underpowered. Overall, infants receiving azithromycin who were low birthweight and/or underweight had lower odds of mortality compared to those receiving placebo, a difference that was particularly pronounced for the underweight subgroup, but none of these differences were statistically significant. Previous studies have shown that WAZ is a strong predictor of undernutrition and mortality in both infants and older children [22–24], suggesting that mortality-reducing interventions may be most useful in these babies. However, further studies with larger sample sizes of low birthweight and/or underweight infants and in higher mortality populations are needed to fully elucidate the role of azithromycin for prevention of mortality in small infants. Improving growth in small infants may have benefits beyond reducing mortality. Malnutrition in infants has been linked to impaired cognitive development and increased risk of non-communicable disease [25, 26]. Longer-term evaluation of infants enrolled in antibiotic trials may yield important insights into any benefits on cognitive development and other endpoints potentially mediated by improved growth [27].

This analysis has several limitations. The population of infants in this trial is not representative of the general population nor of all low birthweight infants, and the study was not designed specifically to evaluate outcomes in low birthweight or small infants. To enter the trial, babies had to both be alive at 8 days and gain enough weight to meet the trial’s 2500 g enrollment criterion if they were born weighing <2500 g or lost significant weight during their first week after birth. While this does not bias by-arm comparisons, it may limit generalizability to generally healthier infants. Those born with a low birthweight and who are on a poor trajectory from birth may have different responses to azithromycin, and it is unclear if earlier treatment (e.g., during the first week after birth) would be beneficial for the highest risk infants. Measurement of gestational age was not available in the study facilities and not recorded in the *carnet de santé*; thus, we were unable to collect gestational age data. Low birthweight babies may have been small-for-gestational age, preterm, or both, and we were not able to distinguish between these causes. These babies may have different responses to azithromycin administration. Babies were enrolled before and during the COVID-19 pandemic. COVID-19 has had negative impacts on provision of neonatal and child health services for newborns, which could affect outcomes especially for smaller babies [28, 29]. Babies enrolled during the COVID-19 pandemic may not be generalizable to those enrolled outside of the pandemic. Breastfeeding status and duration could affect nutritional outcomes in infants. The vast majority (99.9%) of infants were breastfed at baseline, precluding subgroup analysis by breastfeeding status. Exclusive breastfeeding is recommended in Burkina Faso through 6 months of age. However, we did not collect data on duration of breastfeeding, and thus are unable to comment on whether the effect of azithromycin differs by breastfeeding status. We did not evaluate differences in subgroups of children by sex in subgroups defined by baseline anthropometric deficits, due to small subgroups and due to lack of statistically significant differences in the two-way interactions (e.g., anthropometric subgroups by treatment arm). There were no differences in outcomes in children receiving azithromycin compared to placebo in subgroups of children defined by sex for mortality [17] or anthropometric outcomes in the overall cohort. Finally, anthropometric outcomes were only measured at 6 months of age. It is possible that azithromycin could have had a shorter-term effect on growth endpoints but that there was no difference by 6 months of age.

Overall, we found no evidence of an effect of azithromycin in subgroups of infants defined by low birthweight and underweight, although azithromycin may help prevent underweight at 6 months in babies who had a low birthweight and were underweight at enrollment. Infants who were both low birthweight and underweight by the time they were enrolled in the trial may be at the highest risk of poor outcomes due to their persistent anthropometric deficits. Future trials designed specifically to evaluate the role of azithromycin for growth and mortality endpoints in low birthweight and underweight neonates and other small or nutritionally at-risk infants are needed to confirm this finding, and would need to consider selection for antimicrobial resistance, feasibility of implementation, and costs.

## Supporting information

S1 Checklist CONSORT checklist. (DOC)

S1 File Manual of operations and procedures for the NAITRE trial. (PDF)

S2 File Statistical analysis plan for the NAITRE trial. (PDF)

S1 Fig Venn diagram showing overlap in baseline anthropometric deficits defined by low birthweight (LBW; <2500 g), low weight-for-age Z-score (WAZ < -2), low weight-for-length Z-score (WLZ < -2), and low mid-upper arm circumference (MUAC < 11 cm). (DOCX)

S2 Fig Mean differences and odds ratios in subgroups of neonates randomized to azithromycin versus placebo in subgroups defined by low mid-upper arm circumference (< 11.0 cm), severe underweight (WAZ < -3), low MUAC or low WAZ (MUAC < 11.0 or WAZ < -2), low weight-for-length Z-score (WLZ < -2), severe wasting (WLZ < -3), and any anthropometric deficit (birthweight < 2500 g, WAZ < -2, WLZ < -2, or MUAC < 11.0 cm). Outcomes include weight gain (g/day; A), length gain (mm/day, B), mid-upper arm circumference (C), underweight (weight-for-age Z-score -2, D), stunted (length-for-age Z-score <-2), and wasted (weight-for-length Z-score <2) at 6 months.
(DOCX)

S1 Table Mortality and anthropometric endpoints by subgroup in infants receiving azithromycin versus placebo. (DOCX)

S2 Table Baseline demographic characteristics by non-prespecified subgroups defined by anthropometric deficits. (DOCX)

S3 Table Mortality and anthropometric endpoints by subgroup in infants defined by mid-upper arm circumference (MUAC; <11.0 cm or ≥11.0 cm) receiving azithromycin versus placebo. (DOCX)

S4 Table Mortality and anthropometric endpoints by subgroup in infants defined by severe underweight (WAZ < -3) or not severely underweight (WAZ ≥ -3) receiving azithromycin versus placebo. (DOCX)

S5 Table Mortality and anthropometric endpoints by subgroup in infants defined by low mid-upper arm circumference OR underweight (MUAC < 11.0 cm or WAZ < - 2) or neither low mid-upper arm circumference or underweight receiving azithromycin versus placebo. (DOCX)

S6 Table Mortality and anthropometric endpoints by subgroup in infants defined by low weight-for-length Z-score (WLZ < -2) or normal weight-for-length Z-score (WLZ ≥ - 2) receiving azithromycin versus placebo. (DOCX)

S7 Table Mortality and anthropometric endpoints by subgroup in infants defined by severe wasting (WLZ < -3) or WLZ ≥ -3 receiving azithromycin versus placebo. (DOCX)

S8 Table Mortality and anthropometric endpoints by subgroup in infants defined by any anthropometric deficit (low birthweight or WAZ < - 2 or WLZ < -2 or MUAC < 11) versus no anthropometric deficit at enrollment. (DOCX)
