# Supplementary material for: Infant mortality and growth failure after oral azithromycin among low birthweight and underweight neonates: A subgroup analysis of a randomized controlled trial
Source: PLOS Glob Public Health. 2023 May 15;3(5):e0001009. doi: 10.1371/journal.pgph.0001009 (PMC10184901; doi:10.1371/journal.pgph.0001009)
Supplement: S1 File — (PDF) [file pgph.0001009.s002.pdf]

Neonates and Azithromycin, an Innovation in the Treatment of Children in Burkina Faso NAITRE

Manual of Operations and Procedures

Centre de Recherche en Santé de Nouna

The Francis I. Proctor Foundation, Global Health Sciences
University of California, San Francisco

Catherine Oldenburg, ScD MPH
Ali Sié, MD PhD
Thomas Lietman, MD
Mamadou Bountogo, MD
Boubacar Coulibaly, PhD
Cheikh Bagagnan, MS
Eric Nebie, MD
Guillaume Compaoré, MD
Alphonse Zakane, MS
Mamadou Ouattara, MD
Valentin Boudo
Till Bärnighausen, MD ScD
Thuy D Doan, MD, PhD
Jeremy Keenan, MD MPH
Travis Porco, PhD MPH
Benjamin Arnold, PhD MPH
Kieran O'Brien, MPH
Elodie Lebas, RN
Jessica Brogdon, MPH
Catherine Cook, MPH
Ariana Austin, MS
Ying Lin, MSPH
William Godwin, MPH
Fanice Nyatigo, BS
Huiyu Hu, MS

Contents

Abbreviations 4
1. Chapter 1: Overview 5
 1.1 Executive summary 5
 1.2 Objectives 5
 1.3 Study Sites 5
2 Chapter 2: Context 6
3 Chapter 3: Study Design 7
 3.1 Recruitment 7
  3.1.1 Antenatal clinic recruitment 7
  3.1.2 Facility birth recruitment 8
  3.1.3 Key informant-based recruitment 8
 3.2 Enrollment 8
 3.3 Randomization 8
  3.3.1 Unit of randomization 8
  3.3.2 Randomizing treatment 8
4 Chapter 4: Eligibility 9
 4.1 Eligible Communities 9
 4.2 Eligible Participants 9
5 Chapter 5: Procedures 10
 5.1 Treatment 10
 5.2 Field worker safety assessment 10
 5.3 Vital status assessment 11
 5.4 Anthropometry assessment 11
6 Chapter 6: Adverse Event Monitoring and Safety Assessment 12
 6.1 Background on Infantile Hypertrophic Pyloric Stenosis in Neonates 12
 6.2 Screening for IHPS 13
7 Chapter 7: Training 14
8 Chapter 8: Study Medication 14
 8.1 Study Medication Description (from Pfizer, Inc.) 14
 8.2 Dosage Information 14
 8.3 Medication Procurement/Donation 14
 8.4 Medication Quality Control 14
 8.5 Study Treatment 15

8.6 Serious Adverse Events 15
8.7 Adverse Events Data 16
9 Chapter 9: Protection of Human Subjects 17
9.1 Institutional Review Board Approval 17
9.2 Informed Consent 17
10 Chapter 10: Data and Safety Monitoring Committee Charter 18
10.1 Primary Responsibilities of the DSMC 18
10.2 DSMC Membership 19
10.3 Conflicts of Interest 19
10.4 Timing and Purpose of the DSMC Meetings 19
10.5 Procedures to Ensure Confidentiality and Proper Communication 19
10.6 Statistical Monitoring Guidelines 21
11 Chapter 11: Data Collection, Management, and Security 22
11.1 Scope of Data 22
11.2 Data Storage, Management, and Security 22
11.3 Data Monitoring and Cleaning 22

Abbreviations

CRSN: Centre de Recherche en Santé de Nouna
DCC: Data Coordinating Center
DSMC: Data and Safety Monitoring Committee
GPS: global positioning system
HDSS: Health and Demographic Surveillance Site
IHPS: Infantile Hypertrophic Pyloric Stenosis
IRB: Institutional Review Board
MUAC: mid-upper arm circumference
NP swabs: nasopharyngeal swabs
PCR: polymerase chain reaction
STGG: skim milk tryptone glucose glycerin media
UCSF: University of California San Francisco
WHO: World Health Organization

1. Chapter 1: Overview

1.1 Executive summary

Although under-5 mortality rates are declining globally, neonatal mortality remains persistently high in many regions of sub-Saharan Africa.[1] Mass azithromycin distribution to children aged 1-59 months has been shown to reduce childhood mortality in Niger, Tanzania, and Malawi.[2] This study did not evaluate the effect of azithromycin administered during the neonatal period. Observational evidence from high income countries has suggested that macrolides, including erythromycin and azithromycin, may be associated with increased risk of development of infantile hypertrophic pyloric stenosis (IHPS).[3,4] However, these studies are limited by confounding by indication, as infants only receive antibiotics when they are ill.

We proposed an individually randomized trial of azithromycin versus placebo to establish the efficacy and safety of administration of a dose of azithromycin during the neonatal period. Our long-term goal is to generate evidence that can be used by neonatal and child survival programs related to the use of azithromycin in the youngest children who have the highest risk of mortality.

1.2 Objectives

1: Establish the safety and efficacy of a single dose of azithromycin among neonates. Neonates aged 8 to 27 days will be randomized to a single dose of azithromycin or placebo. *We hypothesize that neonates randomized to a single dose of azithromycin will have significantly lower all-cause mortality by 6 months of age, compared to those randomized to placebo.*

1.3 Study Sites

This study will be conducted in several regions of Burkina Faso, including peri-urban areas of Ouagadougou and Nouna town, and rural areas that are within 4 hours drive of a pediatric facility with capacity for performing pyloromyotomy.

2 Chapter 2: Context

Child mortality in West Africa is among the highest in the world. Although child health and mortality are improving worldwide, children in the Sahel and sub-Sahel regions of West Africa have the greatest risks of mortality.[1,5] Burkina Faso's current under-5 mortality rate is estimated 110 per 1,000 live births[5]. Similar to other countries in the region, the major causes of child mortality in Burkina Faso are malaria, respiratory tract infection, and diarrhea. Malnutrition acts as a major underlying contributor to mortality.[6,7] Neonatal mortality remains persistently high, with approximately 1/5th of neonatal mortality due to pneumonia, meningitis, and sepsis.[8] Interventions that address these underlying causes may be particularly efficacious for reducing mortality.

Younger children are at a higher risk of mortality. Approximately 2/3rd of under-5 deaths occur during the first year of life.[5] In general, the child mortality rate decreases as age increases. While some improvement has been observed, neonatal mortality is declining at a slower rate than post-neonatal childhood mortality.[5] Many child health interventions are designed specifically for children over 6 months of age, such as vitamin A supplementation, seasonal malaria chemoprevention, and lipid-based nutritional supplementation. Identification of strategies that are safe and effective for the youngest children will be required to address persistently high rates of neonatal and infant mortality.

The MORDOR I study demonstrated a significant reduction in all-cause child mortality following biannual mass azithromycin distribution. Across three diverse geographic locations in sub-Saharan Africa (Malawi, Niger, and Tanzania), biannual mass azithromycin distribution over a two-year period led to a 14% decrease in all-cause child mortality. In Niger, 1 in 5-6 deaths were averted. These results are qualitatively similar to those of a previous study of mass azithromycin distribution for trachoma control in Ethiopia, which found reduced odds of all-cause mortality in children in communities receiving mass azithromycin compared to control communities.[9]

In MORDOR I, the strongest effect of azithromycin was in the youngest cohort of children. Across all three countries, the strongest effect of azithromycin was consistently in children 1-5 months of age, with an approximately 25% reduction in all-cause mortality. However, MORDOR I was not optimized to target the youngest age groups. Although children as young as 1 month were eligible, biannual distributions might not reach some children until 7 months of age. On average, children were first treated at 4 months. Given that there may be a substantial benefit to treating children at younger ages, azithromycin strategies that are designed to target younger age groups may be even more beneficial for reducing child mortality.

Here, we propose a randomized controlled trial designed to evaluate the efficacy of a dose of azithromycin administered during the neonatal period for prevention of mortality within in the first year of life. We propose to randomize births in several geographic regions of Burkina Faso to a single dose of azithromycin or placebo between day 8 and 27 of life. This study is designed to provide evidence of the efficacy of azithromycin treatment for the youngest children.

3 Chapter 3: Study Design

We will enroll and follow children from birth until 6 months of age. Figure 1 shows the trial profile.

Figure 1. Trial profile

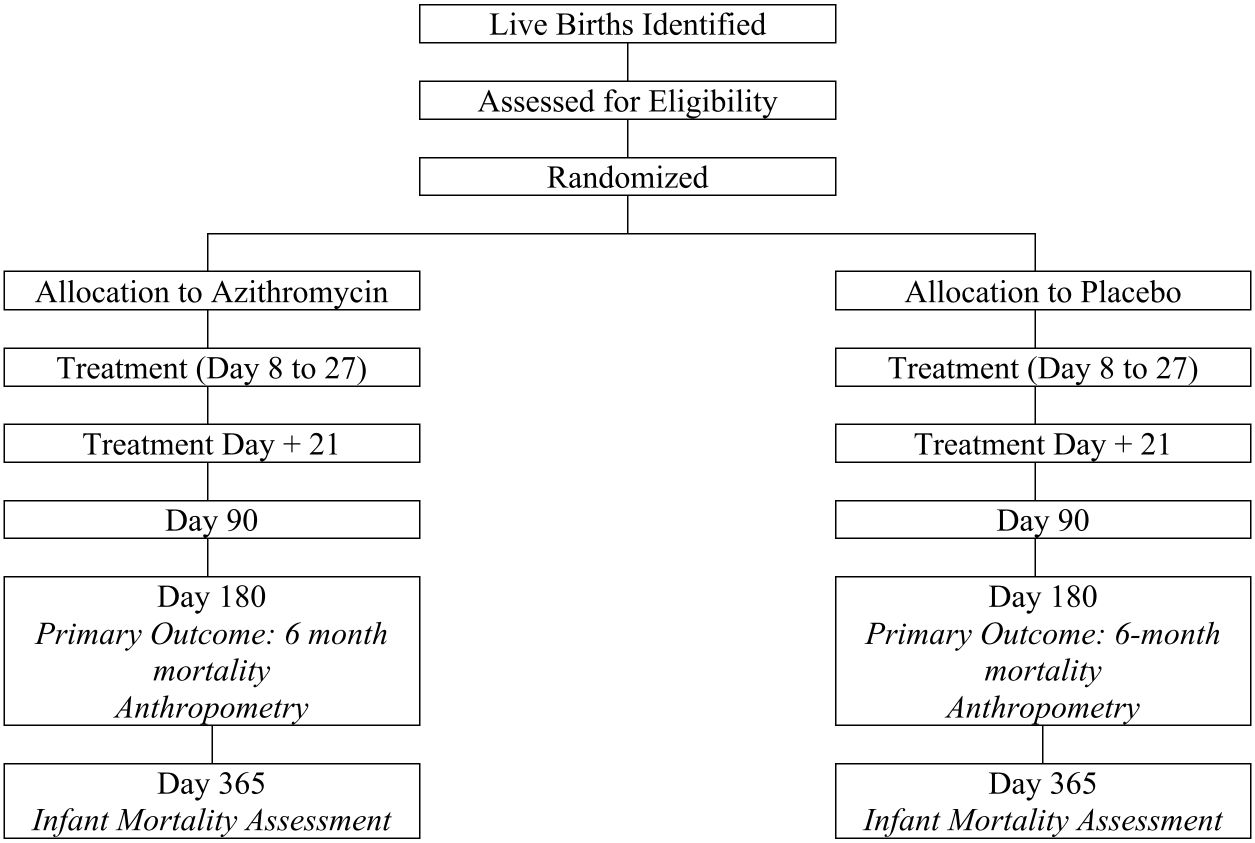

3.1 <u>Recruitment</u>

Mothers and infants will be recruited either during antenatal care visits, via facility births, or via a key informant who notifies study staff of a birth.

3.1.1 Antenatal clinic recruitment

Women in the third trimester of their pregnancy who attend antenatal care services in health facilities in the geographic catchment areas of the study will be approached for possible inclusion in the study. Study staff members will approach women in late pregnancy and explain and discuss the study with the woman. If the woman is interested in participating, her contact information and approximate due date will be recorded by the study staff member. For women who are recruited

during pregnancy, we will obtain verbal consent from the woman to follow the pregnancy until delivery. Once the baby is born we will obtain individual written consent to enroll the infant in the study. Formal eligibility assessment and enrollment will occur after birth.

3.1.2 Facility birth recruitment

Newborns and mothers will be recruited in facilities that have delivery services. Postpartum women will be approached by study staff, who will explain the study to the woman and assess the newborn's eligibility for the study. Newborns will be enrolled on the day of recruitment if they meet eligibility criteria (Chapter 4).

3.1.3 Key informant-based recruitment

In some rural areas and in areas with Health and Demographic Surveillance Sites (HDSS) we will employ key informants who will alert study staff of new births in their catchment area. Key informants will receive a small remuneration for reporting births.

3.2 Enrollment

Enrollment will occur the same day the child will be treated.
For each child assessed, a study staff member will review the eligibility criteria and fill out an eligibility assessment form (Appendix 2). If a child meets all of the eligibility criteria, a written informed consent process will be undertaken with the child's caregiver (Chapter 9. After eligibility assessment and consent, the child will be formally enrolled in the trial, a study identification number will be assigned to the child, and the child will be randomized a treatment arm.

3.3 Randomization

3.3.1 Unit of randomization

The unit of randomization is the neonate. Children will be randomized to azithromycin or placebo.

3.3.2 Randomizing treatment

Children will be randomized in a 1:1 fashion to a single dose of azithromycin or placebo. Randomization will occur via the tablet based on the child's identification number. Children will be assigned a random letter that corresponds to the treatment bottle. To prevent accidental unmasking, a total of 8 letters will be used in the study, with 4 referring to azithromycin and 4 to placebo. Each facility will be stocked with medication bottles labeled with each of the 8 letters.

4 Chapter 4: Eligibility

4.1 Eligible Communities

To be eligible for the trial, a community must meet the following criteria:

1. Within 4 hours of a facility that can provide services for pyloromyotomy (Ouagadougou or Bobo Dioulasso)
2. Accessible during the rainy season
3. Ultrasound machine available OR a facility in which an ultrasound machine could be placed is within 2 hours

4.2 Eligible Participants

Eligible participants are neonates who are screened within the first week of life. Specific inclusion and exclusion criteria are described below.

Inclusion Criteria (all must be met):

- Weight ≥2500 g
- Able to feed orally
- Family intends to stay in study area for at least 6 months
- Appropriate consent from at least one caregiver
- No known allergy to azalides
- Not living within one of the communities included in the community study (CHAT/CHATON)
- No hepatic failure manifested by neonatal jaundice

Exclusion Criteria (any excludes):

- Weight <2500 g
- Unable to feed orally
- Family planning to move
- Mother/caregiver not willing to participate
- Allergic to azalides
- Living in one of the communities included in the community study (CHAT/CHATON)
- Hepatic failure manifested by neonatal jaundice

Only children who meet all inclusion criteria and do not meet any exclusion criteria will be enrolled in the trial.

5 Chapter 5: Procedures

A general overview of study procedures is shown in Table 1. Eligibility assessment, enrollment, and randomization are covered in detail in Chapters 3 and 4. Appendix 2 contains all study forms.

Table 1. Overview of study procedures

**Day (since birth)**	**Activity**	**Study Form**
0 - 7	• Birth<br>• Sensitization	
8 to 27	• Assessment of eligibility<br>• Enrollment<br>• Randomization<br>• Anthropometry (weight, Length, MUAC)<br>• Treatment	Form 1: Eligibility assessment<br>Form 2: Baseline<br>Form 3: Treatment
Treatment Day + 21	• Field worker safety assessment including vital status	Form 4: Vital status<br>Form 5: Neonate AE and IHPS risk
90	• Field worker safety assessment including vital status	Form 4: Vital status<br>Form 5: Neonate AE and IHPS risk
180 (*primary outcome*)	• 6 months vital status assessment<br>• Anthropometry	Form 6: 6 months vital status Form<br>Form 5: Neonate AE and IHPS risk
365	• Vital status assessment	Form 4: vital status

5.1 <u>Treatment</u>

Details on study medication are provided in Chapter 8. No child will be treated before Day 8 or after Day 27. After treatment, the study staff member will record the treatment or if the child did not receive the treatment, and for those who were not treated, the reason the child did not receive the treatment.

5.2 <u>Field worker safety assessment</u>

Field workers will conduct an initial home visit 21 days after treatment, followed by a home visit at 3 and 6 months after birth. At 21 days following treatment, field workers will conduct a survey of adverse events, including if the child had any of the following symptoms:

- Fever
- Diarrhea

- Vomiting
- Abdominal pain
- Skin rash
- Constipation

The field worker will assess whether the caregiver had sought care for the child since the last time they spoke to the study team, and if so, what the reason was for the health care visit and if the child was hospitalized.

All caregivers who report that the child has been vomiting since the last study visit will be asked additional follow-up questions to screen for IHPS, including questions related to progression of vomiting, if the vomiting is projectile, and if the child is not gaining or losing weight. Those who respond affirmatively to those questions will be referred to the study pediatrician for additional follow-up and possible referral to a tertiary facility. (Appendix 3)

All caregivers will be given a pamphlet explaining the signs of IHPS. We will also include contact information and instructions on what to do if the infant develops symptoms. (Appendix 4).

5.3 Vital status assessment

A vital status assessment will occur at each follow-up study assessment. Field workers will record on the tablet if the child is alive, died, moved, or unknown.

5.4 Anthropometry assessment

We will record every enrolled child's weight and length at baseline and at 6 months of age. The anthropometry measurements will be taken at baseline when the neonate is enrolled and during the in person follow-up visit on day 180. Weight will be measured using a digital scale. Length will be taken using a Shorrboard to the nearest cm. We will also record the middle upper arm circumference of every enrolled child. (See appendix: anthropometric measurements protocol).

5.5 Passive Surveillance

In the Centre de Santé et de Promotion Sociale (CSPS, community health facility), we will conduct morbidity passive surveillance. Each CSPS will be equipped with a tablet for electronic capture of health facility visits. Each visit will be recorded, including the reason for the visit (e.g., fever, diarrhea, malnutrition, etc), the village of residenc, the person's age and sex, diagnosis (e.g., malaria, pneumonia, etc), treatment (e.g., antibiotic, antimalarial, etc), and timing of the visit (e.g., first versus follow-up visit). Note that this data is already routinely collected on paper forms. Identifying information like names will not be collected.

6 Chapter 6: Adverse Event Monitoring and Safety Assessment

There is some concern that azithromycin administered during the neonatal period will increase the risk of infantile hypertrophic pyloric stenosis (IHPS). Follow-up assessments have been designed to carefully monitor for IHPS following administration of study treatment.

6.1 Background on Infantile Hypertrophic Pyloric Stenosis in Neonates

IHPS is a condition in which the pylorus of the stomach becomes thickened, resulting in gastric outlet obstruction.[10] In developed countries, the incidence of IHPS is approximately 2 per 1,000 infants in the general population. While surgery is generally curative, the condition is lethal in the absence of surgery. The cause of IHPS is unknown and likely complex, and both genetic[11,12] and environmental[13,14] factors are thought to contribute to its development. Male neonates are disproportionately affected by IHPS, with a 4-5:1 male to female ratio.[10,12,15] Other risk factors for IHPS include prone sleeping position, bottle/formula feeding,[14,16,17] preterm birth,[13,14] cesarean section delivery,[13,14] and birth order.[10,13-15] IHPS incidence appears to be decreasing over time, which some have attributed to public health interventions promoting supine sleeping position and exclusive breastfeeding.[18,19]

In addition to genetic and environmental factors, erythromycin is associated with increased risk of infantile hypertrophic pyloric stenosis (IHPS).[20] Given that azithromycin is a related compound, there is some concern that azithromycin may lead to increased risk of IHPS in neonates. Limited evidence exists of the risk of IHPS among neonates treated with azithromycin.[21] Two randomized controlled trials have assessed the use of intravenous azithromycin for prevention of bronchopulmonary dysplasia (BPD) in low birthweight infants (<1,250g) compared to placebo within 72 hours of birth.[22,23] Of 263 neonates enrolled in the two studies (N=130 receiving IV azithromycin), no cases of IHPS were reported. A third non-placebo controlled randomized trial of azithromycin prophylaxis for BPD in premature neonates additionally reported no cases of IHPS among 53 neonates receiving azithromycin.[24] An observational study of 58 infants receiving azithromycin following exposure to a healthcare worker with pertussis did not identify any cases of IHPS.[25] The largest study of azithromycin exposure in neonates is a retrospective cohort of more than one million infants in the TRICARE Management Activity military health system, which reported an overall IHPS rate of 2.3 per 1,000 (95% CI 2.2 to 2.4) among children in the first 90 days of life.[4] Of 4,875 infants prescribed azithromycin, there were 8 cases IHPS, 3 of which occurred when azithromycin was prescribed during the first 14 days of life and 5 of which during the first 15-42 days of life. Overall, there was no significant difference in IHPS rate in infants treated with azithromycin versus IHPS rates among infants who had not received an antibiotic during the first 90 days of life were not presented by time since birth. Table 2 lists the rates of IHPS following azithromycin, erythromycin, or cephalexin prescription.

Table 3. Unadjusted rate of IHPS in infants with oral azithromycin, per 1,000

	**Azithromycin**	**Erythromycin**	**Cephalexin**
0-14 days[1]	20.3 (4.2-59.2)	30.9 (14.1-58.7)	2.2 (0.05-
15-42 days[1]	6.9 (2.2-16.1)	9.3 (3.0-21.8)	12.2)
43-90 days[1]	0 (0-0.9)	2.8 (0.6-8.1)	3.1 (1.0-7.2)
Overall	1.6 (0.7-3.2)	8.9 (5.2-14.3)	0 (0-1.6)

			1.4 (0.5-3.0)

[1]Day of azithromycin prescription, from birth

There is relatively little evidence of the epidemiology of IHPS in sub-Saharan Africa. A study in Nigeria documented 57 cases of IHPS from 1978-2008 at a university teaching hospital, with only a single case from 2003-2008.[19] A study of 102 cases of IHPS over a 5-year period in Tanzania documented a 4.9% mortality rate despite surgical intervention.[26] The risk of mortality was higher in infants under 2 weeks of age and those with delayed presentation to care. A retrospective study at a tertiary hospital in Ethiopia found that 12.9 per 1,000 admissions were due to IHPS, with a 3.3% mortality rate.[15] In Ouagadougou, an unpublished case series of 32 infants treated for IHPS showed a 6.3% mortality rate, which was attributed to late presentation due to the infant traveling from far outside the city.

6.2 Screening for IHPS

Field workers will conduct home visits with caregivers 21 days following treatment, as described in Chapter 5. Any child suspected of having IHPS will be immediately referred to the study pediatrician for evaluation. Evaluation will include ultrasonography and physical exam. Physical exam will include assessment of the pyloric olive (a thickened and elongated pylorus). Any child with a positive physical exam for a pyloric olive will be immediately transferred to the pediatric surgical unit in Ouagadougou or Bobo.

Images will be taken including the longitudinal pylorus with canal length measurement, transverse pylorus with muscle thickness measurement, and the relationship of the pylorus to the gallbladder. IHPS will be strongly suspected in infants with a permanently closed pylorus and exaggerated, retrograde gastric peristalsis. Diagnostic measurements include pyloric muscle thickness (diameter of a single muscular wall on a transverse image) >4 mm, length (longitudinal measurement) >15 mm, and pyloric volume >1.5 cc.[27] The child should be placed with their right side down, and the pylorus watched to determine if it opens. Small infants not below the pathologic limits with a permanently closed pylorus will also be considered for further workup.[28] Any child with a pyloric muscle thickness >4 mm will be immediately transferred to the pediatric surgical unit in Ouagadougou. In addition any child with normal measurements, but no food is passing, will be transferred.

All children will be assessed for electrolyte disturbance and dehydration, and rehydration and correction of electrolyte disturbance will occur prior to surgery.

Any children receiving surgery for IHPS will be followed-up with 1 week after the procedure. The follow-up will be a phone call or at-home visit. The child's vital status information will be collected. Another follow-up visit will take place 4 weeks after the procedure.

7 Chapter 7: Training

CRSN and UCSF will work together prior to the start of the study to standardize all study procedures. We will review the format, general logistics, and procedures for the recruitment, enrollment, randomization and anthropometric measurements. We will review all study protocols, including informed consent procedures and documentation and adverse event monitoring. The importance of screening for IHPS and safety protocols will be stressed. Refresher trainings will occur as needed and supervisors and study investigators will routinely review data and procedures to ensure fidelity to protocol.

8 Chapter 8: Study Medication

Neonates enrolled in the study will be offered weight-based, directly observed, oral suspension azithromycin or placebo. We will monitor adverse events following treatment as described in Chapter 6.

8.1 Study Medication Description (from Pfizer, Inc.)

Azithromycin

Zithromax® for oral suspension is supplied in bottles containing azithromycin dehydrate powder equivalent to 1200mg per bottle and the following inactive ingredients: sucrose; tribasic anhydrous sodium phosphate; hydroxypropyl cellulose; xanthan gum; FD&C Red #40; and flavoring including spray dried artificial cherry, crème de vanilla, and banana. After constitution, a 5mL suspension contains 200mg of azithromycin.

8.2 Dosage Information

Azithromycin and placebo will be administered as a single dose, in oral suspension form for children. Dosing will follow the WHO recommendations for treatment of active trachoma:

- Single dose of 20mg/kg in children (up to the maximum adult dose of 1g)

Individuals who are allergic to macrolides/azalides will not be treated.

8.3 Medication Procurement/Donation

Azithromycin (Zithromax®) and the placebo have been donated by the Pfizer Corporation. There will be no costs to acquiring the study medication. Pfizer, Inc. will ship azithromycin and placebo directly to the study sites. Representatives of each study site will manage the customs process and transport the medication from the port to storage sites.

8.4 Medication Quality Control

Study medication will be shipped by Pfizer directly to CRSN and stored at CRSN research offices prior to distribution to each study site. The study coordinator and other staff will regularly check and record the study medication expiration dates. The expiration dates on the medication containers will be strictly monitored and all expired study medicine will be discarded appropriately. The study coordinator will work with each health facility to ensure that they have appropriate stock of all study medications.

8.5 Study Treatment

Study treatment procedures are detailed in Chapter 5.

8.6 Serious Adverse Events

Any serious adverse events (SAE) will be reported to Pfizer. An **IIR SAE Form** (*Investigator-Initiated Research Serious Adverse Events Form*) will be completed for each event. (See Appendix for form and complete instructions.)

According to Pfizer, an SAE is any adverse event that:

- Results in death
- Is life-threatening (i.e., causes an immediate risk of death)
- Requires inpatient hospitalization or prolongation of existing hospitalization
- Results in persistent or significant disability or incapacity
- Results in a congenital anomaly or birth defect

Or that is considered to be:

- An important medical event

All participants will be advised to alert a village health worker if they experience, within one week of treatment, a serious adverse event (by the preceding definition). An SAE report must be submitted for all deaths in the study – regardless of the time of treatment. The local health worker will report to the study coordinator; who must, within 24 hours, submit a Pfizer **IIR SAE Form** to mordor.burkina.sae@gmail.com. AS and TL will review, and forward to Pfizer and/or the Medical Monitor, as appropriate. SAEs must be submitted to Pfizer within 24 hours of receipt from the on-site coordinator. AS and TL will also forward SAE to DSMC if meets criteria of being possibly related to study drug. All deaths reported that are not serious adverse events resulting from the treatment will be reported to Pfizer on a quarterly basis.

One or more qualified investigators will be posted in the health centers at the recruitment site. Mothers of included children will be encouraged to visit these health centers in the event of an adverse event. Any adverse events occurring during the trial will be covered by the study free of charge. Participants with a serious event will be cared for in the nearest hospital. The research team will be in constant contact with pediatricians from regional and university hospitals and pediatric surgeons to manage potential serious adverse events. Surgical units will be involved in the diligent management of pyloric stenosis cases. All recruitment sites will be within a two-hour drive of a

Regional Hospital Centre where there is a paediatrician and a radiologist to enable rapid diagnosis of pyloric stenosis and within 4 hours of Bobo-Dioulasso or Ouagadougou to enable rapid management.

The reporting of any serious adverse event will follow national procedures in Burkina Faso.
- In the event of a non-serious adverse event, the CSPS will process the cases and the national reporting form will be completed.
- In the event of serious events, the CSPS will contact the study doctors on the same day. The patient will then be evacuated to an appropriate level of care for management.
- The declaration will be made to the National Agency for Pharmaceutical Regulation in accordance with the regulations and within the deadlines (7 days in the event of death or life-threatening prognosis, 15 days in other serious and unexpected cases, 15 days in new facts) at pharmacovigilance.burkina@sante.gov.bf
- The ethics committees will also be informed of the occurrence of this event within the same time frame.
- The sponsor will be informed within 24 hours in the event of a serious event.

Pfizer will be reported within 24 business hours from the date of knowledge of the serious adverse event

8.7 Adverse Events Data

We will keep records and report all adverse events of azithromycin to the DSMC. We will report both efficacy and side effects of azithromycin For any "sudden deaths" believed to be associated with azithromycin treatment, key informants will immediately notify the verbal autopsy interviewer via SMS message or another appropriate form of rapid communication.

9 Chapter 9: Protection of Human Subjects

Before the study begins, CRSN and UCSF will obtain formal ethical approval from their respective ethics committees as well as national ethical approval in Burkina Faso. In addition, local staff will approach community leaders to describe the study and answer any questions. Study staff will proceed only if local leadership consents to participate. At the individual level, we will obtain written consent from a parent or guardian for all study activities with patient contact, including following pregnancies for potential enrollment, treatment, and follow-up visits.

If, at any time, a parent or guardian elects to withdraw a family member from the study, they will be free to do so. Individuals who withdraw will be offered the same medical treatment outside the study.

9.1 Institutional Review Board Approval

UCSF Committee on Human Research
UCSF's Committee on Human Research will annually review study protocol for ethical approval.

CRSN Comité Institutionnel d'Ethique
The study protocol will be reviewed and granted ethical approval by the Comité Institutionnel d'Ethique at the CRSN headquarters before any patient-related research activities begin.

National Health Ethics Committee of Burkina Faso.
The study protocol will be reviewed and granted ethical approval by the National Health Ethics Committee of Burkina Faso before any patient-related research activities begin and annually.

9.2 Informed Consent

First, the chairman of each village will be asked for permission to include the village in the study. Additionally, the study will be discussed with all adults in the village by team members who speak the local language(s).

Informed consent scripts will be translated into local languages before the study can begin. Consent scripts will then be back-translated by a different party to ensure comprehension. Consent scripts will be submitted and approved by national IRB committees in Burkina Faso prior to study implementation. Then they will be read aloud to each study participant (and his/her parent/guardian) by a local team member who is a native speaker of the local language to ensure that they understand the risks and benefits of participating in all study activities. Young adults and children under 18 years of age, who cannot give consent by law, will be included in the study only following the receipt of verbal informed consent from a parent or guardian. If, at any time, a parent or guardian elects to withdraw themselves or a family member from the study it will be made clear that they can, without consequences.

10 Chapter 10: Data and Safety Monitoring Committee Charter

This Charter is for the Data Safety and Monitoring Committee (DSMC) for *Neonatal Azithromycin to Prevent Infant Mortality. (OPP 1187628)*

The Charter will define the primary responsibilities of the DSMC, its relationship with other trial components, its membership, and the purpose and timing of its meetings. The Charter will also provide the procedures for ensuring confidentiality and communication, statistical monitoring guidelines to be implemented by the DSMC, and an outline of the content of the Open and Closed Reports that will be provided to the DSMC.

10.1 Primary Responsibilities of the DSMC

The DSMC will be responsible for safeguarding the interests of trial participants, assessing the safety and efficacy of the interventions during the trial, and monitoring the overall conduct of the trial. The DSMC will provide recommendations about stopping or continuing the trial. To contribute to the integrity of the trial, the DSMC may also formulate recommendations relating to the selection/recruitment/retention of participants, to protocol-specified regimens, and the procedures for data management and quality control.

The DSMC will be advisory to the trial leadership group, hereafter referred to as the Steering Committee (SC). The SC will be responsible for promptly reviewing the DSMC recommendations and determining, whether to continue or terminate the trial, and to determine whether amendments to the protocol are required. If needed, the DSMC may seek the advice of a content expert outside of the committee.

10.2 DSMC Membership

The DSMC is an independent multidisciplinary group consisting of epidemiologists, biostatisticians, bioethicists, and clinicians that collectively has experience in pediatrics, the management of infectious diseases, and in the conduct and monitoring of randomized clinical trials including subsaharan Africa.

10.3 Conflicts of Interest

The DSMC membership has been restricted to individuals free of apparent conflicts of interest. The source of these conflicts may be financial, scientific, or regulatory. Thus, neither study investigators nor individuals employed by the sponsor, nor individuals who might have regulatory responsibilities for the trial products, are members of the DSMC.

The DSMC members will disclose to fellow members any consulting agreements or financial interests they have with the sponsor of the trial, with the contract research organizations (CRO) , or with other sponsors having products that are being evaluated or that are competitive with those in the trial. The DSMC will be responsible for deciding whether these consulting agreements or financial interests materially impact their objectivity.

The DSMC members will be responsible for advising fellow members of any changes in any of the membership requirements that occur during the course of the trial. It may be appropriate for DSMC members who develop significant conflicts of interest resign from the DSMC.

DSMC membership is to be for the full duration of the trial. If any members leave the DSMC, the SC, in consultation with the DSMC, will promptly appoint a replacement.

10.4 Timing and Purpose of the DSMC Meetings

Organizational Meeting

The initial meeting of the DSMC will be an Organizational Meeting. This is during the final stages of protocol development and the purpose is to provide advisory review of scientific and ethical issues relating to study design to discuss the standard operating procedures and to discuss the format and content of the Open and Closed Reports that will be used to present trial results.

The Organizational Meeting will be attended by all DSMC members, lead trial investigators, and the trial biostatistician. The DSMC will be given the drafts of the trial protocol, the Statistical Analysis Plan, the DSMC Charter, and the current version of the case report forms. At subsequent meetings, committee members will receive Open and Closed Data Reports.

Formal Interim Analysis Meetings

One or more 'Formal Interim Analysis' meetings will be held to review data relating to treatment safety and efficacy, and quality of trial conduct. There will be at least two interim decisions to be made by the DSMC, at approximately 12 months and 24 months into the study.

10.5 Procedures to Ensure Confidentiality and Proper Communication

To enhance the integrity and credibility of the trial, procedures will be implemented to ensure the DSMC has access to all emerging information from the trial regarding comparative results of efficacy and safety, aggregated by treatment arm.

Closed Sessions
Sessions involving only DSMC members and, where appropriate, those unmasked trial investigators (on the Data Coordinating Committee) who generate the Closed Reports (called Closed Sessions) will be held to allow discussion of confidential data from the trial, including information about the relative efficacy and safety of interventions.

At a final Closed Session, the DSMC will develop a consensus on its list of recommendations, including that relating to whether the trial should continue.

Open Session
In order for the DSMC to have access to information provided, by study investigators, or members of regulatory authorities, a joint session between these individuals and DSMC members will be held between the Closed Sessions.

Open and Closed Reports
For each DSMC meeting, Open and Closed Reports will be provided. Open Reports, will include data on recruitment and baseline characteristics, pooled data on eligibility violations, and completeness of follow-up and compliance. The study statistician (TCP) will prepare these Open Reports.

Closed reports, available only to those attending the Closed Sessions of the meeting, will include analyses of primary and secondary efficacy endpoints, including subgroup and adjusted analyses, AEs and symptom severity, , and Open Report analyses that are displayed by intervention group. These Closed Reports will be prepared by the study biostatistician.

The Open and Closed Reports should provide information that is accurate, with follow-up that is complete to within two months of the date of the DSMC meeting. The Reports should be provided to DSMC members approximately three days prior to the date of the meeting.

Minutes of the DSMC Meeting
The research team will prepare minutes for the open portion of the meeting, including the DSMC's recommendations.

Recommendations to the Steering Committee (SC)
At each meeting of the DSMC during the trial, the committee will make a recommendation to the Steering Committee to continue or terminate. This recommendation will be based primarily on safety and efficacy considerations and will be guided by statistical monitoring guidelines defined in this Charter.

Recommendations to amend the protocol or conduct of the study made by the DSMC will be considered and accepted or rejected by the SC. The SC will be responsible for deciding whether to continue or to stop the trial based on the DSMC recommendations.

The DSMC will be notified of all changes to the protocol or to study conduct. The DSMC concurrence will be sought on all substantive recommendations or changes to the protocol or study conduct prior to implementation.

The SC may communicate information in the Open Report to the sponsor and may inform them of the DSMC recommended alterations to study conduct or early trial termination in instances in which the SC has reached a final decision agreeing with the recommendation. The SC will maintain confidentiality of all information it receives other than that contained in the Open Reports until after the trial is completed or until a decision for early termination has been made.

10.6 Statistical Monitoring Guidelines

The SC will propose statistical rules for a futility stopping rule (requested by the sponsor) and an efficacy stopping rule at the first DSMC meeting. A decision will be made whether the efficacy stopping rule is appropriate for the study.

11 Chapter 11: Data Collection, Management, and Security

11.1 Scope of Data

Mortality and morbidity data will be collected in this trial. Data include treatment, vital status, adverse events, demographic and birth-related characteristics, and specimen collections.

Demographic and Birth Data

At baseline, trained field workers will collect information on the child's gestational age and birthweight and basic demographic information about the mother (age, education, etc).

Treatment Data

Trained field workers who administer treatment will record each treatment dose in the mobile application at the time of treatment of the child. Data will include the child's weight (used to calculate the dose), the dose, if the child received the treatment, and if not, why the child did not receive treatment.

Vital Status Data

Trained field workers will collect information on the vital status of each infant enrolled in the study at multiple pre-specified time points during the study. Vital status will include alive, died, moved, or unknown.

Adverse Events

Trained health workers will collect data on adverse events that arise during the study, including gastrointestinal symptoms, hospitalizations, and other healthcare visits.

11.2 Data Storage, Management, and Security

Data will be recorded electronically using handheld mobile devices with custom-made software applications and uploaded daily onto a secure, password protected, central server. Rapid transfer of electronically captured data will allow nearly real-time monitoring of activity at the study site. Each study site will have a local data coordinating center within the study area. All handheld devices and data entry coordinating centers will be password protected, and all changes in data will be noted, including the date of the change, and the person who made the change. To ensure the quality of the data, we will conduct training sessions before each biannual census where needed. The central database application will use hard disk encryption and physical protection of the server (which is to be maintained in a locked room accessible only to authorized personnel). The database will be based on mySQL (which supports standard SQL queries). Data will be backed up off site (providing integrity in case of the physical loss of the server). Data will never be deleted from mobile capture devices until at least one offsite backup has been completed. Data security during electronic transfer will be achieved through use of the Advanced Encryption Standard (AES).

11.3 Data Monitoring and Cleaning

Data monitoring and cleaning will be overseen by the data coordinating center (DCC) at the coordinating site. Data collection will be monitored on a weekly basis by the site study coordinator

using a dashboard function. The dashboard will consist of the following reports by study site: Date Household Census Completed, Number of Households Census Completed by Village, Percent Household Census Completed by village, Treatment Status by Worker, Age Distribution by Worker, Sex Distribution by Worker, GPS Missing by Worker, GPS Missing by Village, Number of Records Synced by Date, Assigned Treatment by Given Treatment, Treatment Status by Age, Treatment Status by Village, Age Distribution by Village, and Sex Distribution by Village.

The DCC will ensure that the site study coordinators log on to the dashboard at least weekly to confirm the status of the dashboard. In addition, upon each village census completion, the DCC will create and maintain a Stata program to identify data quality concerns. Any such concerns, which must be addressed at the site-specific level, will be queried by the DCC. At every phase, as each village is completed and the data is considered cleaned, the data will be locked and a list of deaths will be generated and provided to each site for verbal autopsy.

<u>Appendix</u>

Appendix 1. Revision History
Appendix 2. Study Forms
Appendix 3. Referral decision tree
Appendix 4. IHPS Pamphlet
Appendix 5. Anthropometry protocol

References

1 Golding N, Burstein R, Longbottom J, *et al.* Mapping under-5 and neonatal mortality in Africa, 2000–15: a baseline analysis for the Sustainable Development Goals. *The Lancet* 2017; : 1–12.

2 Keenan JD, Bailey RL, West SK, *et al.* Mass azithromycin distribution for reducing childhood mortality in sub-Saharan Africa. *N Engl J Med* 2018; **In press**.

3 Lund M, Pasternak B, Davidsen RB, *et al.* Use of macrolides in mother and child and risk of infantile hypertrophic pyloric stenosis: nationwide cohort study. *BMJ* 2014; **348**: g1908–8.

4 Eberly MD, Eide MB, Thompson JL, Nylund CM. Azithromycin in Early Infancy and Pyloric Stenosis. *Pediatrics* 2015; **135**: 483–8.

5 Wang H, Bhutta ZA, Coates MM, *et al.* Global, regional, national, and selected subnational levels of stillbirths, neonatal, infant, and under-5 mortality, 1980–2015: a systematic analysis for the Global Burden of Disease Study 2015. *The Lancet* 2016; **388**: 1725–74.

6 Muller O, Garenne M, Kouyate B, Becher H. The association between protein–energy malnutrition, malaria morbidity and all-cause mortality in West African children. *Tropical Medicine & International Health* 2003; **8**: 507–11.

7 Becher H, Muller O, Dambach P, *et al.* Decreasing child mortality, spatial clustering and decreasing disparity in North-Western Burkina Faso. *Tropical Medicine & International Health* 2016; **21**: 546–55.

8 World Health Organization,, UNICEF. WHO/UNICEF Joint Statement: Managing possible serious bacterial infection in young infants 0–59 days old when referral is not feasible. 2017.

9 Porco TC, Gebre T, Ayele B, *et al.* Effect of Mass Distribution of Azithromycin for Trachoma Control on Overall Mortality in Ethiopian Children: A Randomized Trial. *JAMA* 2009; **302**: 962–8.

10 Ranells JD, Carver JD, Kirby RS. Infantile Hypertrophic Pyloric Stenosis: Epidemiology, Genetics, and Clinical Update. *Advances in Pediatrics* 2011; **58**: 195–206.

11 Everett KV, Ataliotis P, Chioza BA, Shaw-Smith C, Chung EMK. A novel missense mutation in the transcription factor FOXF1 cosegregating with infantile hypertrophic pyloric stenosis in the extended pedigree linked to IHPS5 on chromosome 16q24. *Pediatr Res* 2016; **81**: 632–8.

12 Peeters B, Benninga MA, Hennekam RCM. Infantile hypertrophic pyloric stenosis —genetics and syndromes. *Nature Publishing Group* 2012; **9**: 646–60.

13 Svenningsson A, Svensson T, Akre O, Nordenskjöld A. Maternal and pregnancy characteristics and risk of infantile hypertrophic pyloric stenosis. *Journal of Pediatric Surgery* 2014; **49**: 1226–31.

14 Zhu J, Zhu T, Lin Z, Qu Y, Mu D. Perinatal risk factors for infantile hypertrophic pyloric stenosis: A meta-analysis. *Journal of Pediatric Surgery* 2017; **52**: 1389–97.

15 Tadesse A, Gadisa A. Infantile hypertrophic pyloric stenosis: A retrospective study from a tertiary hospital in Ethiopia. *East Cent Afr J Surg* 2014; **19**: 120–4.

16 Wayne C, Hung J-HC, Chan E, Sedgwick I, Bass J, Nasr A. Formula-feeding and hypertrophic pyloric stenosis: is there an association? A case–control study. *Journal of Pediatric Surgery* 2016; **51**: 779–82.

17 Krogh C, Biggar RJ, Fischer TK, Lindholm M, Wohlfahrt J, Melbye M. Bottle-feeding and the Risk of Pyloric Stenosis. *Pediatrics* 2012; **130**: e943–9.

18 Sommerfield T, Chalmers J, Youngson G, Heeley C, Fleming M, Thomson G. The changing epidemiology of infantile hypertrophic pyloric stenosis in Scotland. *Archives of Disease in Childhood* 2008; **93**: 1007–11.

19 Osifo DO, Evbuomwan I. Does Exclusive Breastfeeding Confer Protection Against Infantile Hypertrophic Pyloric Stenosis? A 30-year Experience in Benin City, Nigeria. *Journal of Tropical Pediatrics* 2008; **55**: 132–4.

20 Murchison L, Coppi P, Eaton S. Post-natal erythromycin exposure and risk of infantile hypertrophic pyloric stenosis: a systematic review and meta-analysis. *Pediatric Surgery International* 2016; **32**: 1147–52.

21 Smith C, Egunsola O, Choonara I, Kotecha S, Jacqz-Aigrain E, Sammons H. Use and safety of azithromycin in neonates: a systematic review. *BMJ Open* 2015; **5**: e008194–8.

22 Ballard HO, Anstead MI, Shook LA. Azithromycin in the extremely low birth weight infant for the prevention of Bronchopulmonary Dysplasia: a pilot study. *Respir Res* 2007; **8**: 1793–9.

23 Ballard HO, Shook LA, Bernard P, *et al.* Use of azithromycin for the prevention of bronchopulmonary dysplasia in preterm infants: a randomized, double-blind, placebo controlled trial. *Pediatr Pulmonol* 2010; **46**: 111–8.

24 Gharehbaghi MM, Peirovifar A, Ghojazadeh M, Mahallei M. Efficacy of azithromycin for prevention of bronchopulmonary dysplasia (BPD). *Turk J Med Sci* 2012; **42**: 1070–5.

25 Friedman DS, Robinette Curtis C, Schauer SL, *et al.* Surveillance for Transmission and antibiotic Adverse Events Among Neonates and Adults Exposed to a Healthcare Worker With Pertussis. *Infect Control Hosp Epidemiol* 2015; **25**: 967–73.

26 Chalya PL, Manyama M, Kayange NM, Mabula JB, Massenga A. Infantile hypertrophic pyloric stenosis at a tertiary care hospital in Tanzania: a surgical experience with 102 patients over a 5-year period. *BMC Research Notes* 2015; : 1–6.

27 Rohrschneider WK, Mittnacht H, Darge K, Tröger J. Pyloric muscle in asymptomatic infants: sonographic evaluation and discrimination from idiopathic hypertrophic pyloric stenosis. *Pediatric Radiology* 1998; **28**: 429–34.

28 Said M, Shaul DB, Fujimoto M, Radner G, Sydorak RM, Applebaum H. Ultrasound Measurements in Hypertrophic Pyloric Stenosis: Don't Let the Numbers Fool You. *The*

Revision History NAITRE MOP

Feb 5, 2019: Removed randomization day
Feb 6, 2019: Addition of William Godwin, MPH to personnel, updated SAE section 8.6, added 2 inclusion criteria. JMB
Feb 8, 2019: Title Change to NAITRE, updated SAE and AE section 8.6 with Burkina guidelines, changed follow-up dates from day 28 after treatment to day 21 JMB
Feb 11, 2019: 3.1.3 removed key informant system based on the day of enrollment
Feb 14, 2019: Removed "birthweight" as inclusion criteria. It is now "weight". JMB
Feb 15, 2019: Addition of follow-up at 1 week and 4 weeks after IHPS Surgery. Change from >3mm pyloric stenosis thickness to >4mm for transfer guidelines. Removal of transverse diameter measurement for pyloric stenosis. Addition of any child with normal pyloric measurements but no food is passing will be transferred.
August 13, 2019: Only require one visit 21 days post treatment rather than 3 weekly home visits post treatment. (Chapter 5 table 1: deleted day 7 and day 14. Chapter 5.2.)
August 21, 2019: addition of Zijun Liu
October 3, 2019: addition of Emily Colby
March 16,2020: addition of section 5.5 passive surveillance JMB

Generated by mordorburkina, Sep 14, 2021 14:50
Questionnaire created by ying, Feb 05, 2019 14:36
Last modified by mangoja8, Mar 03, 2021 22:35

Shared with:
wgodwin28 last edited 5/19/2020 5:58:32 PM
mordorburkina last edited 2/4/2020 7:08:47 PM
klaus last edited 3/25/2019 3:23:52 PM
Alphonse1 (never edited)
mangoja8 last edited 3/4/2021 2:35:46 AM

Sections: 1, Sub-sections: 1, Questions: 20.
Questions with enabling conditions: 17
Questions with validation conditions:6
Rosters: 0
Variables: 5

Survey Solutions

1-NAITRE - Éligibilité

SURVEY IDENTIFICATION INFORMATION
QUESTIONNAIRE DESCRIPTION

ÉLIGIBILITÉ

Sub-sections: 1, No rosters, Questions: 20, Static texts: 4, Variables: 5.

APPENDIX A — ENABLING CONDITIONS

APPENDIX B — CATEGORIES

APPENDIX C — VARIABLES

APPENDIX D — CATEGORIES FILTERS

LEGEND

SURVEY IDENTIFICATION INFORMATION QUESTIONNAIRE DESCRIPTION

Basic information

Title 1-NAITRE - Éligibilité

ÉLIGIBILITÉ

Tap to record date	DATE: CURRENT TIME `startDate`
Did you pre-screen the child for jaundice, age, and weight?<br>E `IsAnswered(startDate)`	SINGLE-SELECT `prescreen`<br>01 ○ Yes<br>02 ○ No

STATIC TEXT

E `prescreen == 2`

Please pre-screen child for jaundice, age, weight first!

Region of residence<br>E `prescreen == 1`	SINGLE-SELECT `region`<br>01 ○ Centre<br>02 ○ Boucle du Mouhoun<br>03 ○ Cascade<br>04 ○ Centre Ouest<br>05 ○ Haut-Bassin<br>06 ○ None of the above
District of residence<br>F @optioncode.InList(1,2) ? region==1 : @optioncode.InList(3,6) ? region==2 : @optioncode.InList(4) ? region==3 : @optioncode.InList(5) ? region==4 : @optioncode.InList(7,8,10) ? region==5 : true<br>E `region.InRange(1,5)`	SINGLE-SELECT `district`<br>01 ○ Sig-Noghin<br>02 ○ Nongremasson<br>03 ○ Nouna<br>04 ○ Banfora<br>05 ○ Koudougou<br>06 ○ Dedougou<br>07 ○ Do<br>08 ○ Dafra<br>10 ○ Karangasso Vigue<br>09 ○ None of the above

**Which CSPS?**<br><br>F @optioncode.InList(1,2,3,5) ? district==1 : @optioncode.InList(4) ? district==2 : @optioncode.InList(6,7,8) ? district==3 : @optioncode.InList(9,10,11,12,13,14,34,35) ? district==4 : @optioncode.InLis<br>And 241 other symbols [1]<br><br>E `district.InList(1,2,3,4,5,6,7,8,10)`	SINGLE-SELECT — q_2_1_6<br>01 ○ CSPS de Bissighin<br>02 ○ CSPS de Bassingo<br>03 ○ CSPS de Yagma<br>04 ○ CSPS de Polesgo<br>05 ○ CSPS Centre medial du secteur 15<br>06 ○ CSPS communal 1<br>07 ○ CSPS communal 2<br>08 ○ CSPS notre dame de l'esperance<br>09 ○ Banfora secteur 8<br>10 ○ Niangoloko secteur 3 n2<br>11 ○ Flantama<br>12 ○ Niangoloko secteur 3<br>13 ○ Niangoloko CM<br>14 ○ Centre Medical Urbain de Banfora<br>15 ○ Centre Medica lde Koudougou<br>16 ○ CSPS Secteur 5 Koudougou<br><br>And 29 other symbols [1]
**Village of residence**<br><br>F @optioncode.InList(4,5) ? q_2_1_6==1 : @optioncode.InList(1,2,3) ? q_2_1_6==2 : @optioncode.InList(6,7,8,9) ? q_2_1_6==3 : @optioncode.InList(12,13,14,15,16,17) ? q_2_1_6==4 : @optioncode.InList(10,11<br>And 1738 other symbols [2]<br><br>E `q_2_1_6.InRange(1,30) | q_2_1_6.InRange(32,45)`	SINGLE-SELECT: COMBO BOX — village<br>001 ○ Bassinko<br>002 ○ Dar Salam<br>003 ○ Silmiougou<br>004 ○ Bissghin<br>005 ○ Yagma<br>006 ○ Yagm koudogo<br>007 ○ Nab-Ziguinima<br>008 ○ Camp Peulh<br>009 ○ Trame d'accueil<br>010 ○ Zone 8<br>011 ○ Zone 9<br>012 ○ Polesgo<br>013 ○ Sambin-Est<br>014 ○ Sambin-Ouest<br>015 ○ Wapassi<br>016 ○ Silmissin<br><br>And 231 other symbols [2]
**Enter village**<br><br>E `village==999`	TEXT — villageOther<br>..
**What is your child's date of birth?**<br><br>E `prescreen == 1`<br>V1 `self < startDate`<br>M1 This cannot be the birthdate, please check!	DATE — childBirthdate<br>..
**Is the child at least 2500g?**<br><br>E `prescreen == 1`	SINGLE-SELECT — childWeight<br>01 ○ Yes<br>02 ○ No

Able to feed orally? This means that the child does not vomit after every feed and can keep food down.<br>E `prescreen == 1`<br>V1 `self==1`<br>M1 Please refer the child to the CSPS for suspicion of IHPS	SINGLE-SELECT `feedOrally`<br>01 ○ Yes<br>02 ○ No
Will you be available in zone d'étude for the next 6 months?<br>E `prescreen == 1`	SINGLE-SELECT `stay6months`<br>01 ○ Yes<br>02 ○ No<br>03 ○ I don't know
Allergy to azalides?<br>E `prescreen == 1`	SINGLE-SELECT `allergyAzalides`<br>01 ○ Yes<br>02 ○ No
Has your child participated in MORDOR-Longitudinal study?<br>E `prescreen == 1 && district == 3`	SINGLE-SELECT `otherStudy`<br>01 ○ Yes<br>02 ○ No

STATIC TEXT

E `village==103 || region==6 || district==9 ||q_2_1_6==31 || feedOrally==2 ||stay6months==2 || stay6months==3 ||startDate > childBirthdate.Value.AddDays(28) ||startDate < childBirthdate.Value.AddDays` And 99 other symbols [2]

Child is not eligible

Consent<br>E `feedOrally==1 && stay6months==1 &&childBirthdate.Value.AddDays(8) < startDate &&childBirthdate.Value.AddDays(28) > startDate &&allergyAzalides == 2 &&(district.InList(1,2,4,5,6,7,8,10) && (village` And 245 other symbols [1]	SINGLE-SELECT `consent`<br>01 ○ Yes<br>02 ○ No

STATIC TEXT

E `consent == 1`

Give copy of consent to mother!

Please give the child a study ID, give the mom the study card, and scan the code<br>E `consent==1`	SINGLE-SELECT `childIDmode`<br>01 ○ Scan the code<br>02 ○ Code can't be scanned
Scan the ID<br>E `childIDmode==1`<br>V1 `self.ToUpper().ToCharArray()[0]=='N' && /* must atrt with 'N' */ n>=0 && n<=32767 && /* 32768 IDs */ self.ToUpper().ToCharArray()[3]==(int)chks /* 4th digit is checksum */`<br>M1 Invalid ID	BARCODE `childIDbarcode`
Enter the ID<br>E `childIDmode==2`<br>V1 `self.ToUpper().ToCharArray()[0]=='N' && /* must atrt with 'N' */ n>=0 && n<=32767 && /* 32768 IDs */ self.ToUpper().ToCharArray()[3]==(int)chks /* 4th digit is checksum */`<br>M1 Invalid ID	TEXT `childIDmanual`<br>..

ÉLIGIBILITÉ

VERIFICATION

E `IsAnswered(childIDmanual)`

**Enter the letters in the code**<br>V1 `self.ToUpper().Substring(0,1) == childIDmanual.ToUpper().Substring(0,1) && self.ToUpper().Substring(1,1) == childIDmanual.ToUpper().Substring(3,1)`<br>M1 Not the same code ! - Please verify !	TEXT childIDverification<br>..
**Enter the numbers in the code**<br>V1 `self.ToUpper().Substring(0,2) == childIDmanual.ToUpper().Substring(1,2) && self.ToUpper().Substring(2,3) == childIDmanual.ToUpper().Substring(4,3)`<br>M1 Not the same code ! - Please verify !	TEXT childIDverification1<br>..
VARIABLE<br>`IsAnswered(childIDbarcode) ? childIDbarcode : childIDmanual`	STRING childID
VARIABLE<br>`(childID.ToUpper().ToCharArray()[1]-48-1)*10000 + (childID.ToUpper().ToCharArray()[2]-48)*1000 + (childID.ToUpper().ToCharArray()[4]-48)*100 + (childID.ToUpper().ToCharArray()[5]-48)*10 + (child`<br>And 33 other symbols [1]	LONG n
VARIABLE<br>`n/10+1`	LONG rc
VARIABLE<br>`n%10+1`	LONG c
VARIABLE<br>`new int[]{ (int)checksum[(int)rc].d1, (int)checksum[(int)rc].d2, (int)checksum[(int)rc].d3, (int)checksum[(int)rc].d4, (int)checksum[(int)rc].d5, (int)checksum[(int)rc].d6,`<br>And 133 other symbols [2]	LONG chks
**Please take a picture of child's ID number stuck inside the carnet**<br>E `consent == 1`	PICTURE photoID

STATIC TEXT

E `consent == 1`

Please make sure child is sent to Baseline form station!

APPENDIX A — ENABLING CONDITIONS

[1] consent: Consent

Enablement Condition:

```
feedOrally==1 && stay6months==1 &&childBirthdate.Value.AddDays(8) < startDate &&childBirthdate.Value.AddDays(28) >
startDate &&allergyAzalides == 2 &&(district.InList(1,2,4,5,6,7,8,10) && (village.InRange(1,102) || village==999
|| village.InRange(104,250)) ||(district==3 && otherStudy==2 && (village.InRange(1,102) ||
village.InRange(104,250)))) &&region.InRange(1,5) &&(q_2_1_6.InRange(1,30) || q_2_1_6.InRange(32,45))
&&childWeight==1
```

[2] : Child is not eligible

Enablement Condition:

```
village==103 || region==6 || district==9 ||q_2_1_6==31 || feedOrally==2 ||stay6months==2 || stay6months==3
||startDate > childBirthdate.Value.AddDays(28) ||startDate < childBirthdate.Value.AddDays(8) ||allergyAzalides ==
1 || otherStudy == 1 || childWeight == 2 ||(village==999 && district==3)
```

APPENDIX B — CATEGORIES

[1] q_2_1_6: Which CSPS?
Categories: 1:CSPS de Bissighin, 2:CSPS de Bassingo, 3:CSPS de Yagma, 4:CSPS de Polesgo, 5:CSPS Centre medial du secteur 15, 6:CSPS communal 1, 7:CSPS communal 2, 8:CSPS notre dame de l'esperance, 9:Banfora secteur 8, 10:Niangoloko secteur 3 n2, 11:Flantama, 12:Niangoloko secteur 3, 13:Niangoloko CM, 14:Centre Medical Urbain de Banfora, 15:Centre Medica lde Koudougou, 16:CSPS Secteur 5 Koudougou, 17:CSPS Secteur 8 Koudougou, 18:CSPS Secteur 9 Koudougou, 19:CSPS Secteur 10 Koudougou, 20:CSPS Secteur 7 Koudougou, 21:CMU Dedougou, 22:CSPS Communal de Dedougou, 23:Accart-Ville, 24:Colma 1, 25:Lafiabougou, 26:Sakaby, 27:CSPS Secteur 24, 28:CSPS Guimbi, 29:CSPS Sarfalao, 30:CSPS Ouezzin VIle, 32:CSPS de Tchériba, 33:CSPS Ouarkoye, 34:CSPS Diabarokoko, 35:CSPS Secteur 5 Niangoloko, 36:CSPS Ramongho, 37:CSPS Bolomakote, 38:CSPS Karangasso sambla, 39:CSPS Toussiana, 40:CSPS Dan, 41:CSPS Deguelin, 42:CSPS Diosso, 43:CSPS Karangasso Vigue, 44:CSPS Poya, 45:CSPS Soumousso, 31:None of the above

[2] village: Village of residence
Categories: 1:Bassinko, 2:Dar Salam, 3:Silmiougou, 4:Bissghin, 5:Yagma, 6:Yagm koudogo, 7:Nab-Ziguinima, 8:Camp Peulh, 9:Trame d'accueil, 10:Zone 8, 11:Zone 9, 12:Polesgo, 13:Sambin-Est, 14:Sambin-Ouest, 15:Wapassi, 16:Silmissin, 17:Tangzougou, 21:Secteur 1, 22:Secteur 2, 23:Secteur 3, 24:Secteur 4, 25:Secteur 5, 26:Secteur 6, 27:Secteur 7, 28:Secteur 8, 29:Secteur 9, 30:Secteur 10, 31:Secteur 11, 32:Secteur 12, 33:Secteur 18, 34:Secteur 19, 35:Secteur 20, 36:Secteur 22, 37:Secteur 30, 38:Korona, 39:Djanabana, 40:Kiribina, 41:Zone Tabou, 42:Camana Gouin, 43:Camana Peulh, 44:Touhoni, 45:Touhoni Peulh, 46:Diakora, 47:Djolena, 48:Mitieridg, 49:Pegwende, 50:Camp Mossi, 51:Kakoumana, 52:Kossara, 53:Tangora, 54:Gnagnana, 55:GUY, 56:Kassou 1, 57:Toega, 58:Noakuy, 59:Badala, 60:Idristenga, 61:Noraogtenga, 62:Moundasso, 63:Somyaltenga, 65:Secteur 3 -N2, 66:Secteur 4 -FL, 67:Secteur 5 -FL, 68:Secteur 6 -FL, 69:Secteur 7 -FL, 70:Secteur 1 -N1, 71:Secteur 3 -N1, 72:Secteur 2 -NC, 73:Secteur 6 -NC, 74:Secteur 7 -NC, 75:Secteur 1 -BA, 76:Secteur 2 -BA, 77:Secteur 3 -BA, 78:Secteur 2 -K, 79:Secteur 3 -K, 80:Secteur 5 -K5, 81:Secteur 6 -K7, 82:Secteur 7 -K7, 83:Secteur 1 -CM, 84:Secteur 2 -CM, 85:Secteur 4 -CM, 86:Secteur 5 -CM, 87:Secteur 3 -DE, 88:Secteur 6 -DE, 89:Secteur 9 -AC, 90:Secteur 10 -AC, 91:Secteur 24, 92:Secteur 33, 93:Non lotie du Sect 24, 94:Secteur 4 -GU, 95:Secteur 5 -GU, 96:Secteur 17 -SA, 97:Secteur 18 -SA, 98:Secteur 26 -SA, 99:Non lotie de Sarfalao, 100:Secteur 32, 101:Secteur 15, 102:Non lotie de Ouezinville, 104:Tcheriba, 105:Banouba, 106:Yeyon, 107:Djissasso, 108:Kana, 109:Ouezala, 110:Sao, 111:Sirakele, 112:Ouarkoye, 113:Bekuy, 114:Fouakuy, 115:Koena, 116:Lokinde, 117:Oue, 118:Perakuy, 119:Syn, 120:Pouankuy, 121:Diarabakoko, 122:Korokora, 123:Tiempagora, 124:Diounouna, 125:Katiembara, 126:Poumpoura, 127:Niarebama, 128:Bomboura, 129:Dionouna Peulh, 130:Tagnana, 131:Wintiera, 132:Diaraba 2, 133:Niarabara2, 134:Sect 1 - NIA, 135:Sect 2 - NIA, 136:Sect 5 - NIA, 137:Honk Kong, 138:Ouattara, 139:Fontaine, 140:Souley, 141:Sect 4 - NIA, 142:Camp Peulh - 2, 143:Fleso, 144:Ramongo, 145:Tanghin, 146:Kolonkande, 147:Bayandi palgo, 148:Bayandi tanghin, 149:Ramonkodogo, 150:Sect 6 - BOL, 151:Sect 27 - BOL, 152:Ksambla, 153:Mangafesso, 154:Koumbadougou, 155:Banakorosso, 156:Diofoloma, 157:Yorokofesso, 158:Toussiana, 159:Nianaba, 160:Wempa 1, 161:Wempa 2, 162:Guapegue, 163:Mou 1, 164:Mou 2, 165:kelia, 166:yoya, 167:Gnaware, 168:Pounya, 169:Kobo, 170:Kououkan, 171:Dougoussin, 172:Koignon, 173:Tapokodeni, 174:Gouindegue, 175:Bouko, 176:Ganwake, 177:Logo, 178:Pognon, 179:Simissian, 180:Safoya, 181:Sanglagnon, 182:Dan, 183:Dergouan, 184:Dingara-Dougou, 185:Mandiasso, 186:Peguina, 187:Soumanguina, 188:Signoghin, 189:Parama 1, 190:Parama 2, 191:Toukoro, 192:Lampabila, 193:Torosso/Gneneta, 194:Gwele/Kabeledaga, 195:Sandinga/Dombio, 196:Koudougou, 197:Nabdogo, 198:Benkadi, 199:Sagoma, 200:DIOSSO, 201:Bres, 202:Bio, 203:Toubadinga, 204:Dangoue, 205:Djata, 206:Bonle, 207:KOUEREDOUGOU, 208:Koonan, 209:Dakouabon, 210:Anduro, 211:K Vigue, 212:Kountiofan, 213:Gnaoue, 214:Kouremanga Faso, 215:Tiebadiallo Faso, 216:Seye, 217:Pankatioro, 218:Massasso, 219:Boborila, 220:Bassatombo, 221:Kody, 222:Laminetombo, 223:Siramogognan, 224:Woondo, 225:Sorobi, 226:Konaticombo, 227:Sassoun, 228:Kotombo, 229:Vien, 230:Poya, 231:Ouere, 232:Poya-obaga, 233:soumousso, 234:klesso, 235:larama, 236:kimi, 237:bagui 1, 238:bagui 2, 239:kongodjan, 240:koukapougo, 241:signoghin, 242:koko, 243:camp peulh, 244:Larama mossi, 245:larama peulh, 246:koba, 247:yankady, 248:kiebalogo, 249:kodiale, 250:gontou, 103:none of the above, 999:other

APPENDIX C — VARIABLES

[1] n:

(childID.ToUpper().ToCharArray()[1]-48-1)*10000 + (childID.ToUpper().ToCharArray()[2]-48)*1000 + (childID.ToUpper().ToCharArray()[4]-48)*100 + (childID.ToUpper().ToCharArray()[5]-48)*10 + (childID.ToUpper().ToCharArray()[6]-48)

[2] chks:

new int[]{ (int)checksum[(int)rc].d1, (int)checksum[(int)rc].d2, (int)checksum[(int)rc].d3, (int)checksum[(int)rc].d4, (int)checksum[(int)rc].d5, (int)checksum[(int)rc].d6, (int)checksum[(int)rc].d7, (int)checksum[(int)rc].d8, (int)checksum[(int)rc].d9, (int)checksum[(int)rc].d10 }[(int)c-1]

APPENDIX D — CATEGORIES FILTERS

[1] q_2_1_6: Which CSPS?

@optioncode.InList(1,2,3,5) ? district==1 : @optioncode.InList(4) ? district==2 : @optioncode.InList(6,7,8) ? district==3 : @optioncode.InList(9,10,11,12,13,14,34,35) ? district==4 : @optioncode.InList(15,16,17,18,19,20,36) ? district==5 : @optioncode.InList(21,22,32,33) ? district==6 : @optioncode.InList(23,24,25,26,38,39) ? district==7 : @optioncode.InList(27,28,29,30,37) ? district==8 : @optioncode.InRange(40,45) ? district==10 : true

[2] village: Village of residence

@optioncode.InList(4,5) ? q_2_1_6==1 : @optioncode.InList(1,2,3) ? q_2_1_6==2 : @optioncode.InList(6,7,8,9) ? q_2_1_6==3 : @optioncode.InList(12,13,14,15,16,17) ? q_2_1_6==4 : @optioncode.InList(10,11) ? q_2_1_6==5 : @optioncode.InList(21,22,23,24,25,26,27,103) ? q_2_1_6.InList(6,7,8) : @optioncode.InList(38,39,40) ? q_2_1_6==9 : @optioncode.InList(65,41,42,43,44,45) ? q_2_1_6==10 : @optioncode.InList(66,67,68,69) ? q_2_1_6==11 : @optioncode.InList(70,71,46,47) ? q_2_1_6==12 : @optioncode.InList(72,73,74,48,49,50,51) ? q_2_1_6==13 : @optioncode.InList(75,76,77,52,53,54) ? q_2_1_6==14 : @optioncode.InList(78,79,55) ? q_2_1_6==15 : @optioncode.InList(80,56) ? q_2_1_6==16 : @optioncode.InList(28) ? q_2_1_6==17 : @optioncode.InList(29) ? q_2_1_6==18 : @optioncode.InList(30,57) ? q_2_1_6==19 : @optioncode.InList(81,82) ? q_2_1_6==20 : @optioncode.InList(83,84,85,86,58,59,60,61) ? q_2_1_6==21 : @optioncode.InList(87,88,62,63) ? q_2_1_6==22 : @optioncode.InList(89,90,36) ? q_2_1_6==23 : @optioncode.InList(31,37) ? q_2_1_6==24 : @optioncode.InList(33,34,35) ? q_2_1_6==25 : @optioncode.InList(32) ? q_2_1_6==26 : @optioncode.InList(91,92,93) ? q_2_1_6==27 : @optioncode.InList(94,95) ? q_2_1_6==28 : @optioncode.InList(96,97,98,99) ? q_2_1_6==29 : @optioncode.InList(100,101,102) ? q_2_1_6==30 : @optioncode.InRange(104,111) ? q_2_1_6==32 : @optioncode.InRange(112,120) ? q_2_1_6==33 : @optioncode.InRange(121,133) ? q_2_1_6==34 : @optioncode.InRange(133,143) ? q_2_1_6==35 : @optioncode.InRange(144,149) ? q_2_1_6==36 : @optioncode.InRange(150,151) ? q_2_1_6==37 : @optioncode.InRange(152,156) ? q_2_1_6==38 : @optioncode.InRange(157,181) ? q_2_1_6==39 : @optioncode.InRange(182,188) ? q_2_1_6==40 : @optioncode.InRange(189,199) ? q_2_1_6==41 : @optioncode.InRange(200,210) ? q_2_1_6==42 : @optioncode.InRange(211,229) ? q_2_1_6==43 : @optioncode.InRange(230,232) ? q_2_1_6==44 : @optioncode.InRange(233,250) ? q_2_1_6==45 : true

LEGEND

Legend and structure of information in this file

Name of section

Enabling condition for this section

Question title

Type of question, scope

Variable name

Answer options

SECTION 5: OTHER INCOME SOURCES

E s4_other_sources_which.Contains(98)

Duis aute irure dolor in reprehenderit in voluptate velit esse cillum dolore eu fugiat nulla pariatur?

I This refers to family relations

E s3_time_other > 0

V1 s4_rel_leaders_which.Contains(98)

M1 Can not be itself

V2 (s3_time_other_breeding_advice <= (50 - s3_time_art_in-sem_advice)) ||s3_time_other_breeding_advice == 0

M2 This person is not in the list

F optioncode != s5_ignored_option_code

MULTI-SELECT
SCOPE: PREFILLED

s4_rel_leaders_other

01 ☐ Community animal health workers

02 ☐ Private

03 ☐ Government

04 ☐ Livestock keepers association

05 ☐ NGO

And 5 other [13]

Additional information:

"I" — Question instruction

"E" — Enabling condition

"V1" — Validation condition №1

"M1" — Message for validation №1

"F" — Filter in Categorical questions

Link to full set in appendix

Breadcrumbs

Type or roster

Roster Title

CHAPTER 3 IDENTIFICATION /

Roster: LEADER RELATION DETAILS

generated by fixed list:

01 Ward Livestock Officer

02 Village Livestock Officer

99 Other (specify)

List items

Generated by mordorburkina, Sep 14, 2021 14:51
Questionnaire created by klaus, Sep 07, 2018 17:18
Last modified by mangoja8, Apr 09, 2021 13:52

Shared with:
ying last edited 2/5/2019 7:31:06 PM
wgodwin28 last edited 6/4/2020 4:27:30 PM
wgodwin28 last edited 6/4/2020 4:27:30 PM
mordorburkina last edited 10/30/2020 9:55:04 PM
Alphonse1 (never edited)
mangoja8 last edited 4/9/2021 5:52:54 PM

Sections: 3, Sub-sections: 1, Questions: 39.
Questions with enabling conditions: 15
Questions with validation conditions:13
Rosters: 1
Variables: 9

Survey Solutions

2-NAITRE - Visite de base

SURVEY IDENTIFICATION INFORMATION
QUESTIONNAIRE DESCRIPTION

IDENTIFICATION
Sub-sections: 1, No rosters, Questions: 23, Static texts: 2, Variables: 8.

INFANT INFORMATION
No sub-sections, Rosters: 1, Questions: 9, Static texts: 3, Variables: 1.

MATERNAL INFORMATION
No sub-sections, No rosters, Questions: 7, Static texts: 1.

APPENDIX A — CATEGORIES

APPENDIX B — VARIABLES

APPENDIX C — CATEGORIES FILTERS

LEGEND

SURVEY IDENTIFICATION INFORMATION QUESTIONNAIRE DESCRIPTION

Basic information

Title 2-NAITRE - Visite de base

IDENTIFICATION

Tap to record date	DATE: CURRENT TIME `startDate`<br>..
appuyer ici pour enregistrer la position GPS	GPS `gps`<br>_ _ _ _ _ _ _ _ _ _ _ _ _ _ _ _ _ _<br>N<br>_ _ _ _ _ _ _ _ _ _ _ _ _ _ _ _ _ _<br>W<br>_ _ _ _ _ _ _ _ _ _ _ _ _ _ _ _ _ _<br>A
Region of residence	SINGLE-SELECT `region`<br>01 ○ Centre<br>02 ○ Boucle du Mouhoun<br>03 ○ Cascade<br>04 ○ Centre Ouest<br>05 ○ Haut-Bassin
District of residence	SINGLE-SELECT: CASCADING `district`<br>01 ○ Sig-Noghin<br>02 ○ Nongremasson<br>03 ○ Nouna<br>04 ○ Banfora<br>05 ○ Koudougou<br>06 ○ Dedougou<br>07 ○ Do<br>08 ○ Dafra<br>10 ○ Karangasso Vigue
Which CSPS?	SINGLE-SELECT: CASCADING `q_2_1_6`<br>01 ○ CSPS de Bissighin<br>02 ○ CSPS de Bassingo<br>03 ○ CSPS de Yagma<br>04 ○ CSPS de Polesgo<br>05 ○ CSPS Centre medial du secteur 15<br>06 ○ CSPS communal 1<br>07 ○ CSPS communal 2<br>08 ○ CSPS notre dame de l'esperance<br>09 ○ Banfora secteur 8<br>10 ○ Niangoloko secteur 3 n2<br>11 ○ Flantama<br>12 ○ Niangoloko secteur 3<br>13 ○ Niangoloko CM<br>14 ○ Centre Medical Urbain de Banfora<br>15 ○ Centre Medica lde Koudougou<br>16 ○ CSPS Secteur 5 Koudougou<br><br>And 28 other symbols [1]

Village of residence<br><br>F @optioncode.InList(4,5) ? q_2_1_6==1 : @optioncode.InList(1,2,3) ? q_2_1_6==2 : @optioncode.InList(6,7,8,9) ? q_2_1_6==3 : @optioncode.InList(12,13,14,15,16,17) ? q_2_1_6==4 : @optioncode.InList(10,11<br>And 1738 other symbols [1]<br>E q_2_1_6.InRange(1,30) | q_2_1_6.InRange(32,45)	SINGLE-SELECT: COMBO BOX — village<br>001 ○ Bassinko<br>002 ○ Dar Salam<br>003 ○ Silmiougou<br>004 ○ Bissghin<br>005 ○ Yagma<br>006 ○ Yagm koudogo<br>007 ○ Nab-Ziguinima<br>008 ○ Camp Peulh<br>009 ○ Trame d'accueil<br>010 ○ Zone 8<br>011 ○ Zone 9<br>012 ○ Polesgo<br>013 ○ Sambin-Est<br>014 ○ Sambin-Ouest<br>015 ○ Wapassi<br>016 ○ Silmissin<br><br>And 231 other symbols [2]
Enter village<br><br>E village==999	TEXT — villageOther<br><br>..
Child's first name	TEXT — childFirstname<br><br>..
Child's last name	TEXT — childLastname<br><br>..
VARIABLE<br>"blue"	STRING — colorName

STATIC TEXT

E false

Birthdate
Ask for documentation!

%childFirstname%'s ID	SINGLE-SELECT — childIDmode<br>01 ○ Scan the code<br>02 ○ The code can't be scanned
Scan %childFirstname%'s ID<br><br>E childIDmode==1<br>V1 self.ToUpper().ToCharArray()[0]=='N' && /* must atrt with 'N' */ n>=0 && n<=32767 && /* 32768 IDs */ self.ToUpper().ToCharArray()[3]==(int)chks /* 4th digit is checksum */<br>M1 Invalid ID	BARCODE — childIDbarcode
Enter %childFirstname%'s ID<br><br>E childIDmode==2<br>V1 self.ToUpper().ToCharArray()[0]=='N' && /* must atrt with 'N' */ n>=0 && n<=32767 && /* 32768 IDs */ self.ToUpper().ToCharArray()[3]==(int)chks /* 4th digit is checksum */<br>M1 Invalid ID	TEXT — childIDmanual<br><br>..

IDENTIFICATION

VERIFICATION

E `IsAnswered(childIDmanual)`

Enter the letters in the code<br><br>V1 `self.ToUpper().Substring(0,1) == childIDmanual.ToUpper().Substring(0,1) && self.ToUpper().Substring(1,1) == childIDmanual.ToUpper().Substring(3,1)`<br>M1 Not the same code ! - Please verify !	TEXT `childIDverification`<br>..
Enter the numbers in the code<br><br>V1 `self.ToUpper().Substring(0,2) == childIDmanual.ToUpper().Substring(1,2) && self.ToUpper().Substring(2,3) == childIDmanual.ToUpper().Substring(4,3)`<br>M1 Not the same code ! - Please verify !	TEXT `childIDverification1`<br>..
VARIABLE<br>`IsAnswered(childIDbarcode) ? childIDbarcode : childIDmanual`	STRING `childID`
VARIABLE<br>`(childID.ToUpper().ToCharArray()[1]-48-1)*10000 + (childID.ToUpper().ToCharArray()[2]-48)*1000 + (childID.ToUpper().ToCharArray()[4]-48)*100 + (childID.ToUpper().ToCharArray()[5]-48)*10 + (child`<br><br>And 33 other symbols [1]	LONG `n`
VARIABLE<br>`n/10+1`	LONG `rc`
VARIABLE<br>`n%10+1`	LONG `c`
VARIABLE<br>`new int[]{ (int)checksum[(int)rc].d1, (int)checksum[(int)rc].d2, (int)checksum[(int)rc].d3, (int)checksum[(int)rc].d4, (int)checksum[(int)rc].d5, (int)checksum[(int)rc].d6,`<br><br>And 133 other symbols [2]	LONG `chks`
VARIABLE<br>`new int[]{ (int)tl[(int)rc].d1, (int)tl[(int)rc].d2, (int)tl[(int)rc].d3, (int)tl[(int)rc].d4, (int)tl[(int)rc].d5, (int)tl[(int)rc].d6, (int)tl[(int)rc].d7, (int)tl[(i`<br><br>And 73 other symbols [3]	LONG `tlix`
VARIABLE<br>`new string[]{"RR","SS","TT","UU","WW","XX","YY","ZZ"}[(int)tlix-1]`	STRING `treatmentLetter`

STATIC TEXT

E `false`

TL %treatmentLetter%

Birthdate - Year<br><br>V1 `self.InRange(1900, 2020) || self==9999`<br>M1 L'année doit être entre 1900 et 2020	NUMERIC: INTEGER `anneeN`<br>_ _<br>SPECIAL VALUES<br>9999 Ne sait pas

**Birthdate - Month**<br><br>E `anneeN!=9999`	SINGLE-SELECT `moisN`<br>01 ○ January<br>02 ○ February<br>03 ○ March<br>04 ○ April<br>05 ○ May<br>06 ○ June<br>07 ○ July<br>08 ○ August<br>09 ○ September<br>10 ○ October<br>11 ○ November<br>12 ○ Décember<br>99 ○ Don't know
**Birthdate - day**<br><br>F (@optioncode==30 && moisN!=2) | | (@optioncode==31 && moisN.InList(1,3,5,7,8,10,12)) | | @optioncode.InRange(1,29) | | @optioncode==99<br>E `moisN.InRange(1,12)`	SINGLE-SELECT `jourN`<br>01 ○ 1<br>02 ○ 2<br>03 ○ 3<br>04 ○ 4<br>05 ○ 5<br>06 ○ 6<br>07 ○ 7<br>08 ○ 8<br>09 ○ 9<br>10 ○ 10<br>11 ○ 11<br>12 ○ 12<br>13 ○ 13<br>14 ○ 14<br>15 ○ 15<br>16 ○ 16<br><br>And 16 other symbols [3]
**%childFirstname%'s age:**<br><br>E `anneeN==9999`	NUMERIC: INTEGER `reportedAge`<br>_ _ _ _ _ _ _ _ _ _ _ _ _ _ _ _ _ _ _ _
**How did you validate the date of birth?**	SINGLE-SELECT `ddnSource`<br>01 ○ Vaccination card<br>02 ○ Birth certificate<br>03 ○ CNIB / Passport<br>04 ○ Someone told me<br>05 ○ Other documents
**%childFirstname%'s birthweight**<br><br>E `treatmentLetter!=null`	SINGLE-SELECT `birthweight`<br>01 ○ Enter birthweight<br>02 ○ Birthweight unknown
**Birth weight (in grams)**<br><br>E `birthweight == 1`<br>V1 `self.InRange(1500,7000)`<br>M1 This can't be %childFirstname%'s birth weight in grams. Please verify!	NUMERIC: DECIMAL `q_2_1_2`<br>_ _ _ _ _ _ _ _ _ _ _ _ _ _ _ _ _ _ _ _

What is the name of %childFirstname%'s mother/guardian?<br><br>I Enter first and last name<br>E treatmentLetter!=null	TEXT motherFullname<br><br>..
What is the name of %childFirstname%'s father?<br><br>I Enter first and last name<br>E treatmentLetter!=null	TEXT fatherFullname<br><br>..

INFANT INFORMATION

%childFirstname%'s sex	SINGLE-SELECT q_2_1_1<br>01 ○ Masculin<br>02 ○ Feminin

INFANT INFORMATION

Roster: LENGTH

generated by fixed list

R_length

01 1

02 2

03 3

%childFirstname%'s length in cm<br>V1 `self.InRange(30,80)`<br>M1 This cannot be the length for this child. Please verify!<br>V2 `@rowcode==2?self.InRange(R_length[1].length_m*0.9,R_length[1].length_m*1.1):true`<br>M2 The two measurements are very different! Please verify!<br>V3 `@rowcode==3?self.InRange(R_length[1].length_m*0.9,R_length[1].length_m*1.1) && self.InRange(R_length[2].length_m*0.9,R_length[2].length_m*1.1):true`<br>M3 The 3 measurements differ too much. Please verify!<br>V4 `@rowcode==3?self!=R_length[1].length_m && self!=R_length[2].length_m:true`<br>M4 All three measurements are the same. Are you sure?	NUMERIC: DECIMAL length_m<br>_______________
VARIABLE<br>`(R_length.Sum(x => (double)x.length_m) / 3).ToString ("#.#")`	STRING length

STATIC TEXT

E `R_length.Count(x => IsAnswered(x.length_m)) == 3`

Length (average) = %length%

%childFirstname%'s weight	NUMERIC: DECIMAL weight<br>_______________
Which units?<br>E `IsAnswered(weight)`<br>V1 `self==1 ? weight.InRange(2.49,7) : weight.InRange(5.5,15.45)`<br>M1 This can't be the weight in %self%!	SINGLE-SELECT weightUnit<br>01 ○ kg<br>02 ○ lbs
MUAC in cm<br>V1 `self>0 && self<20`<br>M1 This can't be %childFirstname%'s MUAC in cm!	NUMERIC: DECIMAL MUAC<br>_______________

STATIC TEXT

E `MUAC == weight`

Veuillez vérifier les mesures de MUAC et de poids, ils sont identiques!

STATIC TEXT

E `MUAC < 11.5 && false`

Refer %childFirstname% to the clinic!

Pregnancy type	SINGLE-SELECT q_2_1_3<br>01 ○ Singleton<br>02 ○ Multiple
Born at the health center?	SINGLE-SELECT q_2_1_4<br>01 ○ Yes<br>02 ○ No
Type of feeding for child	SINGLE-SELECT childFeeding<br>01 ○ Breast milk<br>02 ○ Formula milk<br>03 ○ Mix of breast milk and formula
Time of breastfeeding initiation<br>E `childFeeding == 1 || childFeeding == 3`	SINGLE-SELECT breastfeedInit2<br>01 ○ Immediately<br>02 ○ Not immediately

MATERNAL INFORMATION

Question	Response
Mom's age<br>W1 `self.InRange(13, 60)`<br>M1 Are you sure the age is correct?	NUMERIC: INTEGER q_2_2_1<br>_ _
Education level	SINGLE-SELECT q_2_2_2<br>01 ○ None<br>02 ○ Primary<br>03 ○ Secondary<br>04 ○ More than secondary
Number of alive children<br>I Do not include %childFirstname%<br>V1 `self.InRange(0, 20)`<br>M1 Must be between 0 and 20	NUMERIC: INTEGER q_2_2_3<br>_ _
How many prenatal visits did you have?	SINGLE-SELECT prenatalVisits<br>01 ○ Enter number<br>02 ○ Unknown
Number of prenatal visits<br>E `prenatalVisits == 1`<br>W1 `self.InRange(0, 20)`<br>M1 Please check this number!	NUMERIC: INTEGER q_2_2_4<br>_ _
How many times have you been pregnant?<br>I ne pas inclure cette grossesse	SINGLE-SELECT preg<br>01 ○ Enter number<br>02 ○ Don't know
Number of times pregnant<br>E `preg == 1`<br>W1 `self.InRange(0,100)`<br>M1 Please check this number!	NUMERIC: INTEGER pregNum<br>_ _

STATIC TEXT

E `IsAnswered(preg)`

Please continue to the Treatment survey!

APPENDIX A — CATEGORIES

[1] q_2_1_6: Which CSPS?
Categories: 1:CSPS de Bissighin, 2:CSPS de Bassingo, 3:CSPS de Yagma, 4:CSPS de Polesgo, 5:CSPS Centre medial du secteur 15, 6:CSPS communal 1, 7:CSPS communal 2, 8:CSPS notre dame de l'esperance, 9:Banfora secteur 8, 10:Niangoloko secteur 3 n2, 11:Flantama, 12:Niangoloko secteur 3, 13:Niangoloko CM, 14:Centre Medical Urbain de Banfora, 15:Centre Medica lde Koudougou, 16:CSPS Secteur 5 Koudougou, 17:CSPS Secteur 8 Koudougou, 18:CSPS Secteur 9 Koudougou, 19:CSPS Secteur 10 Koudougou, 20:CSPS Secteur 7 Koudougou, 21:CMU Dedougou, 22:CSPS Communal de Dedougou, 23:Accart-Ville, 24:Colma 1, 25:Lafiabougou, 26:Sakaby, 27:CSPS Secteur 24, 28:CSPS Guimbi, 29:CSPS Sarfalao, 30:CSPS Ouezzin VIle, 32:CSPS de Tcheriba, 33:CSPS Ouarkoye, 34:CSPS Diabarokoko, 35:CSPS Secteur 5 Niangoloko, 36:CSPS Ramongho, 37:CSPS Bolomakote, 38:CSPS Karangasso sambla, 39:CSPS Toussiana, 40:CSPS Dan, 41:CSPS Deguelin, 42:CSPS Diosso, 43:CSPS Karangasso Vigue, 44:CSPS Poya, 45:CSPS Soumousso

[2] village: Village of residence
Categories: 1:Bassinko, 2:Dar Salam, 3:Silmiougou, 4:Bissghin, 5:Yagma, 6:Yagm koudogo, 7:Nab-Ziguinima, 8:Camp Peulh, 9:Trame d'accueil, 10:Zone 8, 11:Zone 9, 12:Polesgo, 13:Sambin-Est, 14:Sambin-Ouest, 15:Wapassi, 16:Silmissin, 17:Tangzougou, 21:Secteur 1, 22:Secteur 2, 23:Secteur 3, 24:Secteur 4, 25:Secteur 5, 26:Secteur 6, 27:Secteur 7, 28:Secteur 8, 29:Secteur 9, 30:Secteur 10, 31:Secteur 11, 32:Secteur 12, 33:Secteur 18, 34:Secteur 19, 35:Secteur 20, 36:Secteur 22, 37:Secteur 30, 38:Korona, 39:Djanabana, 40:Kiribina, 41:Zone Tabou, 42:Camana Gouin, 43:Camana Peulh, 44:Touhoni, 45:Touhoni Peulh, 46:Diakora, 47:Djolena, 48:Mitieridg, 49:Pegwende, 50:Camp Mossi, 51:Kakoumana, 52:Kossara, 53:Tangora, 54:Gnagnana, 55:GUY, 56:Kassou 1, 57:Toega, 58:Noakuy, 59:Badala, 60:Idristenga, 61:Noraogtenga, 62:Moundasso, 63:Somyaltenga, 65:Secteur 3 -N2, 66:Secteur 4 -FL, 67:Secteur 5 -FL, 68:Secteur 6 -FL, 69:Secteur 7 -FL, 70:Secteur 1 -N1, 71:Secteur 3 -N1, 72:Secteur 2 -NC, 73:Secteur 6 -NC, 74:Secteur 7 -NC, 75:Secteur 1 -BA, 76:Secteur 2 -BA, 77:Secteur 3 -BA, 78:Secteur 2 -K, 79:Secteur 3 -K, 80:Secteur 5 -K5, 81:Secteur 6 -K7, 82:Secteur 7 -K7, 83:Secteur 1 -CM, 84:Secteur 2 -CM, 85:Secteur 4 -CM, 86:Secteur 5 -CM, 87:Secteur 3 -DE, 88:Secteur 6 -DE, 89:Secteur 9 -AC, 90:Secteur 10 -AC, 91:Secteur 24, 92:Secteur 33, 93:Non lotie du Sect 24, 94:Secteur 4 -GU, 95:Secteur 5 -GU, 96:Secteur 17 -SA, 97:Secteur 18 -SA, 98:Secteur 26 -SA, 99:Non lotie de Sarfalao, 100:Secteur 32, 101:Secteur 15, 102:Non lotie de Ouezinville, 104:Tcheriba, 105:Banouba, 106:Yeyon, 107:Djissasso, 108:Kana, 109:Ouezala, 110:Sao, 111:Sirakele, 112:Ouarkoye, 113:Bekuy, 114:Fouakuy, 115:Koena, 116:Lokinde, 117:Oue, 118:Perakuy, 119:Syn, 120:Pouankuy, 121:Diarabakoko, 122:Korokora, 123:Tiempagora, 124:Diounouna, 125:Katiembara, 126:Poumpoura, 127:Niarebama, 128:Bomboura, 129:Dionouna Peulh, 130:Tagnana, 131:Wintiera, 132:Diaraba 2, 133:Niarabara2, 134:Sect 1 - NIA, 135:Sect 2 - NIA, 136:Sect 5 - NIA, 137:Honk Kong, 138:Ouattara, 139:Fontaine, 140:Souley, 141:Sect 4 - NIA, 142:Camp Peulh - 2, 143:Fleso, 144:Ramongo, 145:Tanghin, 146:Kolonkande, 147:Bayandi palgo, 148:Bayandi tanghin, 149:Ramonkodogo, 150:Sect 6 - BOL, 151:Sect 27 - BOL, 152:Ksambla, 153:Mangafesso, 154:Koumbadougou, 155:Banakorosso, 156:Diofoloma, 157:Yorokofesso, 158:Toussiana, 159:Nianaba, 160:Wempa 1, 161:Wempa 2, 162:Guapegue, 163:Mou 1, 164:Mou 2, 165:kelia, 166:yoya, 167:Gnaware, 168:Pounya, 169:Kobo, 170:Kououkan, 171:Dougoussin, 172:Koignon, 173:Tapokodeni, 174:Gouindegue, 175:Bouko, 176:Ganwake, 177:Logo, 178:Pognon, 179:Simissian, 180:Safoya, 181:Sanglagnon, 182:Dan, 183:Dergouan, 184:Dingara-Dougou, 185:Mandiasso, 186:Peguina, 187:Soumanguina, 188:Signoghin, 189:Parama 1, 190:Parama 2, 191:Toukoro, 192:Lampabila, 193:Torosso/Gneneta, 194:Gwele/Kabeledaga, 195:Sandinga/Dombio, 196:Koudougou, 197:Nabdogo, 198:Benkadi, 199:Sagoma, 200:DIOSSO, 201:Bres, 202:Bio, 203:Toubadinga, 204:Dangoue, 205:Djata, 206:Bonle, 207:KOUEREDOUGOU, 208:Koonan, 209:Dakouabon, 210:Anduro, 211:K Vigue, 212:Kountiofan, 213:Gnaoue, 214:Kouremanga Faso, 215:Tiebadiallo Faso, 216:Seye, 217:Pankatioro, 218:Massasso, 219:Boborila, 220:Bassatombo, 221:Kody, 222:Laminetombo, 223:Siramogognan, 224:Woondo, 225:Sorobi, 226:Konaticombo, 227:Sassoun, 228:Kotombo, 229:Vien, 230:Poya, 231:Ouere, 232:Poya-obaga, 233:soumousso, 234:klesso, 235:larama, 236:kimi, 237:bagui 1, 238:bagui 2, 239:kongodjan, 240:koukapougo, 241:signoghin, 242:koko, 243:camp peulh, 244:Larama mossi, 245:larama peulh, 246:koba, 247:yankady, 248:kiebalogo, 249:kodiale, 250:gontou, 103:none of the above, 999:other

[3] jourN: Birthdate - day
Categories: 1:1, 2:2, 3:3, 4:4, 5:5, 6:6, 7:7, 8:8, 9:9, 10:10, 11:11, 12:12, 13:13, 14:14, 15:15, 16:16, 17:17, 18:18, 19:19, 20:20, 21:21, 22:22, 23:23, 24:24, 25:25, 26:26, 27:27, 28:28, 29:29, 30:30, 31:31, 99:Ne sait pas

APPENDIX B — VARIABLES

[1] n:

(childID.ToUpper().ToCharArray()[1]-48-1)*10000 + (childID.ToUpper().ToCharArray()[2]-48)*1000 + (childID.ToUpper().ToCharArray()[4]-48)*100 + (childID.ToUpper().ToCharArray()[5]-48)*10 + (childID.ToUpper().ToCharArray()[6]-48)

[2] chks:

new int[]{ (int)checksum[(int)rc].d1, (int)checksum[(int)rc].d2, (int)checksum[(int)rc].d3, (int)checksum[(int)rc].d4, (int)checksum[(int)rc].d5, (int)checksum[(int)rc].d6, (int)checksum[(int)rc].d7, (int)checksum[(int)rc].d8, (int)checksum[(int)rc].d9, (int)checksum[(int)rc].d10 }[(int)c-1]

[3] tlix:

new int[]{ (int)tl[(int)rc].d1, (int)tl[(int)rc].d2, (int)tl[(int)rc].d3, (int)tl[(int)rc].d4, (int)tl[(int)rc].d5, (int)tl[(int)rc].d6, (int)tl[(int)rc].d7, (int)tl[(int)rc].d8, (int)tl[(int)rc].d9, (int)tl[(int)rc].d10 }[(int)c-1]

APPENDIX C — CATEGORIES FILTERS

[1] village: Village of residence

@optioncode.InList(4,5) ? q_2_1_6==1 : @optioncode.InList(1,2,3) ? q_2_1_6==2 : @optioncode.InList(6,7,8,9) ? q_2_1_6==3 : @optioncode.InList(12,13,14,15,16,17) ? q_2_1_6==4 : @optioncode.InList(10,11) ? q_2_1_6==5 : @optioncode.InList(21,22,23,24,25,26,27,103) ? q_2_1_6.InList(6,7,8) : @optioncode.InList(38,39,40) ? q_2_1_6==9 : @optioncode.InList(65,41,42,43,44,45) ? q_2_1_6==10 : @optioncode.InList(66,67,68,69) ? q_2_1_6==11 : @optioncode.InList(70,71,46,47) ? q_2_1_6==12 : @optioncode.InList(72,73,74,48,49,50,51) ? q_2_1_6==13 : @optioncode.InList(75,76,77,52,53,54) ? q_2_1_6==14 : @optioncode.InList(78,79,55) ? q_2_1_6==15 : @optioncode.InList(80,56) ? q_2_1_6==16 : @optioncode.InList(28) ? q_2_1_6==17 : @optioncode.InList(29) ? q_2_1_6==18 : @optioncode.InList(30,57) ? q_2_1_6==19 : @optioncode.InList(81,82) ? q_2_1_6==20 : @optioncode.InList(83,84,85,86,58,59,60,61) ? q_2_1_6==21 : @optioncode.InList(87,88,62,63) ? q_2_1_6==22 : @optioncode.InList(89,90,36) ? q_2_1_6==23 : @optioncode.InList(31,37) ? q_2_1_6==24 : @optioncode.InList(33,34,35) ? q_2_1_6==25 : @optioncode.InList(32) ? q_2_1_6==26 : @optioncode.InList(91,92,93) ? q_2_1_6==27 : @optioncode.InList(94,95) ? q_2_1_6==28 : @optioncode.InList(96,97,98,99) ? q_2_1_6==29 : @optioncode.InList(100,101,102) ? q_2_1_6==30 : @optioncode.InRange(104,111) ? q_2_1_6==32 : @optioncode.InRange(112,120) ? q_2_1_6==33 : @optioncode.InRange(121,133) ? q_2_1_6==34 : @optioncode.InRange(133,143) ? q_2_1_6==35 : @optioncode.InRange(144,149) ? q_2_1_6==36 : @optioncode.InRange(150,151) ? q_2_1_6==37 : @optioncode.InRange(152,156) ? q_2_1_6==38 : @optioncode.InRange(157,181) ? q_2_1_6==39 : @optioncode.InRange(182,188) ? q_2_1_6==40 : @optioncode.InRange(189,199) ? q_2_1_6==41 : @optioncode.InRange(200,210) ? q_2_1_6==42 : @optioncode.InRange(211,229) ? q_2_1_6==43 : @optioncode.InRange(230,232) ? q_2_1_6==44 : @optioncode.InRange(233,250) ? q_2_1_6==45 : true

LEGEND

Legend and structure of information in this file

Name of section

Enabling condition for this section

Question title

Type of question, scope

Variable name

Answer options

SECTION 5: OTHER INCOME SOURCES

E s4_other_sources_which.Contains(98)

Duis aute irure dolor in reprehenderit in voluptate velit esse cillum dolore eu fugiat nulla pariatur?

MULTI-SELECT
SCOPE: PREFILLED

s4_rel_leaders_other

I This refers to family relations
E s3_time_other > 0
V1 s4_rel_leaders_which.Contains(98)
M1 Can not be itself
V2 (s3_time_other_breeding_advice <= (50 - s3_time_art_insem_advice)) ||s3_time_other_breeding_advice == 0
M2 This person is not in the list
F optioncode != s5_ignored_option_code

01 ☐ Community animal health workers
02 ☐ Private
03 ☐ Government
04 ☐ Livestock keepers association
05 ☐ NGO

And 5 other [13]

Additional information:
"I" — Question instruction
"E" — Enabling condition
"V1" — Validation condition №1
"M1" — Message for validation №1
"F" — Filter in Categorical questions

Link to full set in appendix

Breadcrumbs

Type or roster

Roster Title

CHAPTER 3 IDENTIFICATION /
Roster: LEADER RELATION DETAILS
generated by fixed list:

01 Ward Livestock Officer
02 Village Livestock Officer
99 Other (specify)

List items

Generated by mordorburkina, Sep 14, 2021 14:51
Questionnaire created by klaus, Sep 08, 2018 15:08
Last modified by mangoja8, Feb 26, 2021 18:10

Shared with:
wgodwin28 last edited 6/30/2020 5:52:58 PM
ying last edited 11/18/2019 6:11:49 PM
mordorburkina last edited 6/30/2020 4:08:33 PM
Alphonse1 (never edited)
mangoja8 last edited 2/26/2021 10:10:25 PM

Sections: 1, Sub-sections: 2, Questions: 18.
Questions with enabling conditions: 11
Questions with validation conditions:6
Rosters: 0
Variables: 21

Survey Solutions

3-NAITRE - Traitement

SURVEY IDENTIFICATION INFORMATION
QUESTIONNAIRE DESCRIPTION

IDENTIFICATION
Sub-sections: 2, No rosters, Questions: 18, Static texts: 3, Variables: 21.

APPENDIX A — ENABLING CONDITIONS

APPENDIX B — VARIABLES

LEGEND

SURVEY IDENTIFICATION INFORMATION QUESTIONNAIRE DESCRIPTION

Basic information

Title 3-NAITRE - Traitement

IDENTIFICATION

Tap to record date	DATE: CURRENT TIME `startTime`
Child's first name	TEXT `childFirstname`
VARIABLE<br>`"blue"`	STRING `colorName`
%childFirstname%'s sex?	SINGLE-SELECT `childSex`<br>01 ○ Masculin<br>02 ○ Féminin
%childFirstname%'s birthdate?<br>V1 `childBirthdate.Value.AddDays(8) < startTime && childBirthdate.Value.AddDays(28) > startTime`<br>M1 Child must be between 8-28 days old!	DATE `childBirthdate`
%childFirstname%'s ID	SINGLE-SELECT `childIDmode`<br>01 ○ Scan the code<br>02 ○ Code can't be scanned
Scan %childFirstname%'s ID<br>E `childIDmode==1`<br>V1 `self.ToUpper().ToCharArray()[0]=='N' && /* must atrt with 'N' */ n>=0 && n<=32767 && /* 32768 IDs */ self.ToUpper().ToCharArray()[3]==(int)chks /* 4th digit is checksum */`<br>M1 Invalid ID	BARCODE `childIDbarcode`
Enter %childFirstname%'s ID<br>E `childIDmode==2`<br>V1 `self.ToUpper().ToCharArray()[0]=='N' && /* must atrt with 'N' */ n>=0 && n<=32767 && /* 32768 IDs */ self.ToUpper().ToCharArray()[3]==(int)chks /* 4th digit is checksum */`<br>M1 Invalid ID	TEXT `childIDmanual`

IDENTIFICATION

VERIFICATION

E `IsAnswered(childIDmanual)`

Enter the letters in the code<br>V1 `self.ToUpper().Substring(0,1) == childIDmanual.ToUpper().Substring(0,1) && self.ToUpper().Substring(1,1) == childIDmanual.ToUpper().Substring(3,1)`<br>M1 Not the same code ! - Please verify !	TEXT `childIDverification`
Enter the numbers in the code<br>V1 `self.ToUpper().Substring(0,2) == childIDmanual.ToUpper().Substring(1,2) && self.ToUpper().Substring(2,3) == childIDmanual.ToUpper().Substring(4,3)`<br>M1 Not the same code ! - Please verify !	TEXT `childIDverification1`

VARIABLE<br>`IsAnswered(childIDbarcode) ? childIDbarcode : childIDmanual`	STRING childID
VARIABLE<br>`(childID.ToUpper().ToCharArray()[1]-48-1)*10000 + (childID.ToUpper().ToCharArray()[2]-48)*1000 + (childID.ToUpper().ToCharArray()[4]-48)*100 + (childID.ToUpper().ToCharArray()[5]-48)*10 + (child`<br>And 33 other symbols [1]	LONG n
VARIABLE<br>`n/10+1`	LONG rc
VARIABLE<br>`n%10+1`	LONG c
VARIABLE<br>`new int[]{ (int)checksum[(int)rc].d1, (int)checksum[(int)rc].d2, (int)checksum[(int)rc].d3, (int)checksum[(int)rc].d4, (int)checksum[(int)rc].d5, (int)checksum[(int)rc].d6,`<br>And 133 other symbols [2]	LONG chks
VARIABLE<br>`new int[]{ (int)tl[(int)rc].d1, (int)tl[(int)rc].d2, (int)tl[(int)rc].d3, (int)tl[(int)rc].d4, (int)tl[(int)rc].d5, (int)tl[(int)rc].d6, (int)tl[(int)rc].d7, (int)tl[(i`<br>And 73 other symbols [3]	LONG tlix
VARIABLE<br>`new string[]{"RR","SS","TT","UU","WW","XX","YY","ZZ"}[(int)tlix-1]`	STRING treatmentLetter
Can I treat %childFirstname%?<br>E `/* childIDverification.ToUpper() == childIDmanual.ToUpper() (IsAnswered(childIDbarcode)) || (childIDverification.ToUpper().Substring(0,1) == childIDmanual.ToUpper().Substring(0,1) && childIDverificat` And 435 other symbols [1]	SINGLE-SELECT consent<br>01 ○ yes<br>02 ○ no
Why not?<br>E `consent == 2`	TEXT noConsent<br>..
Name of %childFirstname%'s mother/guardian?<br>I Enter first and last name<br>E `consent==1 && false`	TEXT motherFullname<br>..
Name of %childFirstname%'s father?<br>I Enter first and last name<br>E `consent==1 && false`	TEXT fatherFullname<br>..

STATIC TEXT

E `consent==1`

Treatment letter: %treatmentLetter%

%childFirstname%'s weight (kg)<br>I Enter the weight of the child from the baseline form<br>E `consent==1`<br>V1 `self.InRange(2.5,7)`<br>M1 Are you sure %childFirstname% weights %weight% kg?	NUMERIC: DECIMAL weight<br>_ _

VARIABLE<br>`Math.Round(weight.Value*5)/10`	DOUBLE `dose_ml`

STATIC TEXT

E `IsAnswered(weight)`

Treatment dose: %dose_ml% ml (40mg/ml solution d'azithromycine)

Treatment administered ?<br>E `IsAnswered(weight)`	SINGLE-SELECT `isAdministered`<br>01 ○ Yes<br>02 ○ No
Tap to record time<br>E `isAdministered==1`	DATE: CURRENT TIME `treatTime`<br>..
Why was the treatment not administered?<br>E `isAdministered==2`	SINGLE-SELECT `reasonNotAdministered`<br>01 ○ Child absent<br>02 ○ Died<br>03 ○ Sick<br>99 ○ Other
Specify other reason?<br>E `reasonNotAdministered==99`	TEXT `reasonNotAdministeredOther`<br>..

IDENTIFICATION

IMPORTANT DATES FOR FOLLOW UP

E `IsAnswered(consent)`

VARIABLE<br>`startTime.Value.AddDays(14).ToString("dd-MM-yyyy")`	STRING `day21lower`
VARIABLE<br>`startTime.Value.AddDays(21).ToString("dd-MM-yyyy")`	STRING `day21`
VARIABLE<br>`startTime.Value.AddDays(35).ToString("dd-MM-yyyy")`	STRING `day21upper`
VARIABLE<br>`childBirthdate.Value.AddDays(69).ToString("dd-MM-yyyy")`	STRING `day90lower`
VARIABLE<br>`childBirthdate.Value.AddDays(90).ToString("dd-MM-yyyy")`	STRING `day90`
VARIABLE<br>`childBirthdate.Value.AddDays(111).ToString("dd-MM-yyyy")`	STRING `day90upper`
VARIABLE<br>`childBirthdate.Value.AddDays(159).ToString("dd-MM-yyyy")`	STRING `day180lower`
VARIABLE<br>`childBirthdate.Value.AddDays(180).ToString("dd-MM-yyyy")`	STRING `day180`
VARIABLE<br>`childBirthdate.Value.AddDays(201).ToString("dd-MM-yyyy")`	STRING `day180upper`
VARIABLE<br>`childBirthdate.Value.AddDays(344).ToString("dd-MM-yyyy")`	STRING `day365lower`
VARIABLE<br>`childBirthdate.Value.AddDays(365).ToString("dd-MM-yyyy")`	STRING `day365`

VARIABLE	STRING
`childBirthdate.Value.AddDays(386).ToString("dd-MM-yyyy")`	`day365upper`

STATIC TEXT

Dates for follow up:
Visit 1: %day21% (%day21lower% to %day21upper%)
Day 90: %day90% (%day90lower% to %day90upper%)
Day 180: %day180% (%day180lower% to %day180upper%)
Day 365: %day365% (%day365lower% to %day365upper%)

APPENDIX A — ENABLING CONDITIONS

[1] consent: Can I treat %childFirstname%?
Enablement Condition:

```
/* childIDverification.ToUpper() == childIDmanual.ToUpper()
(IsAnswered(childIDbarcode)) ||
(childIDverification.ToUpper().Substring(0,1) == childIDmanual.ToUpper().Substring(0,1) &&
childIDverification.ToUpper().Substring(1,1) == childIDmanual.ToUpper().Substring(3,1) &&
childIDverification1.ToUpper().Substring(0,2) == childIDmanual.ToUpper().Substring(1,2) &&
childIDverification1.ToUpper().Substring(2,3) == childIDmanual.ToUpper().Substring(4,3))
*/
childID.ToUpper().ToCharArray()[0]=='N' && /* must atrt with 'N' */
n>=0 &&
n<=32767 && /* 32768 IDs */
childID.ToUpper().ToCharArray()[3]==(int)chks /* 4th digit is checksum */
```

APPENDIX B — VARIABLES

[1] n:
(childID.ToUpper().ToCharArray()[1]-48-1)*10000 + (childID.ToUpper().ToCharArray()[2]-48)*1000 + (childID.ToUpper().ToCharArray()[4]-48)*100 + (childID.ToUpper().ToCharArray()[5]-48)*10 + (childID.ToUpper().ToCharArray()[6]-48)

[2] chks:
new int[]{ (int)checksum[(int)rc].d1, (int)checksum[(int)rc].d2, (int)checksum[(int)rc].d3, (int)checksum[(int)rc].d4, (int)checksum[(int)rc].d5, (int)checksum[(int)rc].d6, (int)checksum[(int)rc].d7, (int)checksum[(int)rc].d8, (int)checksum[(int)rc].d9, (int)checksum[(int)rc].d10 }[(int)c-1]

[3] tlix:
new int[]{ (int)tl[(int)rc].d1, (int)tl[(int)rc].d2, (int)tl[(int)rc].d3, (int)tl[(int)rc].d4, (int)tl[(int)rc].d5, (int)tl[(int)rc].d6, (int)tl[(int)rc].d7, (int)tl[(int)rc].d8, (int)tl[(int)rc].d9, (int)tl[(int)rc].d10 }[(int)c-1]

LEGEND

Legend and structure of information in this file

Name of section

Enabling condition for this section

Question title

Type of question, scope

Variable name

Answer options

SECTION 5: OTHER INCOME SOURCES

E `s4_other_sources_which.Contains(98)`

Duis aute irure dolor in reprehenderit in voluptate velit esse cillum dolore eu fugiat nulla pariatur?

MULTI-SELECT
SCOPE: PREFILLED

`s4_rel_leaders_other`

I This refers to family relations
E `s3_time_other > 0`
V1 `s4_rel_leaders_which.Contains(98)`
M1 Can not be itself
V2 `(s3_time_other_breeding_advice <= (50 - s3_time_art_in-sem_advice)) ||s3_time_other_breeding_advice == 0`
M2 This person is not in the list
F `optioncode != s5_ignored_option_code`

01 ☐ Community animal health workers
02 ☐ Private
03 ☐ Government
04 ☐ Livestock keepers association
05 ☐ NGO

And 5 other [13]

Additional information:
"I" — Question instruction
"E" — Enabling condition
"V1" — Validation condition №1
"M1" — Message for validation №1
"F" — Filter in Categorical questions

Link to full set in appendix

Breadcrumbs

Type or roster

Roster Title

CHAPTER 3 IDENTIFICATION /
Roster: LEADER RELATION DETAILS
generated by fixed list:

01 Ward Livestock Officer
02 Village Livestock Officer
99 Other (specify)

List items

Generated by mordorburkina, Sep 14, 2021 14:52
Questionnaire created by klaus, Sep 08, 2018 15:33
Last modified by mangoja8, Aug 18, 2021 11:17

Shared with:
wgodwin28 last edited 8/4/2020 5:27:27 PM
ying last edited 2/5/2019 7:31:12 PM
mordorburkina last edited 7/6/2021 10:13:29 PM
Alphonse1 (never edited)
mangoja8 last edited 8/18/2021 3:17:05 PM

Sections: 3, Sub-sections: 8, Questions: 56.
Questions with enabling conditions: 42
Questions with validation conditions:15
Rosters: 1
Variables: 8

Survey Solutions

4-NAITRE - Status Vital et Effets secondaires

SURVEY IDENTIFICATION INFORMATION
QUESTIONNAIRE DESCRIPTION

IDENTIFICATION
Sub-sections: 2, No rosters, Questions: 12, Variables: 6.

STATUT VITAL
No sub-sections, Rosters: 1, Questions: 19, Static texts: 4, Variables: 1.

EFFETS SECONDAIRES ET RISQUE DE SHPN
Sub-sections: 6, No rosters, Questions: 25, Static texts: 6, Variables: 1.

APPENDIX A — VARIABLES

LEGEND

SURVEY IDENTIFICATION INFORMATION
QUESTIONNAIRE DESCRIPTION

Basic information

Title 4-NAITRE - Status Vital et Effets secondaires

IDENTIFICATION

Tap to record the date	DATE: CURRENT TIME `startTime`
Tap to record GPS location	GPS `gpsLocation`<br>N<br>W<br>A
Child's first name	TEXT `childFirstname`
Child's last name	TEXT `childLastname`
VARIABLE<br>"blue"	STRING `colorName`
%childFirstname%'s ID	SINGLE-SELECT `childIDmode`<br>01 ○ Scan the code<br>02 ○ Enter the code manually
Scan %childFirstname%'s ID<br>E `childIDmode==1`<br>V1 `self.ToUpper().ToCharArray()[0]=='N' && /* must atrt with 'N' */ n>=0 && n<=32767 && /* 32768 IDs */ self.ToUpper().ToCharArray()[3]==(int)chks /* 4th digit is checksum */`<br>M1 Invalid ID	BARCODE `childIDbarcode`
Enter %childFirstname%'s ID<br>E `childIDmode==2`<br>V1 `self.ToUpper().ToCharArray()[0]=='N' && /* must atrt with 'N' */ n>=0 && n<=32767 && /* 32768 IDs */ self.ToUpper().ToCharArray()[3]==(int)chks /* 4th digit is checksum */`<br>M1 Invalid ID	TEXT `childIDmanual`

IDENTIFICATION
VERIFICATION
E `IsAnswered(childIDmanual) && false`

Enter the code again<br>V1 `self.ToUpper() == childIDmanual.ToUpper()`<br>M1 Not the same code ! - Please verify !	TEXT `childIDverificationX`

IDENTIFICATION
VERIFICATION
E `IsAnswered(childIDmanual)`

**Enter the letters in the code**<br>V1 `self.ToUpper().Substring(0,1) == childIDmanual.ToUpper().Substring(0,1) && self.ToUpper().Substring(1,1) == childIDmanual.ToUpper().Substring(3,1)`<br>M1 Not the same code ! - Please verify !	TEXT `childIDverification`<br>..
**Enter the numbers in the code**<br>V1 `self.ToUpper().Substring(0,2) == childIDmanual.ToUpper().Substring(1,2) && self.ToUpper().Substring(2,3) == childIDmanual.ToUpper().Substring(4,3)`<br>M1 Not the same code ! - Please verify !	TEXT `childIDverification1`<br>..
VARIABLE<br>`IsAnswered(childIDbarcode) ? childIDbarcode : childIDmanual`	STRING `childID`
VARIABLE<br>`(childID.ToUpper().ToCharArray()[1]-48-1)*10000 + (childID.ToUpper().ToCharArray()[2]-48)*1000 + (childID.ToUpper().ToCharArray()[4]-48)*100 + (childID.ToUpper().ToCharArray()[5]-48)*10 + (child`<br>And 33 other symbols [1]	LONG `n`
VARIABLE<br>`n/10+1`	LONG `rc`
VARIABLE<br>`n%10+1`	LONG `c`
VARIABLE<br>`new int[]{ (int)checksum[(int)rc].d1, (int)checksum[(int)rc].d2, (int)checksum[(int)rc].d3, (int)checksum[(int)rc].d4, (int)checksum[(int)rc].d5, (int)checksum[(int)rc].d6,`<br>And 133 other symbols [2]	LONG `chks`
**What is %childFirstname%'s birthdate**<br>E `$childIDverified`<br>V1 `self.Value.AddDays(14) < startTime`<br>M1 Please check on birthdate!	DATE `childBirthdate`<br>..
**What is %childFirstname%'s sex**<br>E `IsAnswered(childBirthdate)`	SINGLE-SELECT `childSex`<br>01 ○ Masculin<br>02 ○ Féminin

STATUT VITAL

E $childIDverified

Which follow up visit?<br>V1 self == 365<br>M1 Only Day 365 follow up is allowed. All other timepoints are out of windo w.	SINGLE-SELECT timepoint<br>010 ◯ Visit 1<br>090 ◯ Day 90<br>180 ◯ Day 180<br>365 ◯ Day 365

STATIC TEXT

E (timepoint==180 && childBirthdate.Value.AddDays(222) < startTime) \|\| (timepoint==180 && childBirthdate.Value.AddDays (138) > startTime)

Are you sure you entered correct timepoint?

STATIC TEXT

E (timepoint==365 && childBirthdate.Value.AddDays(407) < startTime) \|\| (timepoint==365 && childBirthdate.Value.AddDays (323) > startTime)

Are you sure you entered correct timepoint?

How are you conducting the follow-up visit?<br>E timepoint == 7 || timepoint == 14 || timepoint == 21 || timepoint == 90 || timepoint == 365 || timepoint == 10 || timepoint == 180	SINGLE-SELECT visitLocation<br>01 ◯ Home visit<br>02 ◯ On phone<br>03 ◯ Autre
Où est-ce ?<br>E visitLocation==3	SINGLE-SELECT visitLocationautre<br>01 ◯ Marché<br>02 ◯ CSPS<br>03 ◯ Autre
%childFirstname%'s status	SINGLE-SELECT vitalStatus<br>01 ◯ Alive<br>02 ◯ Died<br>04 ◯ Unknown
When did %childFirstname% die?<br>E vitalStatus == 2<br>V1 self < startTime<br>M1 This cannot be death date, it is in future	DATE deathDate<br>..
When can we come back to do verbal autopsy report?<br>E vitalStatus == 2<br>V1 self > startTime<br>M1 Please check that date is in future	DATE autopsyDate<br>..
Can you take the anthropometric measurements?<br>E timepoint==180 && $childAlive	SINGLE-SELECT anthroQ<br>01 ◯ Yes<br>02 ◯ No
Why not?<br>E anthroQ==2	TEXT anthroNo<br>..

STATUT VITAL

Roster: LENGTH MEASUREMENTS

generated by fixed list R_length

01 1

02 2

03 3

E `timepoint == 180 && $childAlive && anthroQ == 1`

%childFirstname%'s length measurement (cm)	NUMERIC: DECIMAL length_m
V1 `self.InRange(30,80)`<br>M1 This can't be the length of an infant!<br>V2 `@rowcode==2?self.InRange(R_length[1].length_m*0.9,R_length[1].length_m*1.1):true`<br>M2 The two measurements are very different! Please verify!<br>V3 `@rowcode==3?self.InRange(R_length[1].length_m*0.9,R_length[1].length_m*1.1) && self.InRange(R_length[2].length_m*0.9,R_length[2].length_m*1.1):true`<br>M3 The 3 measurements differ too much. Please verify!	- -
VARIABLE<br>`R_length.Sum(x => (int)x.length_m) / 3`	LONG length

STATIC TEXT

E `R_length.Count(x => IsAnswered(x.length_m)) == 3`

Length (average) = %length%

Notes?	SINGLE-SELECT hasLengthNotes
E `length > 0 && $childAlive`	01 ○ Yes<br>02 ○ No
Enter your notes<br>E `hasLengthNotes==1`	TEXT Lengthnotes<br>..
%childFirstname%'s weight<br>E `timepoint == 180 && $childAlive && anthroQ==1`<br>V1 `self.InRange(1,20)`<br>M1 This can't be the weight in lbs or kg!	NUMERIC: DECIMAL weight<br>- -
Which units?<br>E `IsAnswered(weight)`	SINGLE-SELECT weightUnit<br>01 ○ kg<br>02 ○ lbs
Notes?<br>E `weight > 0 && $childAlive`	SINGLE-SELECT hasWeightNotes<br>01 ○ Yes<br>02 ○ No
Enter your notes<br>E `hasWeightNotes==1`	TEXT weightnotes<br>..
MUAC (cm)<br>E `$childAlive && timepoint == 180 && anthroQ==1`<br>V1 `self>0 && self<20`<br>M1 This can't be the MUAC in cm!	NUMERIC: DECIMAL MUAC<br>- -

STATIC TEXT

E `MUAC < 11.5`

Refer %childFirstname% to the clinic!

Sign of malnutrition<br>E `$childAlive && timepoint == 180 && anthroQ==1`	MULTI-SELECT: YES/NO — `malnutrition`<br>01 ☐ / ☐ Pedal oedema<br>02 ☐ / ☐ Kwashiorkor<br>03 ☐ / ☐ Marasmus
Notes<br>E `$childAlive && timepoint == 180`	SINGLE-SELECT — `hasMalnutritionNotes`<br>01 ○ Yes<br>02 ○ No
Enter your notes<br>E `hasMalnutritionNotes==1`	TEXT — `malnutritionNotes`<br>..

EFFETS SECONDAIRES ET RISQUE DE SHPN

E `$childAlive`

Since the last time our team visited, has %childFirstname% presented any following symptoms?<br><br>E `$aeTimePoint`	MULTI-SELECT — q_5_6<br>01 ☐ Fever<br>02 ☐ Diarrhea<br>03 ☐ Vomiting<br>04 ☐ Abdominal pain<br>05 ☐ Rash<br>06 ☐ Constipation<br>07 ☐ Aucun
Vomit after every feed?<br><br>E `$aeTimePoint && q_5_6.Contains(3)`	SINGLE-SELECT — vomitAfterfeeding<br>01 ○ Yes<br>02 ○ No
How long has this been going on?<br><br>E `vomitAfterfeeding == 1`	SINGLE-SELECT — regvomitDuration<br>01 ○ Less than 2 days<br>02 ○ 2 days or longer
Is this issue still happening in past 24 hours?<br><br>E `vomitAfterfeeding == 1`	SINGLE-SELECT — regvomitStatus<br>01 ○ Yes<br>02 ○ No
Is child able to hold any food down?<br><br>E `$aeTimePoint && q_5_6.Contains(3)`	SINGLE-SELECT — holdFoodDown<br>01 ○ Yes<br>02 ○ No
How long has this been going on?<br><br>E `holdFoodDown == 2`	SINGLE-SELECT — holdFoodDownDur<br>01 ○ Less than 2 days<br>02 ○ 2 days or longer
Is this issue still happening in past 24 hours?<br><br>E `holdFoodDown == 2`	SINGLE-SELECT — holdFoodDownStatus<br>01 ○ Yes<br>02 ○ No

STATIC TEXT

E `(holdFoodDownStatus == 1 && holdFoodDownDur == 2) || (regvomitStatus == 1 && regvomitDuration == 2)`

Refer %childFirstname% immediately to physician!

VARIABLE<br>`startTime.Value.AddDays(3).ToString("dd-MM-yyyy")`	STRING — followup3

EFFETS SECONDAIRES ET RISQUE DE SHPN

FOLLOW-UP DATE

E `(holdFoodDownStatus == 1 && holdFoodDownDur == 1) || (regvomitStatus == 1 && regvomitDuration == 1)`

STATIC TEXT

Follow up with %childFirstname% in 3 days on %followup3%

Has %childFirstname% projectile vomitted?<br><br>E `$aeTimePoint && q_5_6.Contains(3)`	SINGLE-SELECT — projVomit<br>01 ○ Yes<br>02 ○ No

EFFETS SECONDAIRES ET RISQUE DE SHPN
IHPS

E `projVomit==1`

Projectile vomit after every feed?	SINGLE-SELECT `afterFeeding`<br>01 ○ Yes<br>02 ○ No
How long has this been going on?<br>E `afterFeeding == 1`	SINGLE-SELECT `vomitDuration`<br>01 ○ Less than 2 days<br>02 ○ 2 days or longer
Is this issue still happening in past 24 hours?<br>E `afterFeeding == 1`	SINGLE-SELECT `projVomitStatus`<br>01 ○ Yes<br>02 ○ No

EFFETS SECONDAIRES ET RISQUE DE SHPN / IHPS
FOLLOW-UP DATE

E `vomitDuration == 1 && projVomitStatus == 1`

STATIC TEXT

Follow up with %childFirstname% in 3 days on %followup3%

EFFETS SECONDAIRES ET RISQUE DE SHPN / IHPS
CONSULTATION REQUEST

E `vomitDuration == 2 && projVomitStatus == 1`

STATIC TEXT

Please refer %childFirstname% to the district hospital!

Has %childFirstname% peed at least 3 times in past 24 hours?<br>E `projVomit == 2 || afterFeeding == 2`	SINGLE-SELECT `urineFreq`<br>01 ○ Yes<br>02 ○ No

EFFETS SECONDAIRES ET RISQUE DE SHPN
HYDRATION WARNING

E `urineFreq == 1`

STATIC TEXT

Instruct mother to make sure to keep %childFirstname% hydrated!

EFFETS SECONDAIRES ET RISQUE DE SHPN
CSPS REQUEST

E `urineFreq == 2`

STATIC TEXT

%childFirstname% may be dehydrated, instruct mother to go to CSPS for a checkup

Did you give mother a referral form?<br>E `urineFreq == 2`	SINGLE-SELECT `referForm`<br>01 ○ Yes<br>02 ○ No

Question	Response	Variable
Why not?<br>E referForm == 2	TEXT<br>..	noRefer
Did you contact Dr. Bountogo or the CRSN team?<br>E urineFreq == 2<br>V1 self == 1<br>M1 Please do so now before continuing with interview!	SINGLE-SELECT<br>01 ○ Yes<br>02 ○ No	yesRefer
Has %childFirstname% visited the health center since the last time we visited for a health problem?	SINGLE-SELECT<br>01 ○ Yes<br>02 ○ No<br>03 ○ Don't know	centerVisit
Number of times %childFirstname% visited the health center?<br>E centerVisit == 1	SINGLE-SELECT<br>01 ○ Enter number<br>02 ○ Don't know	centerNumVisit
Number of visits<br>E centerNumVisit == 1<br>W1 self.InRange(0,20)<br>M1 Please check this!	NUMERIC: INTEGER<br>- -	numCenVisits
Why did %childFirstname% visit the health center?<br>E centerVisit == 1	MULTI-SELECT<br>01 ☐ Fever<br>02 ☐ Diarrhea<br>03 ☐ Pneumonia<br>04 ☐ Malaria<br>05 ☐ Other	reasonVisit
Please specify reason for visit<br>E reasonVisit.Contains(5)	TEXT<br>..	specifyVisit
Has %childFirstname% been hospitalized since the last time we visited?	SINGLE-SELECT<br>01 ○ Yes<br>02 ○ No<br>03 ○ Don't know	hospitalVisit
Number of times %childFirstname% visited the hospital?<br>E hospitalVisit == 1	SINGLE-SELECT<br>01 ○ Enter number<br>02 ○ Don't know	hospitalNumVisit
Number of visits<br>E hospitalNumVisit == 1<br>W1 self.InRange(0,20)<br>M1 Please check this!	NUMERIC: INTEGER<br>- -	numHosVisits
Why was %childFirstname% hospitalized?<br>E hospitalVisit == 1	MULTI-SELECT<br>01 ☐ Fever<br>02 ☐ Diarrhea<br>03 ☐ Pneumonia<br>04 ☐ Malaria<br>05 ☐ Other	reasonHosVisit

Please specify reason for hospitalization<br>E reasonHosVisit.Contains(5)	TEXT specifyHosVisit<br>..

APPENDIX A — VARIABLES

[1] n:

(childID.ToUpper().ToCharArray()[1]-48-1)*10000 + (childID.ToUpper().ToCharArray()[2]-48)*1000 + (childID.ToUpper().ToCharArray()[4]-48)*100 + (childID.ToUpper().ToCharArray()[5]-48)*10 + (childID.ToUpper().ToCharArray()[6]-48)

[2] chks:

new int[]{ (int)checksum[(int)rc].d1, (int)checksum[(int)rc].d2, (int)checksum[(int)rc].d3, (int)checksum[(int)rc].d4, (int)checksum[(int)rc].d5, (int)checksum[(int)rc].d6, (int)checksum[(int)rc].d7, (int)checksum[(int)rc].d8, (int)checksum[(int)rc].d9, (int)checksum[(int)rc].d10 }[(int)c-1]

LEGEND

Legend and structure of information in this file

Name of section

Enabling condition for this section

Question title

Type of question, scope

Variable name

Answer options

SECTION 5: OTHER INCOME SOURCES

E s4_other_sources_which.Contains(98)

Duis aute irure dolor in reprehenderit in voluptate velit esse cillum dolore eu fugiat nulla pariatur?

I This refers to family relations
E s3_time_other > 0
V1 s4_rel_leaders_which.Contains(98)
M1 Can not be itself
V2 (s3_time_other_breeding_advice <= (50 - s3_time_art_insem_advice)) ||s3_time_other_breeding_advice == 0
M2 This person is not in the list
F optioncode != s5_ignored_option_code

MULTI-SELECT
SCOPE: PREFILLED

s4_rel_leaders_other

01 ☐ Community animal health workers
02 ☐ Private
03 ☐ Government
04 ☐ Livestock keepers association
05 ☐ NGO

And 5 other [13]

Additional information:
"I" — Question instruction
"E" — Enabling condition
"V1" — Validation condition №1
"M1" — Message for validation №1
"F" — Filter in Categorical questions

Link to full set in appendix

Breadcrumbs

Type or roster

Roster Title

CHAPTER 3 IDENTIFICATION /
Roster: LEADER RELATION DETAILS
generated by fixed list:

01 Ward Livestock Officer
02 Village Livestock Officer
99 Other (specify)

List items

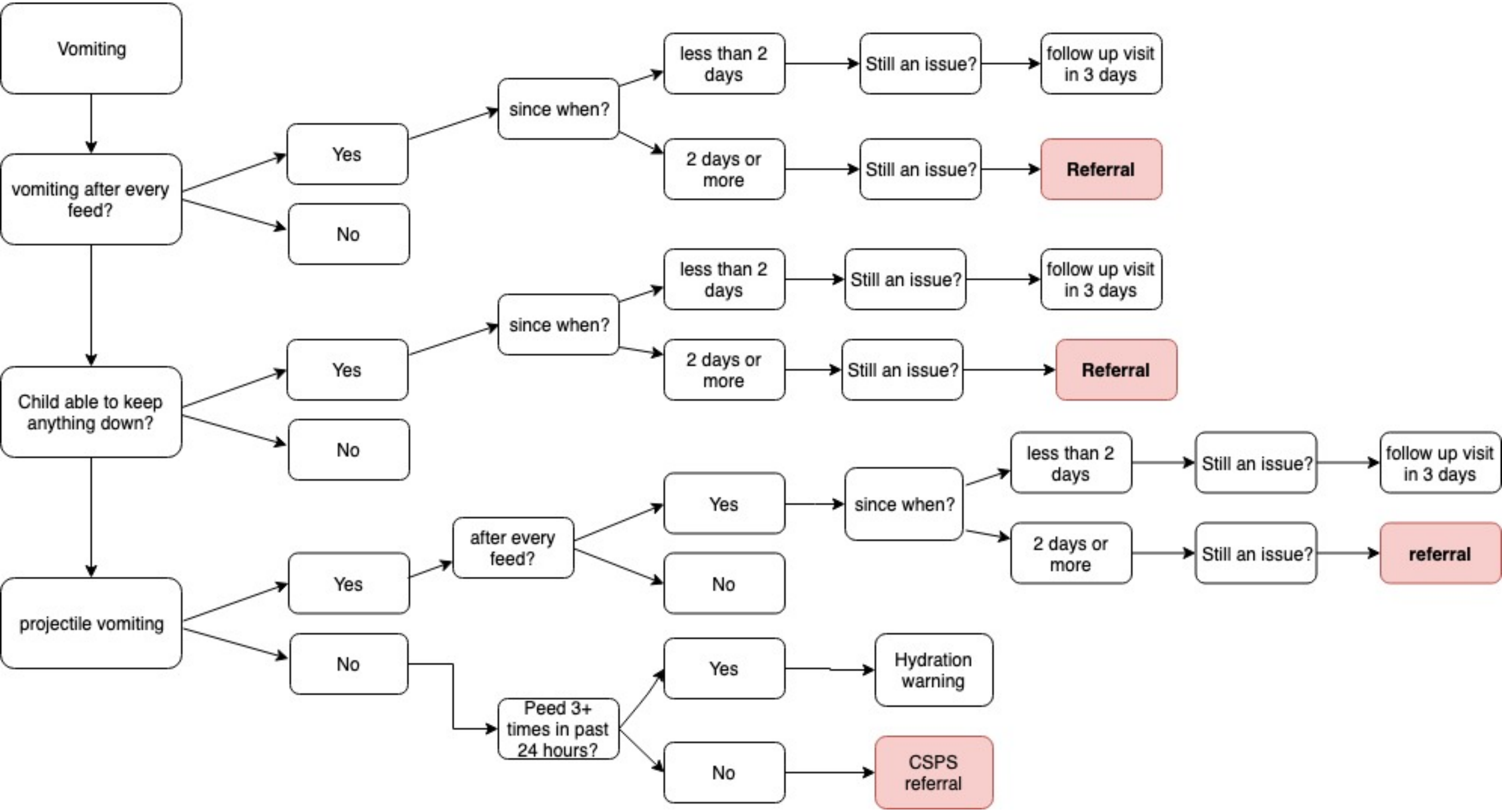
Vomiting
vomiting after every feed?
Yes
No
since when?
less than 2 days
Still an issue?
follow up visit in 3 days
2 days or more
Still an issue?
Referral
Child able to keep anything down?
Yes
No
since when?
less than 2 days
Still an issue?
follow up visit in 3 days
2 days or more
Still an issue?
Referral
projectile vomiting
Yes
after every feed?
Yes
since when?
less than 2 days
Still an issue?
follow up visit in 3 days
2 days or more
Still an issue?
referral
No
No
Peed 3+ times in past 24 hours?
Yes
Hydration warning
No
CSPS referral

Si vous avez des questions ou des inquiétudes concernant la SHPN, veuillez contacter le Dr. Ali Sié par email à

alisie.crsn@fasonet.bf

ou par téléphone au

20537043

ou Dr. Mamadou Bountogo par téléphone au +22670398944 et par email à drbountogo@yahoo.fr

University of California San Francisco

STENOSE HYPERTROPHIQUE DU PYLORE DU NOURRISON (SHPN)

<u>Qu'est ce que le SHPN?</u> C'est une maladie rare qui empêche les aliments d'entrer dans l'intestin grêle. Cette maladie se produit quand le muscle du pylore s'épaissit et devient gros entraînant un blocage de la nourriture dans l'estomac.

<u>Qu'est ce qui cause le SHPN?</u> Les causes de cette maladie sont inconnues mais certaines recherches suggèrent que l'utilisation d'antibiotiques dans les prèmieres semaines de vie augmentent les chances de SHPN. Le chirurgie est necessaire pour soigner la SHPN.

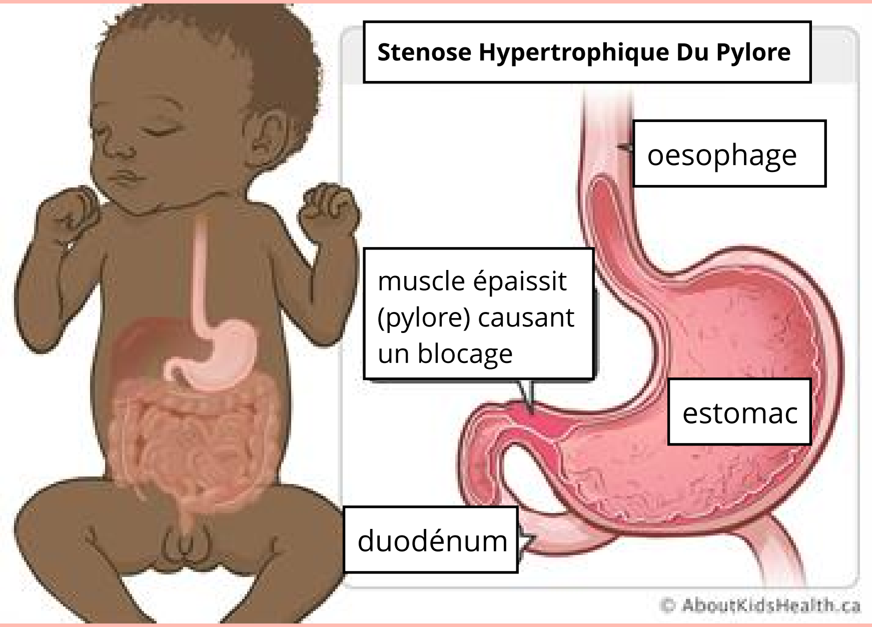

Symptômes de la SHPN

Vomissement en jets:

Votre bébé peut vomir avec force (en jet) après avoir mangé. Le vomissement peut contenir du sang. Les vomissements peuvent être légers au début mais deviennent progressivement plus importants.

Sensation de faim persistante:

Les bébés atteints de SHPN ont faim constamment et veulent manger après avoir vomi.

Contractions de l'estomac:

Vous pouvez remarquer des contractions de l'estomac au niveau de l'abdomen de votre bébé tout de suite après le repas mais avant que votre bébé ne se mette à vomir.

Perte de poids:

Les bébés atteints de SHPN peuvent avoir des problémes pour grossir ou peuvent perdre du poids.

Changement dans le nombre de selles:

Comme la SHPN empeche la nourriture d'atteindre l'appareil digestif, les bébés atteints de SHPN ont souvent moins de selles et peuvent etre constipés.

Déshydratation:

Votre enfant peut pleurer sans qu'aucune larme n'apparaisse ou votre enfant peut uriner moins de 3 fois par jour.

Diagnostic

La SHPN est diagnostiquée par échographie afin de voir le muscle du pylore et confirmer la présence d'une sténose.

Traitement

La chirurgie est nécessaire pour soigner la SHPN.

Si vous pensez que votre enfant est atteint de SHPN

Contactez un membre de notre équipe immédiatement si votre enfant présente des signes de SHPN. Le traitement pour cette maladie ne sera pas à votre charge. Cette maladie est rare mais nous surveillerons votre enfant attentivement pour l'apparition de ces symptômes.

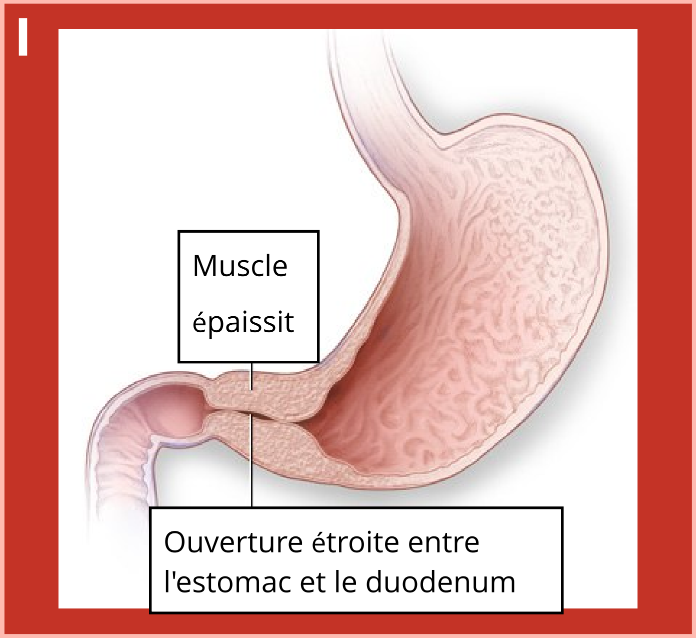

Appendix 5: Anthropometry Protocol

Anthropometric measurements will be collected at baseline and at the 6-month visit, including length, weight, and mid-upper arm circumference. We will collect three consecutive measurements for length at both visits. The child will also be evaluated for signs of malnutrition.

Material
Tablet and accessories
Child’s carnet de santé and unique study ID
ADE electronic baby scale
Shorrboard
MUAC tape
Hand sanitizer
Disinfecting wipes
Pen and marker
Trash bag
Scale batteries

Length Measurement
Length will be measured to the nearest centimeter using a portable ShorrBoard.

It is important to remove large hair ornaments or hats that could falsify the height measurement. Additionally, it is important to remove shoes, sandals, and socks.

If the measurement of the child's height requires the presence of the guardian to reassure the child, explain the purpose of the measurement and describe the different steps of the procedure. Answer any questions he/she may have. Show and explain how he/she can be helpful. Explain that it is important for the child to remain calm and still to get a good measurement.

<u>ShorrBoard Set-Up</u>

1. Remove the board from the bag.
2. Place the board flat against table. When you are facing the board, turn the bolt counterclockwise to release the extension. Note : The bolt should remain attached to the back of the extension piece. Do not remove it.
3. Slide the extension piece to the top of the main board and attach the clasp to the back of the board. Make sure the clasp is secure.
4. The footrest and self-locking headrest are stored at the base of the main board and can be moved up and down along the measuring board. They should lock and stay in place regardless of the their position.

5. The measuring board must be placed on a firm surface. Clean the board with a disinfecting wipe at the beginning of each day and after each child.
6. Note : When you set up the board each day, make sure each part is in good condition and not damaged/broken.

Measuring the child

1. Place an absorbent pad on the ShorrBoard for hygiene and the baby's comfort. Explain to the caregiver that she should place her child on the board and hold the child's head in place during the measurement. Show the caregiver where to place their hands at the top of the child's head and against the headrest.
2. When the caregiver understands the instructions and is ready to assist, ask the caregiver to place the child on the board with their back against the board and the head against the fixed headrest while gently compressing the hair.
3. Quickly position the child's head perpendicular to the board, imagining a vertical line from the ear canal to the lower edge of the eye (the child's eyes should look up). Ask the mother to stand behind the headrest and hold the child's head in this position.
4. Speed is important. Stand on the side of the board where you can see the measuring markers on the board and move the footrest.
5. Ensure the child is lying straight along the board and the spine should not be arched. Ask the mother to tell you if the child's back arches or changes position. Hold the child's legs with one hand and move the footrest with the other hand. Apply gentle pressure on the knees to stretch the legs as far as they can go without causing pain or injury. **Note :** It is not possible to strech a newborn's knees in the same way as an older child. Their knees are fragile and can be easily injured. Therefore, apply minimum pressure.
6. If the child is agitated and the two legs cannot be mainted in position, measure the child with a single leg in position. While holding the knees, push the footrest against the child's foot.
7. Repeat the measurement three separate times. After each measurement, record it in the tablet. Note the child's lenght in centimeters, rounding down to the nearest millimeter (e.g. 87.25 cm should be recorded as 87.2 cm). Keep the child in place, release the footrest and prepare to replace the child for a second and third measurement.

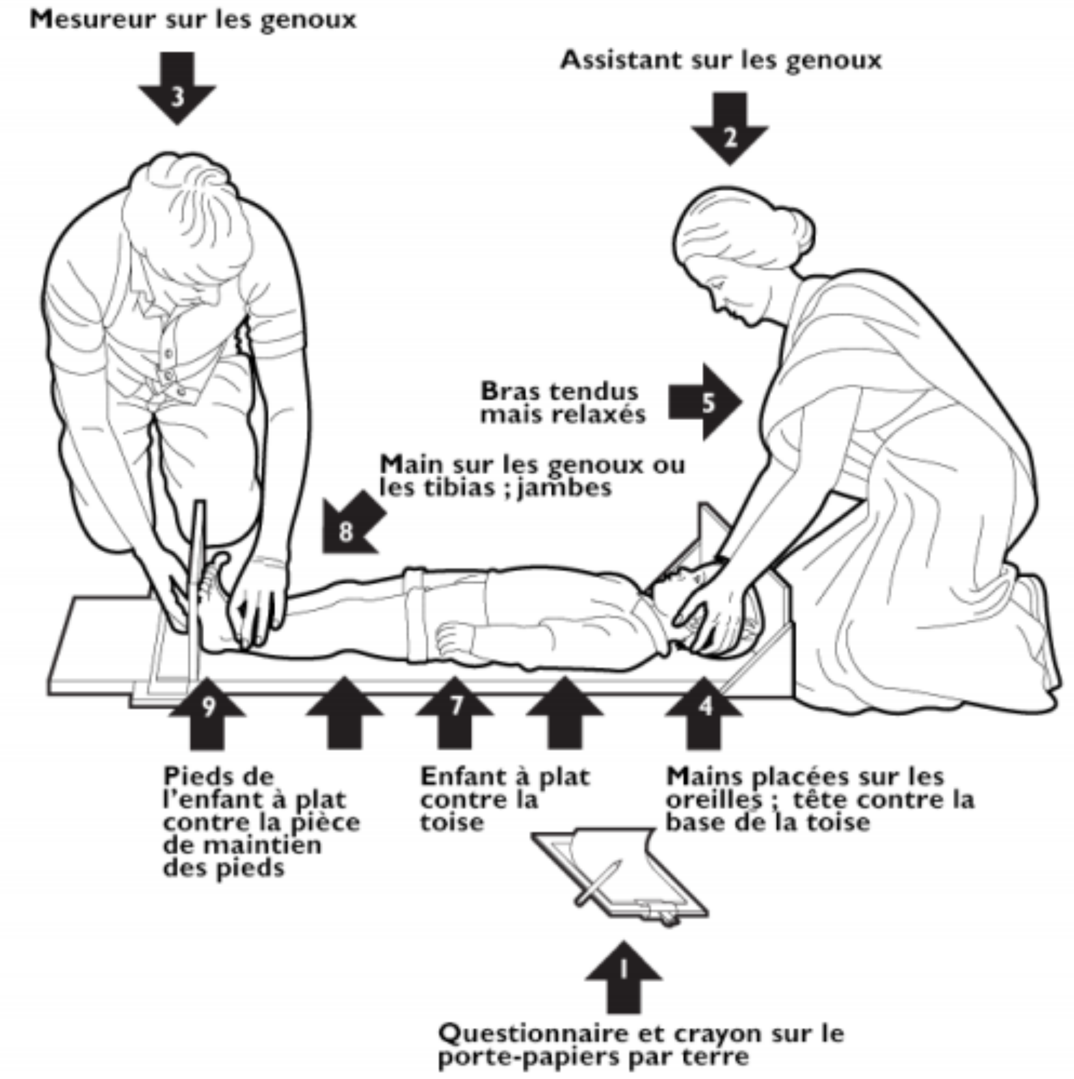
Mesureur sur les genoux
3
Assistant sur les genoux
2
Bras tendus
mais relaxés
5
Main sur les genoux ou
les tibias ; jambes
8
9
7
4
Pieds de
l'enfant à plat
contre la pièce
de maintien
des pieds
Enfant à plat
contre la
toise
Mains placées sur les
oreilles ; tête contre la
base de la toise
1
Questionnaire et crayon sur le
porte-papiers par terre

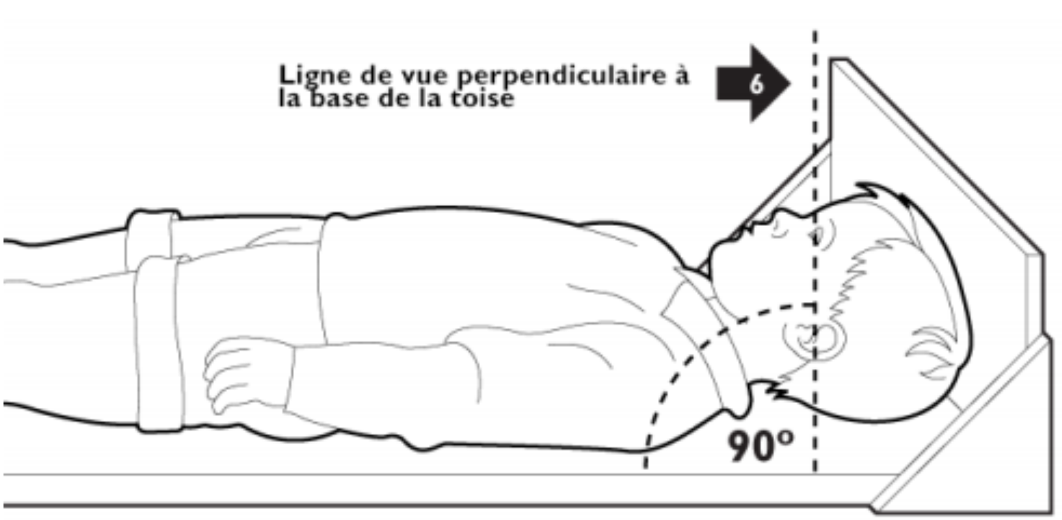
Ligne de vue perpendiculaire à
la base de la toise
6
90°

Disassembling the portable measuring board

1. Hold the board upright. Stand in front of the board and place on foot on the base to keep it stable.
2. Slide the headrest/footrest onto the base of the main board.
3. Unclip the clasp on the back of the extension piece. Push the clasp flat against the extension board.
4. To attach the extension piece to the main board, turn the front of the extension piece inward and place it against the front of the main board. Make sure all sides of the extension piece are aligned with the main board.
5. Push in the bolt on the back of the extension board and attach it to the main board.
6. Place the tower in the carrying bag until the next time you use it.

Weight Measurement

We are using the ADE M112600U scale to measure the child in kilograms.

Standardize the scale

Each morning before using the scale, you must make sure that the scale is functional.

1. Place the scale on a stable, firm, and flat surface.
2. Make sure the 4 legs of the scale are stable. The 4 legs should touch the flat surface.
3. Turn on the scale and make sure the numbers « 0.000 » appear.
4. We will do two tests :
   a. Test 1 : We will weigh a test weight.
      i. Place the 2 kg test weight on the scale.
      ii. Note the number appearing on the scale on the test weight form in column 1. Remove the test weight and wait for the scale to return to « 0.000 ».
   b. Test 2 :
      i. Place books or another item on the scale until it weighs 3 kg.
      ii. Add the test weight
      iii. Calculate the difference in weights (Weight with books alone subtracted from weight with books and test weight).
      iv. Remove all weight from the scale.
      v. The values you will find during these tests should be around 2kg. However, all scales have a margin of error that is considered normal. If the weights weighed are between 1.8kg - 2.2kg this is normal. Please contact Dr. BOUNTOGO if the values are below or above this margin of error.

Setting up the ADE M112600U scale

1. Remove the scale from the bag.
2. Ensure the scale is placed on a firm, flat surface. The four corners of the scale should be touching the flat surface without moving. This is very important for the child's safety and the precision of the measurement.

3. The scale turns off automatically after 5 minutes of inactivity. If you wish to turn it on manually, push the on button for 3 seconds until the scale lights up.
4. The scale has a capacity of 20 kg. If the screen shos « o.Ld », this signifies that there is too much weight on the scale.
5. If the letters « Lo » appear on the screen, this signifies that you must change the batteries. Remove the old batteries and replace them with 4 new ones.
6. Never leave the child on the scale alone without surveillance !
7. Place the scale back in the bag at the end of the day.

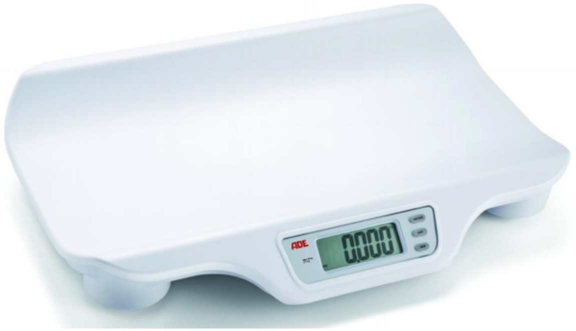

Weighing the child

1. Explain to the caregiver that we would like to weigh child in order to evaluate the child's growth. The child should not wear heavy clothing, shoes, sandals, hair ornaments, or jewelery. Explain that the child must remove all of this for an accurate measurement. If the child is wearing a diaper, it should be removed.
2. When the scale is properly in place and turned on, place the chld on the scale. The numbers on the scale will change for a few seconds until the weight stabilizes. When the weight is stable, the display will flash once and the weight will appear. The child's weight will reamin on the display for 5 minutes or until you remove the child from the scale.
3. Record the the weight in the tablet and ask the mother to take the child back. You should take 3 seperate measurements. **Note :** If the child does not stop moving, your number will not freeze. Try to tell the mom to talk to the child during the procedure to calm the child, but do not touch them.

Measure the mid-upper arm circumference (MUAC)

We will measure the middle of the child's left arm between the point of the shoulder and the point of the elbow using a soft but not stretchy tape measure.

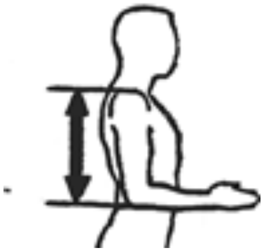

1. First, find the middle of the child's arm. Place the child standing with feet together, arm alongside the body bent at 90 degrees palm up.
2. Place the end of a string on the point of the shoulder.
3. Place the other end of the string on the point of the elbow.
4. Fold the string in half, bringing the bottom part over the top part.
5. Mark the middle with the marker.
6. Stand facing the child on the left side. The child should be standing upright, straight, with the arms at the side of the body.
7. Place the flexible tape around the child's arm at the marked point.
8. Pull the tape until it is well placed with contact on the skin. Don't pull too tight, as the skin should not compress.
9. The place where you should read the measurement is clearly marked on the tape with two large arrows. Read the number that is aligned there. After reading the measurements, record them in the tablet to the nearest millimeter.
10. Remove the tape. If it is the 6 month visit and the circumference is less than 11.5 cm, refer the child to the health center for care.

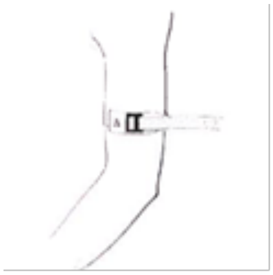

Signs of malnutrition

The examiner will observe the participant for signs of malnourishment:

1. **Kwashiorkor:** look for edema (or swelling); thin, sparse, or discolored hair; and skin with discolored patches that may crack and peel.
2. **Marasmus:** look for severe wasting; the appearance of 'skin and bones;' and a face that looks like an old man's.
3. **Pedal edema:** look for swelling due to excess fluid in the foot. Press the child's foot with your thumb. If the foot is swollen and the indentation remains after you press it, the child has edema.

If the child exhibits any signs of malnutrition, refer them to the health center for care. Mark any signs of malnutrition in the tablet.
