# Supplementary material for: Infant mortality and growth failure after oral azithromycin among low birthweight and underweight neonates: A subgroup analysis of a randomized controlled trial
Source: PLOS Glob Public Health. 2023 May 15;3(5):e0001009. doi: 10.1371/journal.pgph.0001009 (PMC10184901; doi:10.1371/journal.pgph.0001009)
Supplement: S2 File — (PDF) [file pgph.0001009.s003.pdf]

NAITRE

MORDOR Project

June 10, 2021

Version 2.1

Statistical Analysis Plan

UCSF Francis I. Proctor Foundation
Centre de Recherche en Sante de Nouna University of Heidelberg

Introduction

This document (Statistical Analysis Plan, SAP) describes the planned analysis and reporting for the clinical trial, **Azithromycin for the prevention of neonatal mortality in Burkina Faso: an individual-randomized trial**. It includes specifications for the statistical analyses and tables to be prepared for the interim and final Clinical Study Report. This study is a Phase IV clinical trial to compare methods to reduce childhood mortality using mass administration of azithromycin (Pfizer, CAS 83905-01-5) compared to placebo. The content of this Statistical Analysis Plan meets the requirements stated by the US Food and Drug Administration and conforms to the American Statistical Association's Ethical Guidelines.

The following documents were reviewed in preparation of this Statistical Analysis Plan:

- Azithromycin for the prevention of neonatal mortality in Burkina Faso: an individual-randomized trial, Manual of Operations
- Azithromycin for the Prevention of Neonatal, Infant, and Child Mortality in Burkina Faso and Safety Assessment, Proposal
- Statistical Analysis Plan, MORDOR Trial
- ICH Guidance on Statistical Principles for Clinical Trials

The planned analyses described in this SAP will be included in future manuscripts. Exploratory analyses not necessarily identified in this Statistical Analysis Plan may be performed to support the analysis. Unplanned analyses not delineated in this Statistical Analysis Plan will be documented as such in the final Clinical Study Report and manuscripts. Note that unplanned analyses will be broadly grouped into two categories:

1. Sensitivity analyses for pre-specified outcomes
2. Other unspecified analyses

Sensitivity analyses are defined as alternate ways of modeling the primary outcome to ensure the finding was not dependent on the analytic plan, and these will not be subject to a multiple comparisons correction. Other unspecified analyses will be declared hypothesis generating or subject to an alpha level of 0.001.

This document will be reviewed prior to the enrollment of patients. All subsequent changes will be indicated by detailed change log in the Appendix.

Contents

1 **Summary** **5**

1.1 Mortality Trial 5

2 **Statistical Analysis** **5**

2.1 Planned Analyses 5

2.1.1 Mortality Trial 5

2.2 Interim Monitoring 9

3 **Sample Size Considerations** **10**

4 **Randomization** **12**

5 **Abbreviations** **13**

6 **Revision History** **15**

1 Summary

1.1 Mortality Trial

The trial profile is given in the Manual of Operations. In the mortality trial, infants are randomized to two arms: **Azithro** and **Control**. The trial is a placebo-controlled, double-masked (i.e., double-blind), individual-randomized clinical trial.

Objective. Establish the safety and efficacy of a single dose of azithromycin among neonates. Neonates age 8 to 27 days will be randomized to a single dose of azithromycin or placebo. *We hypothesize that neonates randomized to a single dose of azithromycin will have significantly lower all-cause mortality by 6 months of age, compared to those randomized to placebo.*

2 Statistical Analysis

2.1 Planned Analyses

2.1.1 Mortality Trial

Children between the ages of 8 and 27 days may be enrolled in the trial and be offered placebo or azithromycin in a masked fashion. Any child known to be living at a six month follow-up visit is counted as alive, any child known to have died is counted as dead, and any other child is counted as missing or lost to follow-up. Children followed up within six weeks of the actual six month follow-up date will be included in primary analysis. Deaths that occur on the day of enrollment after randomization will be assigned 0.5 days of person-time. Deaths that occur between two follow-up timepoints will be assigned person-time in accordance to the midpoint between the two follow-up dates (i.e. a death occurring between the 3-month and 6-month timepoint will contribute 1.5 months of additional person-time). As a sensitivity analysis, children that were followed up within twelve weeks of six month follow-up date will be analyzed.

Primary Analysis.

The primary analysis will be conducted as **binomial regression** using complementary log-log link. Firth correction will be employed to ensure numerical stability. Inference will be based on the permutation test.

The analysis will be **two-sided**, with a **type I error rate** (alpha) of 0.05.

Statistical considerations.

- Statistical tests will be conducted with Monte Carlo permutation based on the randomization unit. The number of replications will be 10,000, unless the Monte Carlo confidence interval for the P-value includes 0.05. In this case, 10,000,000 will be used, and this fact reported.
- Model adequacy will be checked by examination of residuals or other goodness of fit tests as needed. Inadequate model fit will prompt us to report alternative models.
- Multiple imputation will be used in case of missing baseline covariates (if applicable). Missing outcome variables will be handled by sensitivity analysis and reporting of conditional results.

Supplementary analyses.

The purpose of the supplementary analyses reported in this section is to assess the role of statistical choices and data quality choices in shaping the result.

mortality Fisher exact test

Secondary analyses of mortality.

All secondary analyses will be sharply distinguished from the primary prespecified analysis and will be identified as such. Secondary analyses include outcome variables or planned subsets which contribute either additional insight or address different scientific questions than the primary analysis.

A pre-specified secondary analysis will pool infants randomized in the neonate and individual studies using a binomial regression with complementary log-log link, permuted within study. A pooled sample size of 54414 infants would have 80% power to detect a 12% reduction in mortality.

age Binomial regression with complementary log-log link, using age at treatment (in days)

Prespecified subgroup analyses.

We will estimate differences separately by the following subgroups, defined by baseline characteristics, for both mortality and anthropometry endpoints:

- **age** Age at enrollment, by week
- **sex** Male versus female
- **season of enrollment** Rainy versus dry
- **region** Centre, Boucle du Mouhoun, Cascade, Centre Ouest, Haut-Bassins
- **urbanicity** Urban versus peri-urban versus rural
- **birthweight** Low birthweight (<2500 g) versus normal birthweight (>= 2500 g)
- **Underweight (WAZ < –2), stunted (LAZ < –2), wasted (WLZ < –2)**

Additional secondary outcomes.

Note that participants outside of WHO Child Growth Standards for WAZ (-6 to +5 SD), HAZ (-6 to +6 SD), or WHZ (-5 to +5 SD) will be excluded from all anthropometric analyses.

infantile hypertrophic pyloric stenosis Since the importance of avoiding type II errors in safety studies is well known [SL12], we propose to report the one-sided 90% confidence interval of the estimated relative risk for IHPS, and tabulate the occurrence of IHPS by age at treatment and gender.

adverse events The total number of individuals reporting an adverse event, the number reporting each adverse event; composite serious adverse events. Adverse events reported on any of the 7, 14, and 21-day timepoints will be reported in aggregate.

clinic visit Negative binomial regression on clinic visit counts, for the following reasons given for visiting the clinic: Malaria, Pneumonia, Diarrhea, Fever.

hospitalization We will report the two-sided 95% confidence interval of the estimated relative risk and tabulate occurrence by age at treatment and gender.

hospitalization and/or deaths Similarly, we will report the two-sided 95% confidence interval of the estimated relative risk and tabulate occurrence by age at treatment and gender.

weight comparison of grams per kilogram per day (growth velocity) between treatment and placebo arms, permuting at the level of randomization.

height comparison of millimeters per day (growth velocity) between treatment and placebo arms, permuting at the level of randomization.

weight, height, MUAC, WHZ, WAZ, HAZ ANCOVA comparing outcomes between treatment and placebo arms using baseline as a covariate.

neonatal mortality Binomial regression with complementary log-log link and firth correction used to ensure numerical stability. The analysis will be **two-sided**, with a **type I error rate** (alpha) of 0.05, analyzed identically to the primary outcome.

12-month mortality Binomial regression with complementary log-log link and firth correction used to ensure numerical stability. The analysis will be **two-sided**, with a **type I error rate** (alpha) of 0.05, analyzed identically to the primary outcome.

cause-specific mortality Leveraging verbal autopsy information, specific causes of death will be analyzed as **binomial regression** with complementary log-log link. It will be **two-sided**, with a **type I error rate** (alpha) of 0.05.

2.2 Interim Monitoring

Efficacy. Interim analysis will be executed by the trial biostatistician at the central site. A single interim analysis conducted at alpha of 0.001 will be conducted. Specifically, the interim analysis will be conducted when full data are available for the first third of patients (six months after the last individual in the first third of patients) or at the end of the first full year, whichever occurs first.

Futility. An interim analysis of futility is proposed, to be finalized in consultation with the Data and Safety Monitoring Board. Specifically, we propose to conduct the same regression as in the primary analysis comparing the treatment and control arms using only data from the first year. This analysis will be conducted using simulation. We suggest consideration of the conditional power to detect a 30% effect. If this drops below 10% at the interim analysis, discontinuation of the trial or other changes to the protocol may be made in consultation with the Data and Safety Monitoring Board.

Safety. Pyloric stenosis is a rare, though serious, outcome. Cases will be tabulated, and the proportion experiencing this outcome by treatment status will be reported on a quarterly basis, along with listing of symptoms, onset date, and surgical outcomes once performed. We will cross-tabulate by age and gender. Statistical tests will be reported, but the decision to discontinue for safety is not expected to be made based solely on statistical considerations, but rather in conjunction with the DSMC.

We will summarize adverse events overall and separately by study arm. We will report adverse events by study arm to the DSMC at interim meetings throughout the trial, but primary investigators will be masked to the arm-stratified results until the primary outcome analysis is unmasked at the end of the trial. We will summarize adverse events (numerator) by children at risk (denominator) and will disaggregate and report separately more common events following best practice recommendations [LBM+16]. We will estimate the risk difference in adverse events between arms and 95 percent confidence interval for the difference, though we acknowledge that the trial is not necessarily powered to detect differences in adverse events.

3 Sample Size Considerations

We use the following formula:

$$n = \frac{p_0(1-p_0) + p_1(1-p_1)}{(p_1-p_0)^2}\left(z_{1-\alpha/2} - z_{1-\beta}\right)^2$$

We assume a mortality probability of 0.035. We also assume a reduction in the probability of mortality, this reduction being 20%. Specifically, the mortality in the azithromycin group is one minus this probability, multiplied by the placebo mortality probability; an effect size expressed by 10% corresponds to multiplying the baseline probability by 1-10% = 1-0.1 = 0.9.

mortality probability assumed 0.035 (over the study period of six months).

effect size assumed to be 20%.

loss to follow-up assumed to be 10%.

The proposed sample size per arm is 10856, for a total of 21712 in both arms.

Sensitivity analysis, 20% effect size

- **mortality rate** 25 per thousand, **loss to follow up** 10%: total enrollment 30682.
- **mortality rate** 35 per thousand, **loss to follow up** 20%: total enrollment 24426.

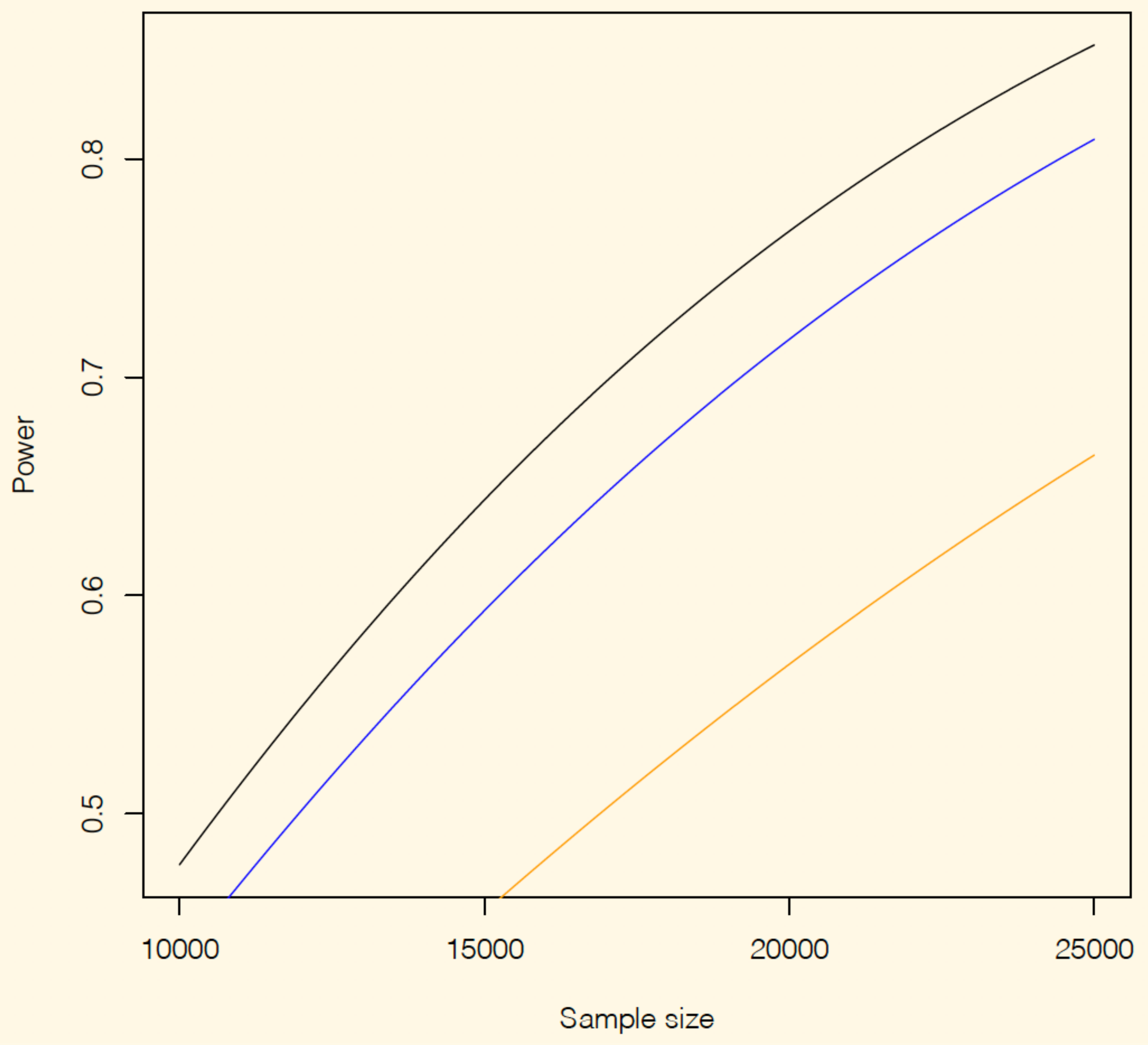

Power for secondary outcomes

All analyses assume a two-sided alpha of 0.05 and a power of 0.8.

nutritional status The proposed sample size will be sufficient to detect a standardized effect size of 0.04 for weight and height.

pyloric stenosis The proposed sample size will be sufficient to detect a relative risk of approximately 2.1.

4 Randomization

The randomization will be conducted using R. The function sample with option replace=FALSE will be used to conduct the random shuffling. Note that the choice of the random number seed completely determines the randomization. To ensure the integrity of the randomization, we will use the procedure we used for MORDOR/Malawi.

5 Abbreviations

ANCOVA Analysis of Covariance

DSMC Data and Safety Monitoring Committee

HAZ Height for Age Z score

HDSS Health and Demographic Surveillance System

LAZ Length for Age Z score

MUAC Mid Upper Arm Circumference

SAP Statistical Analysis Plan

WAZ Weight for Age Z score

WHZ Weight for Height Z score

WLZ Weight for Length Z score

References

[LBM+16] N. Lineberry, J. A. Berlin, B. Mansi, S. Glasser, M. Berkwits, C. Klem, and et al. Recommendations to improve adverse event reporting in clinical trial publications: a joint pharmaceutical industry/journal editor perspective. *British Medical Journal*, 355, 2016.

[SL12] S. Singh and Y. K. Loke. Drug safety assessment in clinical trials: methodological challenges and opportunities. *Trials*, 13, 2012.

6 Revision History

26 Sep 2018 Sample size formula revision. A pre-specified secondary analysis pooling infants randomized in the neonate and individual studies was added.

28 Feb 2019 Revisions to interim, futility, and safety analyses according to Sep 2018 DSMC recommendations. Addition of secondary endpoints: cause-specific mortality, 12- month mortality, and neonatal mortality.

26 Mar 2019 Added pre-specifications of follow-up date time window and sensitivity analysis for primary outcome.

19 Apr 2019 Clarification regarding unplanned analyses and use of multiple comparison corrections.

26 Jul 2019 Changed mortality analyses from logistic regression to binomial with complementary log-log link.

14 Oct 2019 Added comment that deaths on enrollment day contribute 0.5 days person time.

28 Oct 2019 Revision to futility analysis and secondary outcomes.

09 Dec 2019 Added adverse event detail to the safety component of interim analysis.

16 Jan 2020 Abbreviated the study name on title page.

07 May 2020 Specified WHO standard range for inclusion for anthropometric (child growth) analysis.

21 July 2020 Added secondary analysis of negative binomial regression on clinic visit counts.

14 April 2021 Added prespecified subgroup analyses and aggregate adverse event reporting under additional secondary outcomes.

14 May 2021 Defined subgroup analyses for birthweight, growth standards and regions.

9 June 2021 Updated regions for subgroup analyses, added LAZ and WLZ abbreviation definitions

10 June 2021 Added a breakdown of the reasons for visiting the clinic under additional secondary outcomes
