# Supplementary material for: Infant mortality and growth failure after oral azithromycin among low birthweight and underweight neonates: A subgroup analysis of a randomized controlled trial
Source: PLOS Glob Public Health. 2023 May 15;3(5):e0001009. doi: 10.1371/journal.pgph.0001009 (PMC10184901; doi:10.1371/journal.pgph.0001009)

**S1 Fig.**  Venn diagram showing overlap in baseline anthropometric deficits defined by low birthweight (LBW; <2500 g), low weight-for-age Z-score (WAZ < -2), low weight-for-length Z-score (WLZ < -2), and low mid-upper arm circumference (MUAC < 11 cm).


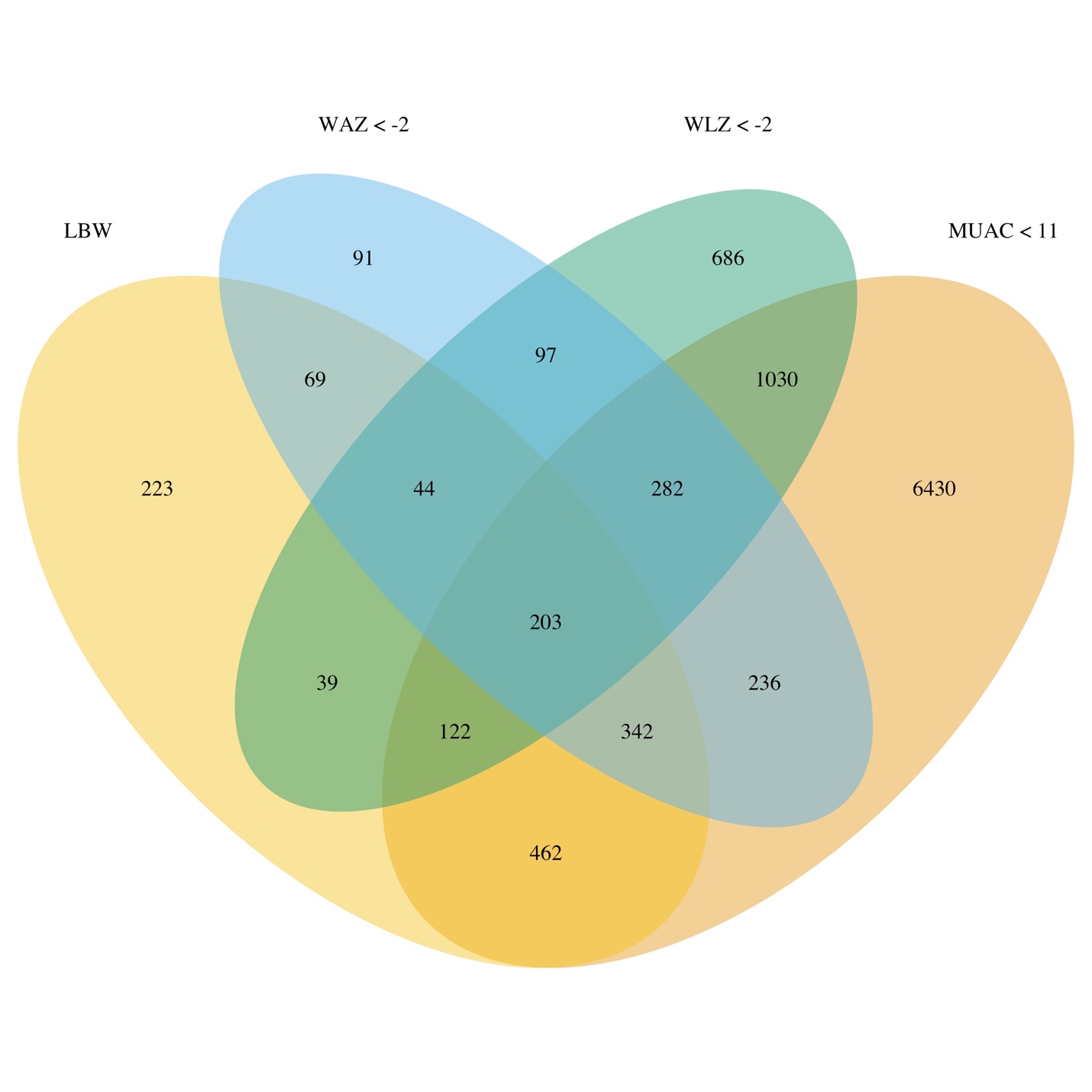

Supplement: S1 Fig — (DOCX) [file pgph.0001009.s004.docx]
