# Supplementary material for: Infant mortality and growth failure after oral azithromycin among low birthweight and underweight neonates: A subgroup analysis of a randomized controlled trial
Source: PLOS Glob Public Health. 2023 May 15;3(5):e0001009. doi: 10.1371/journal.pgph.0001009 (PMC10184901; doi:10.1371/journal.pgph.0001009)
Supplement: S2 Fig — Outcomes include weight gain (g/day; A), length gain (mm/day, B), mid-upper arm circumference (C), underweight (weight-for-age Z-score -2, D), stunted (length-for-age Z-score <-2), and wasted (weight-for-length Z-score <2) at 6 months. (DOCX) [file pgph.0001009.s005.docx]

**S2 Fig.** Mean differences and odds ratios in subgroups of neonates randomized to azithromycin versus placebo in subgroups defined by low mid-upper arm circumference (< 11.0 cm), severe underweight (WAZ < -3), low MUAC or low WAZ (MUAC < 11.0 or WAZ < -2), low weight-for-length Z-score (WLZ < -2), severe wasting (WLZ < -3), and any anthropometric deficit (birthweight < 2500 g, WAZ < -2, WLZ < -2, or MUAC < 11.0 cm). Outcomes include weight gain (g/day; A), length gain (mm/day, B), mid-upper arm circumference (C), underweight (weight-for-age Z-score -2, D), stunted (length-for-age Z-score <-2), and wasted (weight-for-length Z-score <2) at 6 months.
