# Supplementary material for: Infant mortality and growth failure after oral azithromycin among low birthweight and underweight neonates: A subgroup analysis of a randomized controlled trial
Source: PLOS Glob Public Health. 2023 May 15;3(5):e0001009. doi: 10.1371/journal.pgph.0001009 (PMC10184901; doi:10.1371/journal.pgph.0001009)
Supplement: S1 Table — (DOCX) [file pgph.0001009.s006.docx]

**S1 Table.** Mortality and anthropometric endpoints by subgroup in infants receiving azithromycin versus placebo

|  | **Azithromycin**  **N (%) or**  **Mean (SD)** | **Placebo**  **N (%) or**  **Mean (SD)** | **Mean Difference or Odds Ratio (95% CI)** | **P for interaction** |
| --- | --- | --- | --- | --- |
| ***Mortality*** |  |  |  |  |
| LBW and UW | 4 (1.1%) | 5 (1.4%) | 0.84 (0.21 to 3.19) | 0.86 |
| UW only | 6 (1.4%) | 6 (1.7%) | 0.84 (0.26 to 2.72) |  |
| LBW only | 5 (1.0%) | 2 (0.4%) | 0.44 (0.06 to 2.04) |  |
| Not LBW or UW | 29 (0.3%) | 32 (0.4%) | 0.91 (0.55 to 1.50) |  |
| ***Weight gain (g/day)*** |  |  |  |  |
| LBW and UW | 25.1 (5.7) | 24.6 (5.4) | 0.57 (-0.28 to 1.42) | 0.46 |
| UW only | 25.7 (5.5) | 25.9 (5.5) | -0.22 (-1.04 to 0.60) |  |
| LBW only | 23.0 (5.1) | 23.3 (5.2) | -0.23 (-0.93 to 0.46) |  |
| Not LBW or UW | 23.1 (5.2) | 23.1 (5.4) | -0.05 (-0.21 to 0.12) |  |
| ***Length change (mm/day)*** |  |  |  |  |
| LBW and UW | 0.96 (0.18) | 0.94 (0.17) | 0.02 (-0.01 to 0.04) | 0.46 |
| UW only | 0.94 (0.17) | 0.94 (0.17) | -0.006 (-0.03 to 0.02) |  |
| LBW only | 0.90 (0.17) | 0.89 (0.17) | 0.01 (-0.01 to 0.03) |  |
| Not LBW or UW | 0.88 (0.16) | 0.88 (0.16) | 0.002 (-0.003 to 0.006) |  |
| ***MUAC (cm)*** |  |  |  |  |
| LBW and UW | 13.7 (1.1) | 13.6 (1.1) | 0.08 (-0.08 to 0.2) | 0.84 |
| UW only | 13.7 (1.2) | 13.8 (1.1) | 0.002 (-0.2 to 0.2) |  |
| LBW only | 13.8 (1.2) | 13.8 (1.1) | -0.02 (-0.2 to 0.1) |  |
| Not LBW or UW | 14.1 (1.1) | 14.1 (1.1) | 0.008 (-0.03 to 0.04) |  |
| ***Underweight (WAZ < -2)*** |  |  |  |  |
| LBW and UW | 52 (16.5%) | 80 (23.4%) | 0.65 (0.44 to 0.95) | 0.06 |
| UW only | 71 (18.6%) | 52 (16.0%) | 1.20 (0.81 to 1.79) |  |
| LBW only | 43 (10.5%) | 35 (8.0%) | 1.34 (0.84 to 2.15) |  |
| Not LBW or UW | 480 (5.9%) | 467 (5.6%) | 1.05 (0.92 to 1.19) |  |
| ***Stunted (HAZ < -2)*** |  |  |  |  |
| LBW and UW | 80 (25.4%) | 99 (29.0%) | 0.84 (0.59 to 1.18) | 0.35 |
| UW only | 70 (18.4%) | 48 (14.8%) | 1.30 (0.87 to 1.95) |  |
| LBW only | 56 (13.7%) | 64 (14.7%) | 0.92 (0.62 to 1.35) |  |
| Not LBW or UW | 663 (8.1%) | 638 (7.7%) | 1.06 (0.95 to 1.19) |  |
| ***Wasted (WHZ < -2)*** |  |  |  |  |
| LBW and UW | 30 (9.5%) | 35 (10.2%) | 0.92 (0.55 to 1.54) | 0.96 |
| UW only | 37 (9.7%) | 31 (9.5%) | 1.02 (0.62 to 1.69) |  |
| LBW only | 25 (6.1%) | 31 (7.1%) | 0.85 (0.49 to 1.46) |  |
| Not LBW or UW | 424 (5.2%) | 447 (5.4%) | 0.96 (0.84 to 1.10) |  |
