# Supplementary material for: Infant mortality and growth failure after oral azithromycin among low birthweight and underweight neonates: A subgroup analysis of a randomized controlled trial
Source: PLOS Glob Public Health. 2023 May 15;3(5):e0001009. doi: 10.1371/journal.pgph.0001009 (PMC10184901; doi:10.1371/journal.pgph.0001009)
Supplement: S2 Table — (DOCX) [file pgph.0001009.s007.docx]

**S2 Table.** Baseline demographic characteristics by non-prespecified subgroups defined by anthropometric deficits

|  | Low MUAC  (< 110 mm) | | Severe underweight (WAZ < -3) | | Low MUAC  or WAZ < - 2 | | Wasting  (WLZ < -2) | | Severe wasting  (WLZ < -3) | | Any anthropometric deficit* | |
| --- | --- | --- | --- | --- | --- | --- | --- | --- | --- | --- | --- | --- |
|  | Azithro | Placebo | Azithro | Placebo | Azithro | Placebo | Azithro | Placebo | Azithro | Placebo | Azithro | Placebo |
| N | 5,326 | 5,120 | 72 | 82 | 5,509 | 5,299 | 1,438 | 1,400 | 410 | 366 | 6,016 | 5,873 |
| Chronological age, days |  |  |  |  |  |  |  |  |  |  |  |  |
| Mean (SD) | 11.8 (4.4) | 11.7 (4.4) | 24.4 (2.4) | 24.4 (2.4) | 12.0 (4.5) | 11.9 (4.6) | 12.3 (4.9) | 12.0 (4.7) | 12.5 (5.1) | 12.2 (4.7) | 12.1 (4.7) | 12.0 (4.6) |
| Sex |  |  |  |  |  |  |  |  |  |  |  |  |
| Female | 2,755 (51.9%) | 2,730 (53.3%) | 20 (27.8%) | 23 (28.1%) | 2,848 (51.7%) | 2,795 (52.8%) | 747 (52.0%) | 743 (53.1%) | 206 (50.2%) | 186 (50.8%) | 3,130 (52.0%) | 3,106 (52.9%) |
| Male | 2,560 (48.1%) | 2,390 (46.7%) | 52 (72.2%) | 59 (72.0%) | 2,661 (48.3%) | 2,504 (47.3%) | 691 (48.1%) | 657 (46.9%) | 204 (49.8%) | 180 (49.2%) | 2,886 (48.0%) | 2,767 (47.1%) |
| Mother’s age, years |  |  |  |  |  |  |  |  |  |  |  |  |
| Mean (SD) | 25.8 (6.3) | 25.6 (6.2) | 25.0 (6.5) | 25.6 (7.4) | 25.8 (6.3) | 25.6 (6.3) | 25.9 (6.3) | 25.6 (6.4) | 26.1 (6.3) | 25.6 (6.1) | 25.8 (6.3) | 25.6 (6.2) |
| Birthweight, g |  |  |  |  |  |  |  |  |  |  |  |  |
| Mean (SD) | 2873 (377) | 2867 (394) | 2324 (358) | 2319 (313) | 2862 (380) | 2856 (396) | 2795 (379) | 2792 (394) | 2778 (384) | 2763 (406) | 2859 (387) | 2852 (402) |
| Weight at enrollment, kg |  |  |  |  |  |  |  |  |  |  |  |  |
| Mean (SD) | 3.1 (0.4) | 3.1 (0.4) | 2.6 (0.07) | 2.6 (0.08) | 3.1 (0.4) | 3.1 (0.4) | 2.9 (0.3) | 2.9 (0.4) | 2.8 (0.3) | 2.9 (0.4) | 3.1 (0.4) | 3.1 (0.4) |
| Length at enrollment, cm |  |  |  |  |  |  |  |  |  |  |  |  |
| Mean (SD) | 50.1 (1.9) | 50.1 (1.9) | 48.4 (2.4) | 48.3 (1.7) | 50.1 (1.9) | 50.1 (1.9) | 51.6 (2.0) | 51.6 (2.1) | 52.4 (2.2) | 52.6 (2.5) | 50.2 (2.0) | 50.3 (2.1) |
| WAZ |  |  |  |  |  |  |  |  |  |  |  |  |
| Mean (SD) | -0.95 (0.9) | -0.95 (0.9) | -3.3 (0.2) | -3.2 (0.2) | -1.0 (0.9) | -1.0 (0.9) | -1.5 (0.8) | -1.4 (0.8) | -1.7 (0.7) | -1.6 (0.8) | -1.0 (0.9) | -1.0 (0.9) |
| WLZ |  |  |  |  |  |  |  |  |  |  |  |  |
| Mean (SD) | -0.92 (1.3) | -0.93 (1.3) | -2.1 (1.3) | -1.8 (1.1) | -0.95 (1.27) | -0.96 (1.24) | -2.8 (0.7) | -2.8 (0.7) | -3.7 (0.7) | -3.7 (0.8) | -1.1 (1.3) | -1.1 (1.3) |
| LAZ |  |  |  |  |  |  |  |  |  |  |  |  |
| Mean (SD) | -0.73 (1.0) | -0.73 (1.0) | -2.7 (1.3) | -2.7 (0.9) | -0.77 (1.0) | -0.76 (1.0) | 0.0 (1.1) | 0.05 (1.2) | 0.4 (1.2) | 0.6 (1.4) | -0.7 (1.1) | -0.7 (1.1) |

*Low birthweight or WAZ < -2 or WLZ < -2 or MUAC < 110)
