# Supplementary material for: Infant mortality and growth failure after oral azithromycin among low birthweight and underweight neonates: A subgroup analysis of a randomized controlled trial
Source: PLOS Glob Public Health. 2023 May 15;3(5):e0001009. doi: 10.1371/journal.pgph.0001009 (PMC10184901; doi:10.1371/journal.pgph.0001009)
Supplement: S3 Table — (DOCX) [file pgph.0001009.s008.docx]

**S3 Table.** Mortality and anthropometric endpoints by subgroup in infants defined by mid-upper arm circumference (MUAC; <11.0 cm or ≥11.0 cm) receiving azithromycin versus placebo

|  | **Azithromycin**  **N (%) or**  **Mean (SD)** | **Placebo**  **N (%) or**  **Mean (SD)** | **Mean Difference or Odds Ratio (95% CI)** | **P for interaction** |
| --- | --- | --- | --- | --- |
| ***Mortality*** |  |  |  |  |
| MUAC < 11.0 cm | 29 (0.54%) | 33 (0.64%) | 0.84 (0.51 to 1.39) | 0.99 |
| MUAC ≥ 11.0 cm | 12 (0.23%) | 15 (0.27%) | 0.84 (0.38 to 1.79) |  |
| ***Weight gain (g/day)*** |  |  |  |  |
| MUAC < 11.0 cm | 23.5 (5.2) | 23.6 (5.3) | -0.06 (-0.28 to 0.15) | 0.64 |
| MUAC ≥ 11.0 cm | 22.9 (5.4) | 22.9 (5.6) | 0.008 (-0.21 to 0.23) |  |
| ***Length change (mm/day)*** |  |  |  |  |
| MUAC < 11.0 cm | 0.90 (0.15) | 0.90 (0.15) | 0.002 (-0.004 to 0.008) | 0.94 |
| MUAC ≥ 11.0 cm | 0.87 (0.16) | 0.87 (0.17) | 0.002 (-0.004 to 0.009) |  |
| ***MUAC (cm)*** |  |  |  |  |
| MUAC < 11.0 cm | 13.7 (1.0) | 13.7 (1.0) | 0.006 (-0.04 to 0.05) | 0.78 |
| MUAC ≥ 11.0 cm | 14.4 (1.2) | 14.4 (1.2) | 0.02 (-0.03 to 0.06) |  |
| ***Underweight (WAZ < -2)*** |  |  |  |  |
| MUAC < 11.0 cm | 380 (8.2%) | 383 (8.5%) | 0.96 (0.83 to 1.12) | 0.23 |
| MUAC ≥ 11.0 cm | 261 (5.7%) | 249 (5.1%) | 1.11 (0.93 to 1.33) |  |
| ***Stunted (HAZ < -2)*** |  |  |  |  |
| MUAC < 11.0 cm | 457 (9.9%) | 436 (9.7%) | 1.02 (0.89 to 1.18) | 0.93 |
| MUAC ≥ 11.0 cm | 404 (8.8%) | 413 (8.5%) | 1.03 (0.90 to 1.19) |  |
| ***Wasted (WHZ < -2)*** |  |  |  |  |
| MUAC < 11.0 cm | 285 (6.2%) | 313 (7.0%) | 0.88 (0.75 to 1.04) | 0.14 |
| MUAC ≥ 11.0 cm | 230 (5.0%) | 228 (4.7%) | 1.07 (0.88 to 1.29) |  |
