# Supplementary material for: Infant mortality and growth failure after oral azithromycin among low birthweight and underweight neonates: A subgroup analysis of a randomized controlled trial
Source: PLOS Glob Public Health. 2023 May 15;3(5):e0001009. doi: 10.1371/journal.pgph.0001009 (PMC10184901; doi:10.1371/journal.pgph.0001009)
Supplement: S4 Table — (DOCX) [file pgph.0001009.s009.docx]

**S4 Table.** Mortality and anthropometric endpoints by subgroup in infants defined by severe underweight (WAZ < -3) or not severely underweight (WAZ ≥ -3) receiving azithromycin versus placebo

|  | **Azithromycin**  **N (%) or**  **Mean (SD)** | **Placebo**  **N (%) or**  **Mean (SD)** | **Mean Difference or Odds Ratio (95% CI)** | **P for interaction** |
| --- | --- | --- | --- | --- |
| ***Mortality*** |  |  |  |  |
| WAZ < -3 | 2 (2.8%) | 2 (2.4%) | 1.14 (0.13 to 9.67) | 0.78 |
| WAZ ≥ -3 | 39 (0.37%) | 46 (0.43%) | 0.85 (0.55 to 1.30) |  |
| ***Weight gain (g/day)*** |  |  |  |  |
| WAZ < -3 | 27.0 (5.4) | 26.0 (6.5) | 0.96 (-1.20 to 3.12) | 0.31 |
| WAZ ≥ -3 | 23.2 (5.3) | 23.2 (5.4) | -0.03 (-0.18 to 0.13) |  |
| ***Length change (mm/day)*** |  |  |  |  |
| WAZ < -3 | 1.0 (0.2) | 0.9 (0.2) | 0.06 (-0.01 to 0.13) | 0.04 |
| WAZ ≥ -3 | 0.9 (0.2) | 0.9 (0.2) | 0.002 (-0.002 to 0.007) |  |
| ***MUAC (cm)*** |  |  |  |  |
| WAZ < -3 | 13.6 (1.0) | 13.6 (1.4) | 0.02 (-0.41 to 0.45) | 0.95 |
| WAZ ≥ -3 | 14.1 (1.2) | 14.1 (1.1) | 0.01 (-0.02 to 0.04) |  |
| ***Underweight (WAZ < -2)*** |  |  |  |  |
| WAZ < -3 | 10 (17.2%) | 21 (32.8%) | 0.43 (0.17 to 0.99) | 0.04 |
| WAZ ≥ -3 | 636 (6.9%) | 614 (6.6%) | 1.05 (0.94 to 1.18) |  |
| ***Stunted (HAZ < -2)*** |  |  |  |  |
| WAZ < -3 | 12 (20.7%) | 30 (46.9%) | 0.30 (0.13 to 0.65) | 0.002 |
| WAZ ≥ -3 | 857 (9.3%) | 821 (8.8%) | 1.06 (0.96 to 1.18) |  |
| ***Wasted (WHZ < -2)*** |  |  |  |  |
| WAZ < -3 | 4 (6.9%) | 6 (9.4%) | 0.72 (0.18 to 2.64) | 0.66 |
| WAZ ≥ -3 | 512 (5.6%) | 538 (5.8%) | 0.96 (0.85 to 1.09) |  |
