# Supplementary material for: Infant mortality and growth failure after oral azithromycin among low birthweight and underweight neonates: A subgroup analysis of a randomized controlled trial
Source: PLOS Glob Public Health. 2023 May 15;3(5):e0001009. doi: 10.1371/journal.pgph.0001009 (PMC10184901; doi:10.1371/journal.pgph.0001009)
Supplement: S5 Table — (DOCX) [file pgph.0001009.s010.docx]

**S5 Table.** Mortality and anthropometric endpoints by subgroup in infants defined by low mid-upper arm circumference OR underweight (MUAC < 11.0 cm or WAZ < - 2) or neither low mid-upper arm circumference or underweight receiving azithromycin versus placebo

|  | **Azithromycin**  **N (%) or**  **Mean (SD)** | **Placebo**  **N (%) or**  **Mean (SD)** | **Mean Difference or Odds Ratio (95% CI)** | **P for interaction** |
| --- | --- | --- | --- | --- |
| ***Mortality*** |  |  |  |  |
| MUAC < 11 or WAZ < -2 | 31 (0.56%) | 35 (0.66%) | 0.85 (0.52 to 1.38) | 0.92 |
| MUAC ≥ 11 and WAZ ≥ -2 | 10 (0.20%) | 13 (0.24%) | 0.81 (0.34 to 1.84) |  |
| ***Weight gain (g/day)*** |  |  |  |  |
| MUAC < 11 or WAZ < -2 | 23.6 (5.2) | 23.6 (5.3) | -0.05 (-0.27 to 0.16) | 0.77 |
| MUAC ≥ 11 and WAZ ≥ -2 | 22.8 (5.4) | 22.8 (5.5) | -0.008 (-0.23 to 0.22) |  |
| ***Length change (mm/day)*** |  |  |  |  |
| MUAC < 11 or WAZ < -2 | 0.9 (0.2) | 0.9 (0.2) | 0.002 (-0.004 to 0.008) | 0.93 |
| MUAC ≥ 11 and WAZ ≥ -2 | 0.9 (0.2) | 0.9 (0.2) | 0.002 (-0.004 to 0.009) |  |
| ***MUAC (cm)*** |  |  |  |  |
| MUAC < 11 or WAZ < -2 | 13.8 (1.1) | 13.7 (1.0) | 0.009 (-0.03 to 0.05) | 0.92 |
| MUAC ≥ 11 and WAZ ≥ -2 | 14.4 (1.2) | 14.4 (1.2) | 0.01 (-0.03 to 0.06) |  |
| ***Underweight (WAZ < -2)*** |  |  |  |  |
| MUAC < 11 or WAZ < -2 | 407 (8.6%) | 412 (8.9%) | 0.96 (0.83 to 1.11) | 0.19 |
| MUAC ≥ 11 and WAZ ≥ -2 | 235 (5.3%) | 221 (4.7%) | 1.13 (0.93 to 1.36) |  |
| ***Stunted (HAZ < -2)*** |  |  |  |  |
| MUAC < 11 or WAZ < -2 | 490 (10.3%) | 466 (10.0%) | 1.03 (0.90 to 1.18) | 0.99 |
| MUAC ≥ 11 and WAZ ≥ -2 | 373 (8.4%) | 383 (8.1%) | 1.03 (0.89 to 1.19) |  |
| ***Wasted (WHZ < -2)*** |  |  |  |  |
| MUAC < 11 or WAZ < -2 | 298 (6.3%) | 331 (7.1%) | 0.87 (0.74 to 1.03) | 0.08 |
| MUAC ≥ 11 and WAZ ≥ -2 | 217 (4.9%) | 211 (4.5%) | 1.09 (0.90 to 1.32) |  |
