# Supplementary material for: Infant mortality and growth failure after oral azithromycin among low birthweight and underweight neonates: A subgroup analysis of a randomized controlled trial
Source: PLOS Glob Public Health. 2023 May 15;3(5):e0001009. doi: 10.1371/journal.pgph.0001009 (PMC10184901; doi:10.1371/journal.pgph.0001009)
Supplement: S6 Table — (DOCX) [file pgph.0001009.s011.docx]

**S6 Table.** Mortality and anthropometric endpoints by subgroup in infants defined by low weight-for-length Z-score (WLZ < -2) or normal weight-for-length Z-score (WLZ ≥ - 2) receiving azithromycin versus placebo

|  | **Azithromycin**  **N (%) or**  **Mean (SD)** | **Placebo**  **N (%) or**  **Mean (SD)** | **Mean Difference or Odds Ratio (95% CI)** | **P for interaction** |
| --- | --- | --- | --- | --- |
| ***Mortality*** |  |  |  |  |
| WLZ < -2 | 13 (0.90%) | 14 (0.93%) | 0.96 (0.44 to 2.11) | 0.65 |
| WLZ ≥ -2 | 27 (0.29%) | 35 (0.38%) | 0.78 (0.47 to 1.28) |  |
| ***Weight gain (g/day)*** |  |  |  |  |
| WLZ < -2 | 24.3 (5.4) | 24.5 (5.3) | -0.13 (-0.55 to 0.29) | 0.64 |
| WLZ ≥ -2 | 23.1 (5.3) | 23.1 (5.4) | -0.02 (-0.19 to 0.15) |  |
| ***Length change (mm/day)*** |  |  |  |  |
| WLZ < -2 | 0.8 (0.2) | 0.8 (0.2) | 0.006 (-0.007 to 0.02) | 0.54 |
| WLZ ≥ -2 | 0.9 (0.2) | 0.9 (0.2) | 0.002 (-0.003 to 0.007) |  |
| ***MUAC (cm)*** |  |  |  |  |
| WLZ < -2 | 13.8 (1.1) | 13.9 (1.1) | -0.009 (-0.10 to 0.08) | 0.71 |
| WLZ ≥ -2 | 14.1 (1.2) | 14.1 (1.1) | 0.01 (-0.02 to 0.04) |  |
| ***Underweight (WAZ < -2)*** |  |  |  |  |
| WLZ < -2 | 139 (11.0%) | 127 (10.2%) | 1.08 (0.84 to 1.40) | 0.67 |
| WLZ ≥ -2 | 504 (6.3%) | 506 (6.2%) | 1.02 (0.90 to 1.16) |  |
| ***Stunted (HAZ < -2)*** |  |  |  |  |
| WLZ < -2 | 116 (9.2%) | 103 (8.3%) | 1.12 (0.85 to 1.48) | 0.58 |
| WLZ ≥ -2 | 743 (9.3%) | 741 (9.1%) | 1.03 (0.92 to 1.14) |  |
| ***Wasted (WHZ < -2)*** |  |  |  |  |
| WLZ < -2 | 152 (12.0%) | 132 (10.7%) | 1.15 (0.90 to 1.47) | 0.09 |
| WLZ ≥ -2 | 364 (4.6%) | 412 (5.1%) | 0.90 (0.78 to 1.04) |  |
