# Supplementary material for: Infant mortality and growth failure after oral azithromycin among low birthweight and underweight neonates: A subgroup analysis of a randomized controlled trial
Source: PLOS Glob Public Health. 2023 May 15;3(5):e0001009. doi: 10.1371/journal.pgph.0001009 (PMC10184901; doi:10.1371/journal.pgph.0001009)
Supplement: S7 Table — (DOCX) [file pgph.0001009.s012.docx]

**S7 Table.** Mortality and anthropometric endpoints by subgroup in infants defined by severe wasting (WLZ < -3) or WLZ ≥ -3 receiving azithromycin versus placebo

|  | **Azithromycin**  **N (%) or**  **Mean (SD)** | **Placebo**  **N (%) or**  **Mean (SD)** | **Mean Difference or Odds Ratio (95% CI)** | **P for interaction** |
| --- | --- | --- | --- | --- |
| ***Mortality*** |  |  |  |  |
| WLZ < -3 | 8 (1.95%) | 4 (1.09%) | 1.76 (0.55 to 6.64) | 0.18 |
| WLZ ≥ -3 | 32 (0.31%) | 44 (0.43%) | 0.73 (0.46 to 1.15) |  |
| ***Weight gain (g/day)*** |  |  |  |  |
| WLZ < -3 | 24.6 (5.5) | 25.4 (5.7) | -0.74 (-1.59 to 0.12) | 0.09 |
| WLZ ≥ -3 | 23.1 (5.3) | 23.2 (5.4) | -0.009 (-0.17 to 0.15) |  |
| ***Length change (mm/day)*** |  |  |  |  |
| WLZ < -3 | 0.8 (0.2) | 0.8 (0.2) | -0.008 (-0.04 to 0.02) | 0.39 |
| WLZ ≥ -3 | 0.9 (0.2) | 0.9 (0.2) | 0.003 (-0.001 to 0.008) |  |
| ***MUAC (cm)*** |  |  |  |  |
| WLZ < -3 | 13.8 (1.2) | 14.0 (1.1) | -0.11 (-0.29 to 0.06) | 0.17 |
| WLZ ≥ -3 | 14.1 (1.2) | 14.1 (1.1) | 0.01 (-0.02 to 0.04) |  |
| ***Underweight (WAZ < -2)*** |  |  |  |  |
| WLZ < -3 | 37 (10.8%) | 35 (11.2%) | 0.97 (0.59 to 1.58) | 0.79 |
| WLZ ≥ -3 | 606 (6.8%) | 598 (6.6%) | 1.04 (0.92 to 1.15) |  |
| ***Stunted (HAZ < -2)*** |  |  |  |  |
| WLZ < -3 | 36 (10.5%) | 17 (5.4%) | 2.06 (1.15 to 3.82) | 0.02 |
| WLZ ≥ -3 | 823 (9.3%) | 827 (9.1%) | 1.02 (0.92 to 1.12) |  |
| ***Wasted (WHZ < -2)*** |  |  |  |  |
| WLZ < -3 | 42 (12.3%) | 31 (9.9%) | 1.28 (0.78 to 2.10) | 0.23 |
| WLZ ≥ -3 | 474 (5.3%) | 513 (5.7%) | 0.94 (0.83 to 1.07) |  |
