# Supplementary material for: Infant mortality and growth failure after oral azithromycin among low birthweight and underweight neonates: A subgroup analysis of a randomized controlled trial
Source: PLOS Glob Public Health. 2023 May 15;3(5):e0001009. doi: 10.1371/journal.pgph.0001009 (PMC10184901; doi:10.1371/journal.pgph.0001009)
Supplement: S8 Table — (DOCX) [file pgph.0001009.s013.docx]

**S8 Table.** Mortality and anthropometric endpoints by subgroup in infants defined by any anthropometric deficit (low birthweight or WAZ < - 2 or WLZ < -2 or MUAC < 11) versus no anthropometric deficit at enrollment

|  | **Azithromycin**  **N (%) or**  **Mean (SD)** | **Placebo**  **N (%) or**  **Mean (SD)** | **Mean Difference or Odds Ratio (95% CI)** | **P for interaction** |
| --- | --- | --- | --- | --- |
| ***Mortality*** |  |  |  |  |
| Anthropometric deficit | 33 (0.55%) | 37 (0.63%) | 0.87 (0.54 to 1.39) | 0.79 |
| No anthropometric deficit | 8 (0.17%) | 11 (0.23%) | 0.75 (0.29 to 1.87) |  |
| ***Weight gain (g/day)*** |  |  |  |  |
| Anthropometric deficit | 23.6 (5.2) | 23.7 (5.3) | -0.12 (-0.33 to 0.08) | 0.18 |
| No anthropometric deficit | 22.8 (5.3) | 22.7 (5.6) | 0.09 (-0.15 to 0.32) |  |
| ***Length change (mm/day)*** |  |  |  |  |
| Anthropometric deficit | 0.9 (0.2) | 0.9 (0.2) | 0.003 (-0.003 to 0.009) | 0.92 |
| No anthropometric deficit | 0.9 (0.2) | 0.9 (0.2) | 0.002 (-0.005 to 0.009) |  |
| ***MUAC (cm)*** |  |  |  |  |
| Anthropometric deficit | 13.8 (1.1) | 13.8 (1.1) | -0.0004 (-0.04 to 0.04) | 0.51 |
| No anthropometric deficit | 14.4 (1.2) | 14.4 (1.2) | 0.02 (-0.03 to 0.07) |  |
| ***Underweight (WAZ < -2)*** |  |  |  |  |
| Anthropometric deficit | 449 (8.6%) | 442 (8.6%) | 1.01 (0.88 to 1.16) | 0.66 |
| No anthropometric deficit | 194 (4.8%) | 191 (4.6%) | 1.07 (0.88 to 1.31) |  |
| ***Stunted (HAZ < -2)*** |  |  |  |  |
| Anthropometric deficit | 530 (10.2%) | 523 (10.2%) | 1.00 (0.88 to 1.14) | 0.50 |
| No anthropometric deficit | 334 (8.3%) | 326 (7.8%) | 1.08 (0.92 to 1.26) |  |
| ***Wasted (WHZ < -2)*** |  |  |  |  |
| Anthropometric deficit | 339 (6.5%) | 367 (7.1%) | 0.91 (0.78 to 1.06) | 0.27 |
| No anthropometric deficit | 176 (4.4%) | 175 (4.2%) | 1.05 (0.85 to 1.31) |  |
